# Ultra-sensitive profiling of CRISPR-Cas off-target effects with Tracking-seq2

**DOI:** 10.1038/s41467-026-76778-9

**Published:** 2026-08-11

**Authors:** Tingting Cong, Runda Xu, Xuancheng Chen, Junsong Yuan, Zuomiao Lin, Wenqin Yang, Anji Ju, Liren Wang, Qingcan Wang, Jiqin Zhang, Yinqing Li, Dali Li, Ming Zhu

**Affiliations:** 1 Shanghai Frontiers Science Center of Genome Editing and Cell Therapy, Shanghai Key Laboratory of Regulatory Biology, Institute of Biomedical Sciences and School of Life Sciences, East China Normal University, Shanghai, China; 2 Tsinghua-Peking Joint Center for Life Sciences, Tsinghua University, Beijing, 100084 China

**Keywords:** CRISPR-Cas9 genome editing, Genetic engineering, Gene expression profiling, Double-strand DNA breaks

## Abstract

Accurate detection of off-target activity in primary human cells is crucial for ensuring the safety of gene therapies, yet existing methods often lack sufficient sensitivity. To address this limitation, we develop Tracking-seq2, an advanced technology that integrates exogenous 5′ → 3′ exonuclease treatment and non-homologous end joining (NHEJ) pathway inhibitors with the original Tracking-seq. Tracking-seq2 exhibits enhanced sensitivity in profiling off-target sites of diverse genome editors—including Cas9, Cas12a, cytosine base editors (CBEs), adenine base editors (ABEs), and prime editors (PEs). Critically, Tracking-seq2 is directly applicable to clinically relevant primary human cell types, such as T cells and CD34^+^ hematopoietic stem and progenitor cells (HSPCs). Furthermore, our findings reveal that genomic variations drive distinct off-target heterogeneity across different individuals, highlighting the necessity for personalized safety assessment in clinical genome editing applications. Tracking-seq2 provides a robust platform for sensitive off-target detection in primary cells, with sensitivity comparable to or exceeding current state-of-the-art methods.

## Abstract (web summary)

Ensuring safe gene editing requires precise detection of unintended DNA edits. Here, authors develop Tracking-seq2, a highly sensitive method to profile off-targets for diverse genome editors, and reveal distinct off-target effects across individuals in primary human cells.

## Introduction

CRISPR-based gene editing has significantly advanced the treatment of genetic disorders^1^. The approval of Casgevy for sickle cell disease and β-thalassemia marks a major milestone^2^, with several other CRISPR-based therapeutics now in advanced stages of clinical trials. Beyond standard Cas9, CRISPR-derived editors, such as base editors (BEs) and prime editors (PEs), also hold significant therapeutic promise and are nearing clinical use^3,4^. However, unintended off-target DNA damage remains a primary safety concern, and our prior studies have revealed significant off-target heterogeneity across editor modalities and cell types even with identical guide RNAs^5^. Consequently, sensitive direct detection of CRISPR–Cas off-target activity in native systems is essential.

Existing experimental off-target detection methods comprise two primary categories^6,7^: 1) Cell-free methods, which involve genomic DNA extraction for in vitro cleavage^8–10^, exhibit poor clinical translatability due to fundamental discrepancies such as altered Cas9 kinetics in vitro and the lack of epigenetic context, and therefore often suffer from high false-positive rates^11^. 2) Cell-based methods enable direct cellular detection and can better reflect off-target effects in specific cells^12–16^. Most cell-based technologies are confined to Cas9 systems with limited sensitivity. Tracking-seq is a highly versatile cell-based technology that supports direct off-target detection of multiple genome editors^5^, thus its performance enhancement is of great importance for advancing clinical applications.

Replication protein A (RPA) is a single-stranded DNA (ssDNA) binding protein that is involved in both double-strand break (DSB) and single-strand break (SSB) repair pathways^17,18^. The original Tracking-seq technology captures editing-induced DNA lesions by tracking the genome-wide binding patterns of RPA, enabling the direct detection of off-target sites for diverse genome-editing tools. The workflow comprises four major steps: (1) capturing RPA-bound ssDNA using CUT&RUN; (2) removing double-stranded DNA (dsDNA) using HL-dsDNase; (3) performing strand-specific ssDNA library construction; and (4) next-generation sequencing (NGS) coupled with Offtracker bioinformatics analysis. The Offtracker software calculates a normalized “Track score” for each candidate off-target site, which represents the intensity of Tracking-seq signal, and the empirical threshold for a positive site is Track score > 2.

Cells primarily repair DSBs through two competing pathways: homologous recombination (HR) and non-homologous end joining (NHEJ). NHEJ directly ligates broken ends without extensive DNA processing, whereas HR requires extensive end resection driven by endogenous exonucleases^19^, generating long ssDNA intermediates at the DSB ends. These intermediates are rapidly coated by RPA to stabilize the structures and are finally displaced by RAD51^20^. Therefore, the extensive RPA-ssDNA complexes make HR the main source of the signal detected by Tracking-seq for DSB-inducing editors (such as Cas9 and Cas12a). Based on this mechanism, we hypothesized that because HR and NHEJ are mutually competitive, inhibiting NHEJ would force a greater proportion of DSBs into the HR pathway, thereby increasing endogenous ssDNA generation. Additionally, the exogenous addition of a 5′ → 3′ exonuclease could extend the length of end resection. Consequently, combining NHEJ inhibition with exonuclease treatment should amplify the RPA-ssDNA signal and enhance the sensitivity of Tracking-seq for DSB-based editors.

In contrast, BEs and PEs function primarily through SSB repair mechanisms^21,22^. During SSB repair, ssDNA intermediates are formed and coated by RPA to stabilize the local structure and coordinate the recruitment of downstream repair factors^18^. Since NHEJ predominantly processes DSBs, inhibiting the NHEJ pathway is not applicable for SSB-generating editors such as BEs or PEs. Nevertheless, exogenous 5′ → 3′ exonuclease treatment can extend ssDNA regions at nicked DNA sites, thereby amplifying the detection signal of Tracking-seq.

Guided by this mechanistic understanding of distinct DNA repair pathways, we develop Tracking-seq2 by implementing editor-specific optimizations. For DSB-dependent editors such as Cas9 and Cas12a, we integrate 5′ → 3′ exonuclease treatment with inhibition of the NHEJ pathway. For SSB-dependent BEs and PEs, only 5′ → 3′ exonuclease treatment is applied to amplify off-target signals. These refinements enable Tracking-seq2 to achieve substantially improved sensitivity in genome-wide off-target profiling, while functioning effectively within patient-derived primary cells.

## Results

### Exogenous T5 treatment and NHEJ inhibition enhance Cas9 off-target detection

**Fig. 1:**
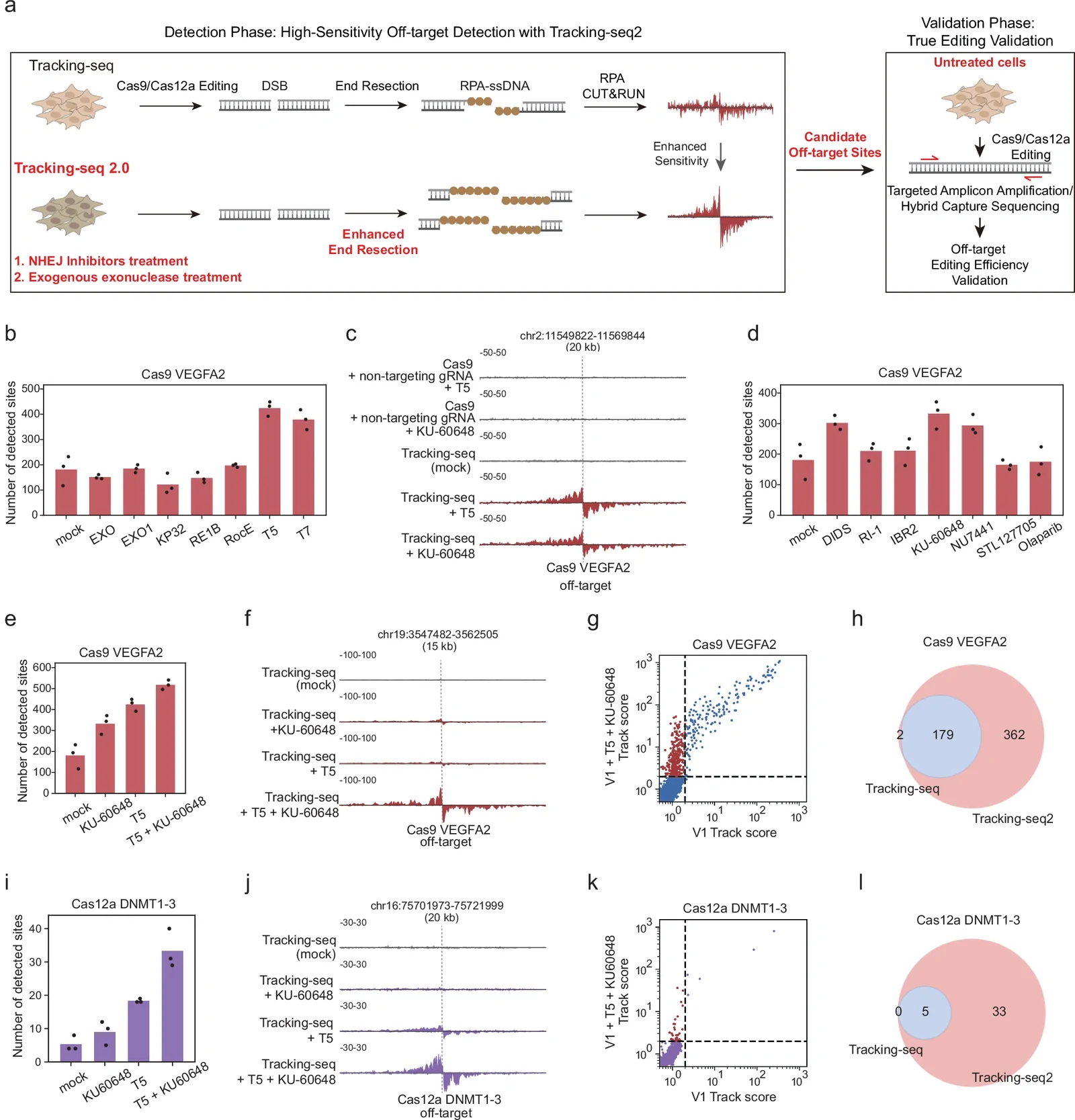
T5 and KU-60648 enhanced the sensitivity of Tracking-seq for detecting Cas9 and Cas12a off-targets in HEK293T cells. **a** Schematic illustration of the Tracking-seq2 workflow for Cas9/Cas12a off-target detection. Exogenous T5 exonuclease treatment and NHEJ inhibition (KU-60648) enhance the detection of Cas9/Cas12a off-target sites by increasing the accumulation of RPA-bound ssDNA intermediates. The Tracking-seq2 workflow consists of two distinct phases: (1) Detection Phase—cells treated with T5 exonuclease and KU-60648 are used for Tracking-seq2; (2) Validation Phase—edited cells without additional treatment are used for targeted deep sequencing. **b** Numbers of off-target sites caused by Cas9 + sgRNA VEGFA2 detected by Tracking-seq co-transfected with plasmids encoding various 5′ → 3′ exonucleases. Mock: cells co-transfected with the PX458 plasmid and an empty vector (pUC19). Each dot represents a biological replicate (*n* = 3). **c** Strand-specific signals generated by Tracking-seq at representative off-target sites (Cas9 + sgRNA VEGFA2). The range denotes normalized signal intensity, represented as read coverage per 10 million mapped reads. **d** Numbers of off-target sites caused by Cas9 + sgRNA VEGFA2 detected by Tracking-seq treated with various chemical inhibitors. Mock: control group without inhibitor treatment. Each dot represents a biological replicate (*n* = 3). **e** Numbers of off-target sites detected by Tracking-seq in various conditions (Cas9 + sgRNA VEGFA2). Each dot represents a biological replicate (*n* = 3). **f** Signals generated by Tracking-seq at representative off-target sites in various conditions. **g** Scatter plot showing the Track scores of Tracking-seq (V1) and Tracking-seq2 (V1 + T5 + KU-60648). Each dot represents a candidate off-target site. Dashed lines represent the thresholds of the Track score > 2 for positive sites. **h** Venn diagram comparing off-target sites (Cas9 + VEGFA2) detected by Tracking-seq and Tracking-seq2. **i** Numbers of off-target sites detected by Tracking-seq in various conditions (Cas12a + sgRNA DNMT1-3). Each dot represents a biological replicate (*n* = 3). **j** Signals generated by Tracking-seq at representative off-target sites in various conditions (Cas12a + sgRNA DNMT1-3). **k** Scatter plot showing the Track scores of Tracking-seq and Tracking-seq2 (Cas12a + sgRNA DNMT1-3). **l** Venn diagram comparing off-target sites detected by Tracking-seq and Tracking-seq2 (Cas12a + sgRNA DNMT1-3). Source data are provided as a Source Data file.

Tracking-seq detects off-target effects via tracking single-stranded DNA (ssDNA) binding patterns of replication protein A (RPA). To test whether exogenous ssDNA resection enzymes could enhance RPA-bound signals, we screened bacterial/phage-derived 5′ → 3′ exonucleases in HEK293T cells (Fig. 1a and Supplementary Fig. 1a). In the experimental groups, cells were co-transfected with the Cas9/sgRNA-expressing PX458 plasmid and a plasmid encoding a specific exonuclease at a 1:1 mass ratio. Cells co-transfected with the PX458 plasmid and an empty vector served as the control group (mock, representing the performance of Tracking-seq V1). This screen revealed that bacteriophage T5 exonuclease (T5) markedly increased the number of off-target sites detected by Tracking-seq (Fig. 1b and Supplementary Fig. 2a), for both sgRNA VEGFA_site_2 (hereinafter referred to as VEGFA2) and sgRNA HEK293_site_4 (hereinafter referred to as HEK4). Many sites from the original Tracking-seq with weak signals or no signals exhibited strong signals and higher Track scores after T5 treatment (Fig. 1c and Supplementary Fig. 2b, c). No signals were observed in control groups treated with T5 alone in the absence of gene editing, confirming that the detected signals were not artifacts (Fig. 1c and Supplementary Fig. 2c).

To identify potential inhibitors that could enhance the performance of Tracking-seq, we screened four NHEJ inhibitors (KU-60648 and NU7441 targeting DNA-PK, STL127705 targeting Ku-70/80, and Olaparib targeting PARP1) and three RAD51 inhibitors (DIDS, RI-1, and IBR2). Following transfection of HEK293T cells with plasmids encoding Cas9 and either sgRNA VEGFA2 or sgRNA HEK4, one of the inhibitors was added to the culture medium and incubated for 48 h. Notably, three inhibitors improved the off-target detection sensitivity of Tracking-seq, with KU-60648 showing the most pronounced effect (Fig. 1d and Supplementary Fig. 2d). Control experiments using Cas9, KU-60648, and a non-targeting sgRNA confirmed that the additional signals were specifically associated with active editing (Fig. 1c and Supplementary Fig. 2c).

We next demonstrated that the combination of T5 and KU-60648 further enhanced the sensitivity of Tracking-seq (Fig. 1e–h and Supplementary Fig. 2e–i), enabling a ~ 2.5-fold increase in the number of detected off-target sites for sgRNA VEGFA2 and a ~ 2-fold increase for sgRNA HEK4 compared to the original method. This integrated approach is termed Tracking-seq2 for DSB-dependent systems.

To confirm that these sites uniquely detected by Tracking-seq2 represented genuine off-target events under physiological conditions rather than artifacts resulting from the modified detection environment, we performed targeted hybrid capture sequencing for all detected off-target sites of VEGFA2 and HEK4, using genomic DNA extracted from edited cells 96 h post-transfection, without T5 exonuclease or NHEJ inhibitor treatment (Fig. 1a). The results showed that the Δindel rates (indel rates of Cas9-edited samples minus indel rates of WT samples) of Tracking-seq2-specific off-target sites were relatively low but detectable (Supplementary Fig. 2j).

To further validate the hybrid capture sequencing results, we performed targeted amplicon sequencing with unique molecular identifiers (UMIs) for off-target sites stratified by editing efficiency (10 sites per tier): 1–10%, 0.1–1%, 0.01–0.1%, and 0.001–0.01% Δindel rates (Supplementary Fig. 3). Amplicon sequencing confirmed genuine editing at all 20 tested sites with 0.1%–10% Δindel rates (Supplementary Fig. 3a, b), and at half of the sites within the 0.001%–0.1% range (Supplementary Fig. 3c-f). Given the ~0.01% median Δindel rate of Tracking-seq2-exclusive sites, we estimate that approximately half of these additional sites represent true off-targets under physiological conditions (without exonuclease or NHEJ inhibitors) (Supplementary Fig. 2j).

Notably, we found that these treatments were also effective for Cas12a, which generates staggered DSB with sticky ends. Application of the combined treatment yielded a ~ 5-fold increase in the number of off-target sites for Cas12a (AsCpf1) editing with sgRNA DNMT1-3 or sgRNA CCR5-1 (Fig. 1i–l and Supplementary Fig. 4a–d), demonstrating that the combined treatment could greatly enhance the off-target detection of DSB-dependent editors.

We further tested Tracking-seq2 with Cas9 using two additional sgRNAs (IDS and VEGFA3), as well as HiFi Cas9^23^ using sgRNA VEGFA2 and sgRNA HEK4. The results showed that Tracking-seq2 detected additional off-target sites for all these conditions (Supplementary Fig. 4e–p). Furthermore, for the same sgRNA, the off-target sites detected with HiFi Cas9 constituted a substantially smaller subset of those detected with WT Cas9 (Supplementary Fig. 4i–p). These results demonstrated that Tracking-seq2 robustly enhanced the detection sensitivity and successfully captured the reduction in off-target activity of HiFi Cas9.

### Comparison of Tracking-seq2 with existing methods

**Fig. 2:**
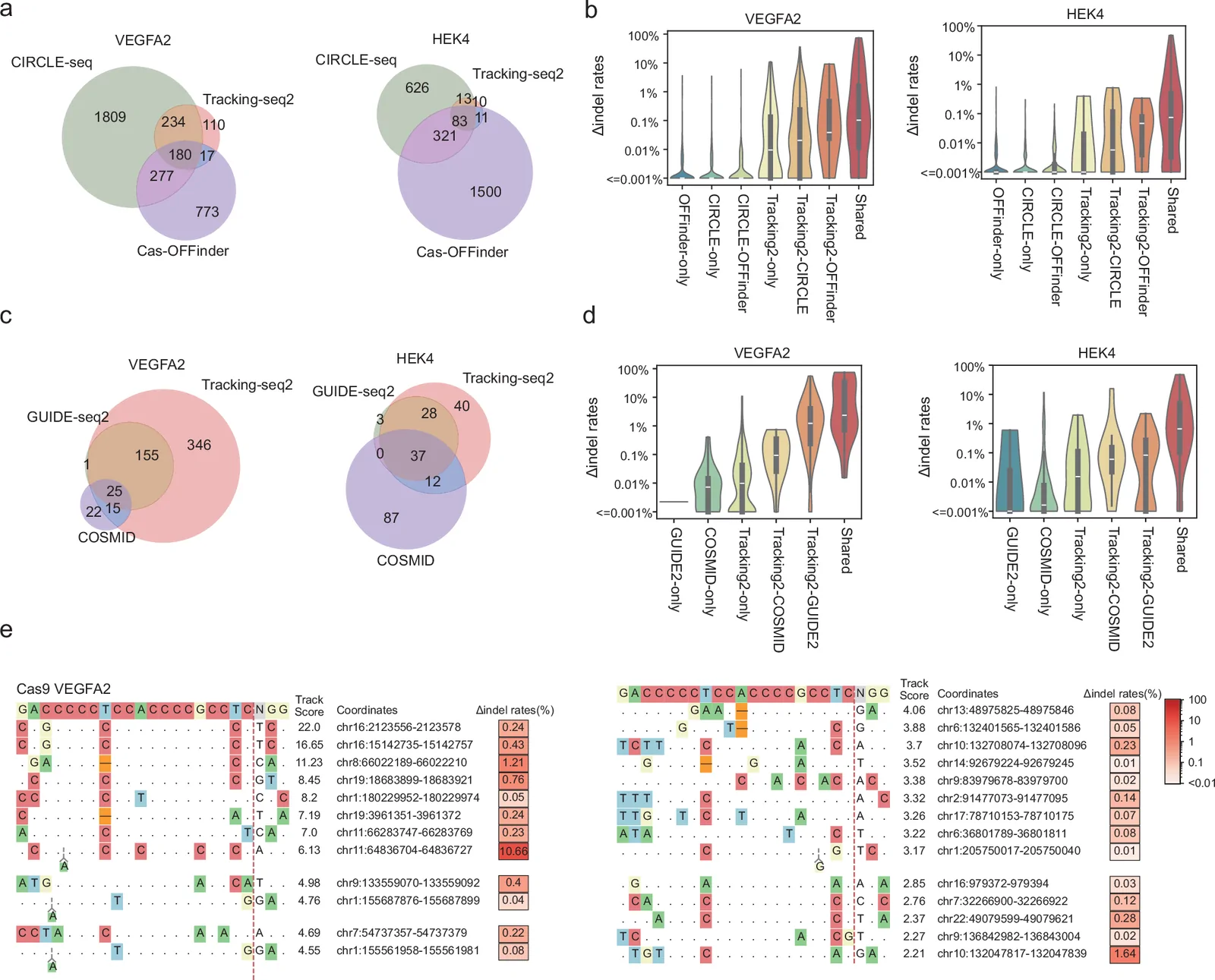
Comparison of Tracking-seq2 with existing cell-free, cell-based and in-silico prediction methods in HEK293T cells. **a** Venn diagrams comparing off-target sites detected (or predicted) by Tracking-seq2, CIRCLE-seq, and CAS-OFFinder for Cas9 + VEGFA2 and Cas9 + HEK4. **b** Violin plot showing the distribution of Δindel rates (edited cells minus unedited cells) measured by hybrid capture targeted sequencing at off-target sites belonging to different sets for Cas9 + VEGFA2 or Cas9 + HEK4. Most off-target sites detected by Tracking-seq2 contained confirmed indels, while most sites exclusively detected by CIRCLE-seq, CAS-OFFinder, or both showed no evidence of indels. Shared: sites shared by all three methods. Each inner box plot shows the median (central white line), interquartile range (black box bounds), and 1.5× the interquartile range (whiskers). (n = 1 for each off-target site) **c** Venn diagrams comparing off-target sites detected (or predicted) by Tracking-seq2, GUIDE-seq-2, and COSMID for Cas9 + sgRNA VEGFA2 or Cas9 + sgRNA HEK4. **d** Violin plot showing the distribution of Δindel rates measured by hybrid capture targeted sequencing at off-target sites belonging to different sets for Cas9 + VEGFA2 and Cas9 + HEK4. GUIDE-seq-2 and COSMID missed a considerable number of off-target sites detected by Tracking-seq2. Each inner box plot shows the median (central white line), interquartile range (black box bounds), and 1.5× the interquartile range (whiskers). (n = 1 for each off-target site) **e** Sequences, Track scores and Δindel rates of successfully sequenced Tracking-seq2-only off-target sites with indel frequencies ≥ 0.01% for Cas9 + sgRNA VEGFA2. Most of these sites contained non-canonical PAMs (non-NGG), and three sites contained 7 mismatches. Source data are provided as a Source Data file.

We compared Tracking-seq2 with the cell-free method CIRCLE-seq^8^ and the in-silico prediction tool Cas-OFFinder^24^ (Fig. 2a). To validate the off-target effects, we performed targeted hybrid capture sequencing, including probes targeting all off-target sites identified by CIRCLE-seq, Cas-OFFinder, and Tracking-seq2 for sgRNA VEGFA2 and HEK4, among which 5099 sites were successfully sequenced. The results revealed that most off-target sites detected solely by Tracking-seq2, or shared with CIRCLE-seq or Cas-OFFinder, exhibited higher indel rates (Fig. 2b). In contrast, most sites identified exclusively by CIRCLE-seq, Cas-OFFinder, or both, lacked detectable indels (Fig. 2b). These results indicate that although cell-free and computational methods are often considered highly sensitive with high false-positive rates, they may still miss true off-target sites, as many verified off-target sites were detected only by Tracking-seq2 (Fig. 2a, b).

We next compared Tracking-seq2 with a state-of-the-art cell-based method, GUIDE-seq-2^25^, and another computational method COSMID^26^ in HEK293T cells. Tracking-seq2 identified a larger number of off-target sites than GUIDE-seq-2 for both the HEK4 and VEGFA2 sgRNAs, capturing the vast majority of sites detected by GUIDE-seq-2 (Fig. 2c). The results from hybrid capture sequencing and targeted amplicon sequencing showed that Tracking-seq2 successfully detected numerous bona fide off-target sites that were missed by GUIDE-seq-2 or COSMID (Fig. 2d). Besides, GUIDE-seq-2 and COSMID also identified a few true off-target sites that were not captured by Tracking-seq2 (Fig. 2d).

For off-target sites uniquely identified by Tracking-seq2 and missed by all other methods, we found that most contained non-canonical PAMs (non-NGG), and three contained 7 mismatches (Fig. 2e). These two features made such off-target sites challenging for computational algorithms to predict and easy to miss with other methods. To further validate these atypical events, we performed targeted amplicon sequencing. Within our dataset, we identified five specific non-canonical PAMs (NAG, NCG, NTG, NGA, and NGC) and selected two representative off-target sites for each type. Both the targeted amplicon sequencing results (Supplementary Fig. 5a) and the distinct Tracking-seq2 signals (Supplementary Fig. 5b) confirmed bona fide off-target editing at all five non-canonical PAM types. Similarly, we performed targeted amplicon sequencing on the 3 off-target sites with 7 mismatches, as well as 7 sites with 6 mismatches. Most of them were verified as true off-target events (Supplementary Fig. 5c, d). While such events are rare, their occurrence is supported by previous literature^27–29^. Taken together, these data demonstrate that off-target editing can occur at sites with up to 7 mismatches and non-canonical PAMs, indicating the unique capability of Tracking-seq2 to uncover atypical off-target sites.

### Exogenous T5 can enhance the off-target detection sensitivity of BEs and PEs

**Fig. 3:**
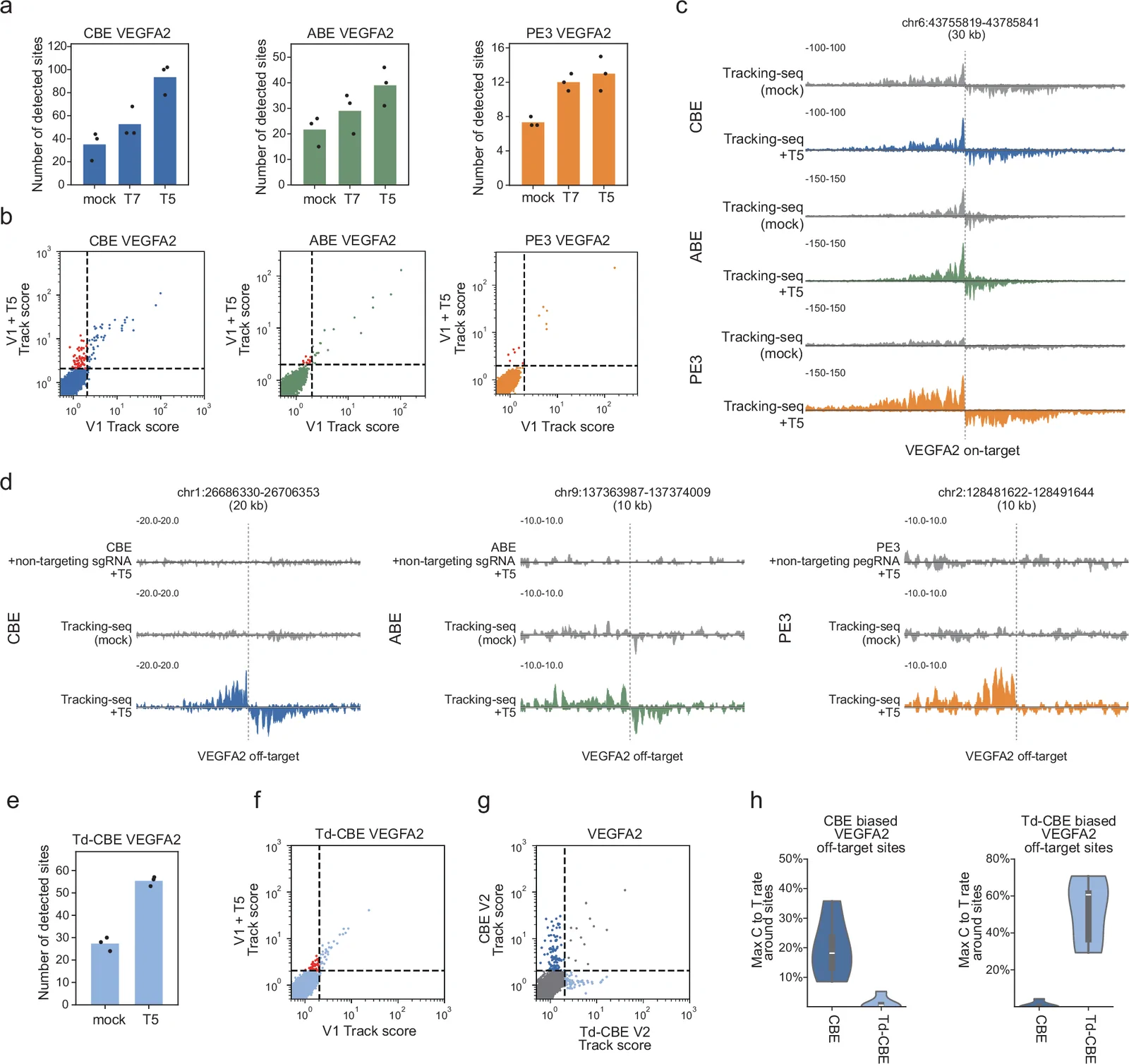
T5 enhanced the sensitivity of Tracking-seq for CBE, ABE, and PE in HEK293T cells. **a** Numbers of off-target sites caused by CBE, ABE, or PE3 using sgRNA/pegRNA VEGFA2 detected by Tracking-seq co-transfected with plasmids encoding T5, T7, or mock. Each dot represents a biological replicate (n = 3). **b** Scatter plot showing the Track scores of Tracking-seq with T5 co-transfection versus those without co-transfection, edited by CBE, ABE, or PE3 with sgRNA/pegRNA VEGFA2. Each dot represents a candidate off-target site. Dashed lines represent the thresholds of the Track score > 2 for positive sites. **c, d** Signals generated by Tracking-seq at the on-target site (c) and representative off-target sites (d) of sgRNA/pegRNA VEGFA2. The range in the top left of each track denotes normalized signal intensity, represented as read coverage per 10 million mapped reads. **e** Numbers of off-target sites caused by Td-CBEmax (a TadA-derived CBE variant) + sgRNA VEGFA2 detected by Tracking-seq in the mock group and the T5 group. Each dot represents a biological replicate (n = 3). **f** Scatter plot showing the Track scores of Tracking-seq with T5 co-transfection treatment versus those without treatment edited by Td-CBEmax + sgRNA VEGFA2. Each dot represents a candidate off-target site. Dashed lines represent the thresholds of the Track score > 2 for positive sites. **g** Scatter plots comparing the off-target sites detected for CBE4max and Td-CBEmax in HEK293T cells using sgRNA VEGFA2. **h** Violin plots showing the maximum C-to-T editing efficiency of 5 tested CBE4max-biased off-target sites (left) and 5 tested Td-CBEmax-biased off-target sites (right) in HEK293T cells (sgRNA VEGFA2). Each inner box plot shows the median (central white line), interquartile range (black box bounds), and 1.5× the interquartile range (whiskers). (n = 3 for each off-target site, mean values were used for the plot). Source data are provided as a Source Data file.

While BEs and PEs primarily generate NHEJ-independent SSBs, exogenous exonucleases hold promise for extending ssDNA at nicked sites to amplify Tracking-seq signals (Supplementary Fig. 1b). To test this, we first performed Tracking-seq for CBE (BE4max^30^), ABE (ABEmax^30^), and PE3^22^ in HEK293T cells, evaluating T5- and T7-exonuclease-treated samples alongside a mock control. Notably, Tracking-seq with T5 treatment amplified signals and identified novel off-target sites across all three editors (Fig. 3a–d and Supplementary Fig. 6a–f). This enhancement was most pronounced for CBE, detecting a ~ 3-fold increase in off-target sites for the sgRNA VEGFA2 (Fig. 3a).

To validate these novel off-target sites identified by Tracking-seq2, we performed targeted amplicon sequencing on the top five novel sites for each condition (CBE-VEGFA2, CBE-HEK4, ABE-VEGFA2, ABE-HEK4, PE3-VEGFA2, and PE3-HEK4). For CBE and ABE, all tested sites exhibited C to T or A to G editing, if there were C or A within the editing window (Supplementary Fig. 7a–d). For 4 sites of ABE lacking an A in the editing window, low-frequency indels were detected in 3 of them (Supplementary Fig. 7b). For PE3, the majority exhibited no significant editing (Supplementary Fig. 7e). Given that only three novel off-target sites were identified for PE3-HEK4, we additionally validated four sites jointly detected by both Tracking-seq V1 and V2. The results revealed that only the site with the strongest Tracking-seq2 signal (V1&V2-01) contained substantial off-target editing (Supplementary Fig. 7e). Considering the presence of distinct Tracking-seq2 signals at mutation-negative sites (Supplementary Fig. 7f), we hypothesize that while DNA nicking events did occur at these loci, they are efficiently repaired in an error-free manner. Our result is consistent with prior reports^22,31^ that PE exhibited high fidelity with much lower off-target effects.

As TadA-derived CBE variants are increasingly utilized, we investigated Td-CBEmax (Td-CBE)^32^ in our analysis with sgRNAs VEGFA2 or HEK4 in HEK293T cells. Tracking-seq with T5 treatment amplified signals and identified novel off-target sites (Fig. 3e, f and Supplementary Fig. 8a, b). Besides, we found that while Td-CBE generated fewer off-target sites than BE4max, the two editors exhibited mutually exclusive subsets of off-target sites (Fig. 3g and Supplementary Fig. 8c). We performed targeted amplicon sequencing on the top five differential off-target sites for each editor. The results verified these as genuine differential editing events (Fig. 3h and Supplementary Fig. 8d-f), demonstrating that Tracking-seq2 can reliably capture editor-dependent off-target profiles.

Collectively, these results demonstrate that T5 exonuclease enhances Tracking-seq sensitivity regardless of whether the genome editor relies on DSBs or SSBs. This broad applicability underscores the value of T5 treatment for improving off-target detection across diverse CRISPR-Cas systems. We therefore designate this T5-integrated approach for SSB-dependent editors as Tracking-seq2.

### Tracking-seq2 can be applied to human primary HSPCs and T cells

**Fig. 4:**
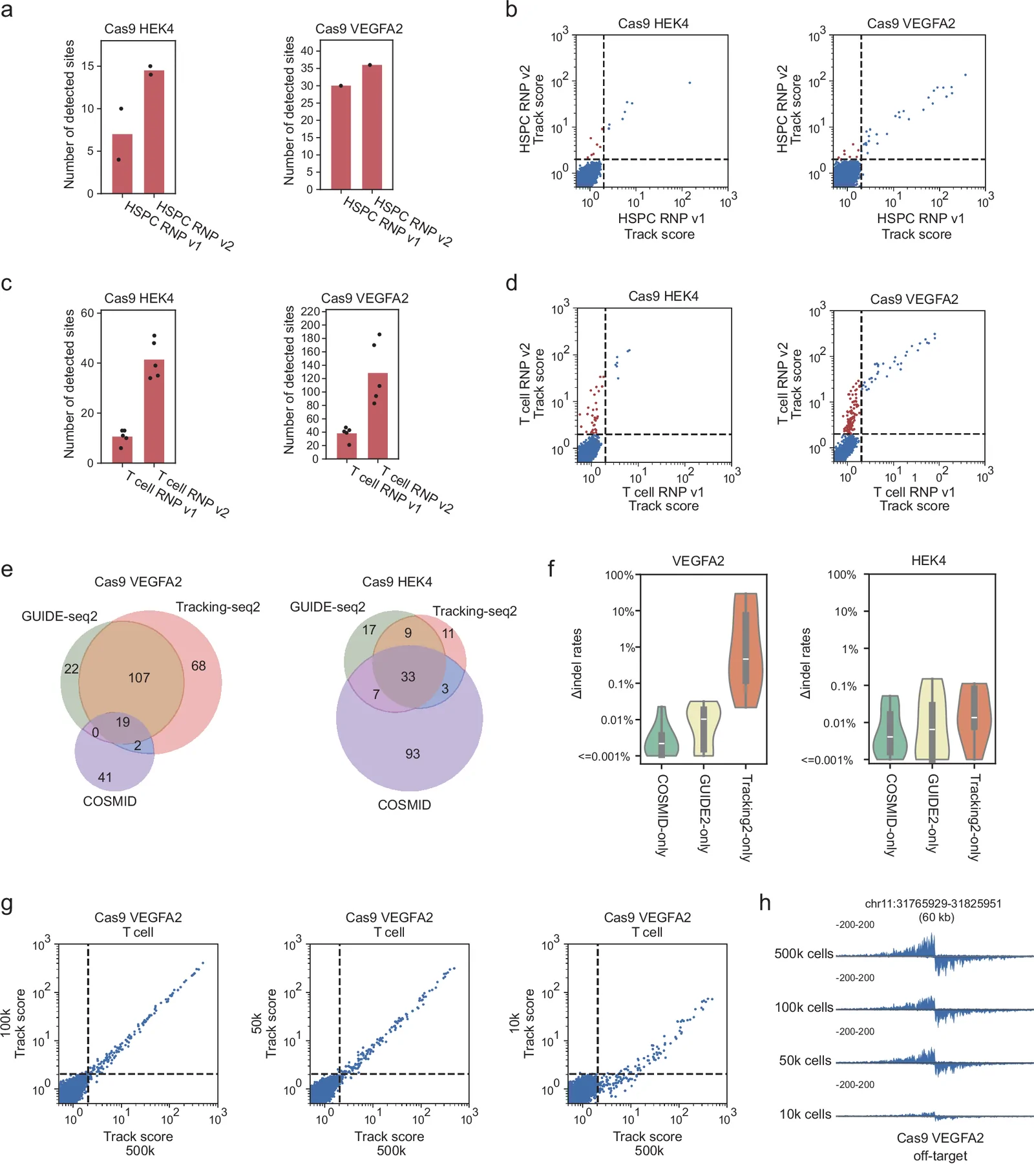
Tracking-seq2 enhances off-target detection sensitivity in human primary T cells and HSPCs. **a** Numbers of off-target sites detected by Tracking-seq (V1) and Tracking-seq2 (V2, i.e., V1 + T5 + KU-60648) in human HSPCs derived from cord blood (CB) edited by Cas9 RNP electroporation. Each dot represents an independent biological replicate (n = 2 for HEK4 and n = 1 for VEGFA2). **b** Scatter plot showing the Track scores of Tracking-seq2 versus Tracking-seq in human HSPCs. Each dot represents a candidate off-target site. Dashed lines represent the thresholds of the Track score > 2 for positive sites. Red dots indicate additional off-target sites detected by Tracking-seq2. **c** Numbers of off-target sites detected by Tracking-seq (V1) and Tracking-seq2 (V2) in human T cells edited by Cas9 RNP electroporation. Each dot represents a biological replicate (n = 5). **d** Scatter plot showing the Track scores of Tracking-seq2 versus Tracking-seq in human T cells. **e** Venn diagrams comparing off-target sites detected (or predicted) by Tracking-seq2, GUIDE-seq-2, and COSMID for Cas9 + VEGFA2 or Cas9 + HEK4 in human T cells. **f** Violin plot showing the distribution of Δindel rates measured by targeted amplicon sequencing at method-specific off-target sites (top 10 per method) for Cas9 + VEGFA2 or Cas9 + HEK4 in human T cells. Off-target sites exclusively identified by Tracking-seq2 exhibited the highest indel frequencies compared to those uniquely detected by GUIDE-seq-2 or COSMID. Each inner box plot shows the median (central white line), interquartile range (black box bounds), and 1.5× the interquartile range (whiskers). (n = 3 for each off-target site, mean values were used for the plot) **g** Correlation between Track scores in experiments with varying human T cell inputs (10,000, 50,000, 100,000, and 500,000 cells). Each dot represents a candidate off-target site. Sensitivity with 100,000 or 50,000 cells was only slightly lower than that with 500,000 cells, while assays using 10,000 cells missed off-target sites with low signals. **h** Tracking-seq2 signals at a representative off-target site using different numbers of human T cells. Source data are provided as a Source Data file.

To assess its clinical utility, we applied Tracking-seq2 to two clinically relevant primary human cell types edited via Cas9 ribonucleoprotein (RNP) electroporation: T cells and hematopoietic stem and progenitor cells (HSPCs). For both cell types, Tracking-seq2 successfully enhanced off-target detection over the original Tracking-seq (Fig. 4a–d). Specifically, in HSPCs, it yielded a ~ 2-fold and ~1.2-fold increase for HEK4 and VEGFA2, respectively (Fig. 4a, b); in T cells, Tracking-seq2 enabled a ~ 4-fold increase in detected off-target sites for both HEK4 and VEGFA2 (Fig. 4c, d).

To evaluate Tracking-seq2 against current state-of-the-art methods, we performed direct benchmarking comparisons with GUIDE-seq-2 and COSMID in primary T cells. The three methods shared common off-target sites and also included unique sites (Fig. 4e). To validate these unique sites, we performed targeted amplicon sequencing on the top-10 unique sites specific to each method. The results showed that Tracking-seq2-only sites had higher indel rates than the others, while the other two methods also identified several genuine editing sites (Fig. 4f).

To validate the robustness of Tracking-seq2 with low cell inputs, we titrated starting cell numbers (10,000, 50,000, 100,000, and 500,000 cells) in both HEK293T and primary T cells for the experiments. Compared to the 500,000-cell baseline, Tracking-seq2 with 50,000 or 100,000 cells resulted in only a slight reduction in detected off-target sites for both cell types (Fig. 4g, h and Supplementary Fig. 9). However, with an input of 10,000 cells, weak off-target signals became indistinguishable from background noise. Therefore, we recommend ≥50,000 cells for primary cells to balance sensitivity with sample availability.

### Genomic variations drive distinct off-target heterogeneity across cell types and individuals

**Fig. 5:**
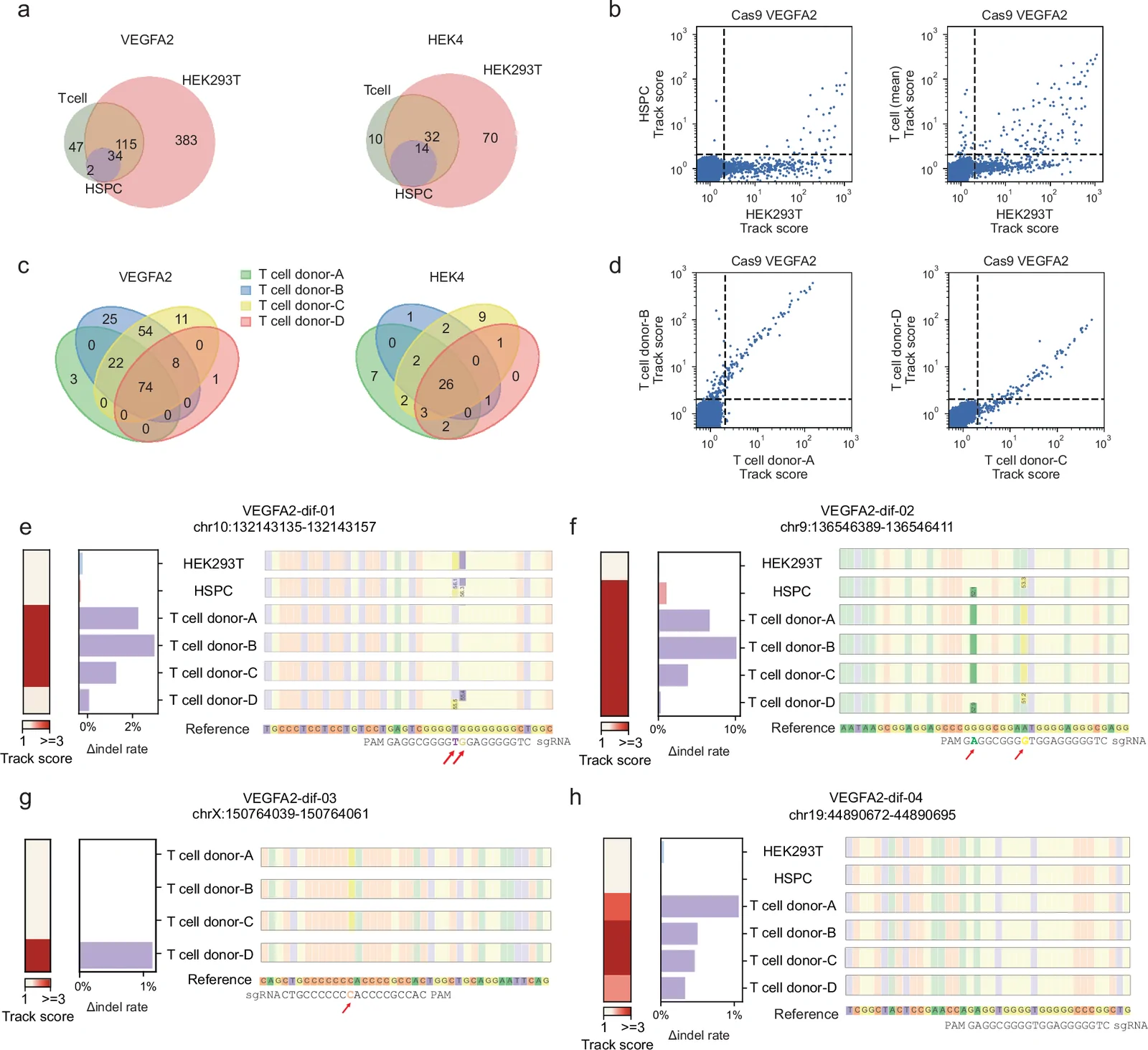
Genomic variations drive off-target heterogeneity across cell types and individuals. **a** Venn diagram comparing off-target sites generated by Cas9 with sgRNA VEGFA2 (left) and sgRNA HEK4 (right) in HEK293T cells, HSPCs, and T cells. HSPCs and T cells were derived from different donors. **b** Scatter plot comparing the Track scores in HEK293T cells with HSPCs and T cells for the sgRNA VEGFA2. **c** Venn diagram comparing off-target sites generated by Cas9 with sgRNA VEGFA2 (left) and sgRNA HEK4 (right) in primary human T cells from four different donors (Donors A, B, C, and D). **d** Scatter plot comparing Track scores across different donors for the sgRNA VEGFA2. **e–h** Genomic sequences of unedited HEK293T cells, HSPCs, and T cells from four different donors at the identified differential off-target sites for the sgRNA VEGFA2, as determined by targeted amplicon sequencing. For the VEGFA2-dif-01 locus (**e**), T cells from donors A, B, and C harbored a genomic sequence identical to the hg38 reference, which matched the sgRNA at the marked two nucleotides. In contrast, T cells from donor D and the HSPCs were heterozygous at these two nucleotides, while HEK293T cells were homozygous for the variant, both resulting in mismatches with the sgRNA sequence. For VEGFA2-dif-02 (**f**), T cells from donors A, B, and C contained two homozygous nucleotide polymorphisms that matched the sgRNA sequence, whereas HEK293T cells were homozygous at these two nucleotides that mismatched the sgRNA sequence. HSPCs and T cells from donor D were heterozygous at these positions, resulting in weak off-target editing. For VEGFA2-dif-03 (**g**), T cells from donors A, B, and C contained a homozygous nucleotide polymorphism that mismatched the sgRNA sequence, while T cells from donor D matched the nucleotide. For VEGFA2-dif-04 (**h**), the genomic sequences across all samples were homozygous and completely identical, indicating that genomic variations cannot account for all the observed off-target heterogeneity, and other factors may contribute. Source data are provided as a Source Data file.

Notably, off-target profiles for identical sgRNAs exhibited heterogeneity across HEK293T cells, human T cells, and HSPCs (Fig. 5a, b and Supplementary Fig. 10a). Although most off-target sites in T cells and HSPCs overlapped with those in HEK293T cells, a few sites were exclusively detected in T cells or HSPCs. Furthermore, we observed differential off-target effects among T cells from different donors (Fig. 5c, d and Supplementary Fig. 10b). Track scores showed a robust global correlation across T cells from different donors, while specific sites emerged as clear outliers from the global trendline, suggesting donor-specific heterogeneity in off-target effects at these sites (Fig. 5d and Supplementary Fig. 10b).

We conducted targeted amplicon sequencing and confirmed that differential off-target editing existed across cell types and T cell donors (Fig. 5e–h and Supplementary Fig. 10c, d). By analyzing the genomic sequences of the unedited cells, we discovered that inherent genomic variations at specific off-target sites were largely responsible for the heterogeneity of certain sites (Fig. 5e-g): nucleotide polymorphisms increased local sequence homology to the sgRNA, thereby driving higher off-target activity in particular cells.

Conversely, we also identified differentially edited off-target sites that shared identical genomic sequences (Fig. 5h and Supplementary Fig. 10c, d), indicating additional factors contributing to the heterogeneity. We hypothesized that cell-type-specific epigenetic states might influence off-target effects. To explore this, we analyzed DNase-seq, H3K4me3 ChIP-seq, and H3K27ac ChIP-seq data from HEK293T cells, HSPCs, and T cells (Supplementary Fig. 10e). While we observed minor variations in epigenetic signals, these did not exhibit a clear correlation with the distinct off-target profiles.

Taken together, these data support that individual-specific genetic backgrounds influence off-target profiles, emphasizing the clinical risk of relying on off-target data from reference cell lines or unmatched donors for patient safety assessments.

## Discussion

Accelerating clinical translation of CRISPR therapies necessitates highly sensitive, direct off-target profiling in primary human cells. We developed Tracking-seq2 through editor-specific biochemical optimizations, achieving high sensitivity directly in human primary cells and uncovering individual-specific off-target effects due to inter-individual genetic diversity.

Tracking-seq2 employs a two-step workflow to balance detection sensitivity with physiological relevance. The first “detection phase” utilizes T5 exonuclease and NHEJ inhibitors to perturb the repair landscape and amplify RPA-ssDNA signals, thereby maximizing the discovery of low-frequency off-target sites. Although the treatment may induce a certain degree of cytotoxicity, the treated cells are used exclusively for Tracking-seq2 and are not intended for practical editing scenarios. As this treatment amplifies RPA-ssDNA intermediates and may overestimate rare off-target events, the second “validation phase” leverages targeted amplicon or hybrid capture sequencing under standard physiological conditions (without T5 or KU-60648) to accurately measure true off-target editing efficiencies. While signal amplification during the detection phase alters the native repair environment and may cause additional DNA damage, our validation phase confirmed that a substantial proportion of the newly identified candidates were genuine off-target events under physiological conditions. Although this two-step paradigm increases experimental complexity, it is also required by other state-of-the-art methods (e.g., GUIDE-seq^12^/GUIDE-seq-2^25^, DISCOVER-seq+^14^). More importantly, this trade-off yields markedly enhanced sensitivity over the original Tracking-seq, fully justifying the additional effort.

Compared to existing methods, Tracking-seq2 offers distinct technical advantages. First of all, most off-target detection technologies require DSBs and are thus mainly confined to the Cas9 system (e.g. GUIDE-seq^12^/GUIDE-seq-2^25^, DISCOVER-seq^13^/DISCOVER-seq^+14^, and CIRCLE-seq^8^), and other editors require dedicated methods (e.g. Detect-seq^16^ for CBEs). Besides, GUIDE-seq/GUIDE-seq-2 requires the integration of exogenous oligodeoxynucleotide at off-target sites, which may trigger apoptosis due to high levels of free DNA ends in fragile primary cells^33^. Furthermore, while DISCOVER-seq^13^/DISCOVER-seq^+14^ requires 1-5 million input cells, Tracking-seq2 operates with substantially lower cell inputs (as few as 50,000 cells)—a critical asset for scarce clinical samples. Unlike cell-free biochemical assays (e.g., CIRCLE-seq^8^, SITE-seq^10^) and in silico tools (e.g., Cas-OFFinder^24^ and COSMID^26^), Tracking-seq2 functions within native chromatin contexts, thereby eliminating the vast number of false positives typically associated with naked DNA templates or simple sequence alignment. Our direct benchmarking against GUIDE-seq-2 and COSMID demonstrated that Tracking-seq2 achieves comparable, if not superior, sensitivity in primary T cells. Importantly, each method identified unique bona fide off-target sites missed by the others. This underscores that these technologies are complementary and that their combined use offers the most rigorous strategy for comprehensive clinical safety assessment.

Despite these strengths, several limitations of the current study warrant consideration. First, while Tracking-seq2 effectively identifies off-target cleavage or nicking events, it does not provide information about structural variations, such as large deletions or translocations. Second, the sensitized condition of T5/KU-60648 treatment may overestimate certain weak off-target cleavage/nicking events, which may not lead to detectable off-target editing at such sites under physiological conditions. Third, because the assay captures transient RPA-ssDNA intermediates, it is inherently constrained to specific temporal windows of editing and may overlook late-occurring off-target events beyond early sampling timepoints. Fourth, for CBEs, detection sensitivity remains suboptimal for certain weak off-target effects. The SSB repair cascades are currently poorly characterized and lack potent small-molecule inhibitors, hindering further signal amplification for base editors. We recommend Detect-seq as a complementary cell-based method tailored for CBEs. Finally, our study is restricted to in vitro and ex vivo systems, and the performance of Tracking-seq2 for in vivo off-target profiling deserves further investigation.

Despite these limitations, our findings strongly emphasize that inter-individual genetic diversity shapes off-target profiles, precluding the reliance on reference cell lines or unmatched donors for rigorous patient safety assessments. By enabling direct, low-input profiling in patient-derived cells, Tracking-seq2 serves as a powerful technology for evaluating potential patient-specific risks, which is essential for the safe clinical translation of genome-editing applications.

## Methods

### Ethics statement

This research complies with all relevant ethical regulations, approved by the East China Normal University Committee on Medical Ethics, and informed consent was obtained from all human participants prior to sample collection.

### Cell culture

HEK293T cells (ATCC, CRL-3216) were cultured in DMEM (Gibco, C11995500BT) supplemented with 10% FBS (HUANKE, HK-W500) and 1% penicillin–streptomycin (HyClone, SV30010) at 37 °C with 5% CO₂.

HSPCs used in this study were derived from cord blood (purchased from Milestone Biological Science and Technology). HSPCs were cultured in Serum-Free Expansion Medium (STEMCELL, 09650) supplemented with 100 ng/mL Recombinant Human SCF (Peprotech, 300-18), 100 ng/mL Recombinant Human TPO (Peprotech, 300-07), and 100 ng/mL Recombinant Human Flt3-L (Peprotech, 300-19).

Human T cells from healthy donors were obtained from Zhejiang Provincial People’s Hospital (Hangzhou, China) with informed consent and institutional ethics approval. Recruitment of healthy donors was conducted under the approval of the Clinical Research Ethics Committee of Zhejiang Provincial People’s Hospital. T cells were stimulated using T cell TransAct (Miltenyi Biotec, 130-111-160) and cultured in X-VIVO 15 medium (Lonza, 04-418Q) supplemented with 2% human AB serum (Sigma-Aldrich, H4522) or CTS Immune Cell Serum Replacement (Thermo Fisher Scientific, A4702902), along with recombinant human IL-2 (100 U/mL), IL-7 (5 ng/mL), and IL-15 (5 ng/mL).

### Plasmids

The pSpCas9(BB)-2A-GFP (PX458) plasmid (Addgene, #48138) was utilized for all Cas9-based editing experiments. Expression plasmids for AsCas12a and HiFi were constructed by replacing the Cas9 gene in the PX458 vector with their corresponding coding sequences (Addgene #69982 and #140563, respectively). For base editing, cytosine base editor (CBE) and adenine base editor (ABE) were expressed from editor-P2A-mCherry plasmids, and the coding sequences of CBE and ABE were derived from pCMV-AncBE4max (Addgene #112094) and pCMV-ABEmax-P2A-GFP (Addgene #112101). For PE3 editing, the DNA sequence encoding for MMLV was synthesized by GENEWIZ, and PEmax-P2A-mCherry was cloned using Gibson assembly by combining DNA fragments encoding the three components: nCas9, MMLV, and mCherry. For Td-CBEmax editing, the Td-CBEmax (Addgene, #196600) plasmid was a gift from Dali Li’s Lab. All the exonuclease plasmids were a gift from Yinqing Li’s Lab. For sgRNA expression plasmid construction, complementary oligonucleotides encoding the target spacer were phosphorylated using T4 Polynucleotide Kinase (T4 PNK, Thermo Fisher Scientific, EK0032), annealed by gradual cooling from 95 °C to 25 °C, and ligated into a BbsI-digested U6-sgRNA-GFP vector using T4 DNA ligase. The sequences of all sgRNAs, pegRNAs, and dsODNs used in this study are listed in Supplementary Data 4.

### Cell transfection and nucleofection

For HEK293T cells, cells were seeded in six-well culture plates at a density of 1-2 × 10⁶ cells per well and incubated overnight at 37 °C with 5% CO₂. Transfection was performed when cells reached 60–80% confluency. For each well, 3 μg of plasmid DNA was mixed with 12 μg of PEI MAX (PolyScience, 24765) and incubated at room temperature for 15 min to form DNA-PEI complexes. The total amount and composition of plasmids varied depending on the editing system. For base editing (BE), 3 μg DNA per well was used at a ratio of BE expression plasmid: sgRNA plasmid: exonuclease-encoding plasmid = 1 μg: 0.5 μg: 1.5 μg. For prime editing 3 (PE3), 3 μg DNA per well was used, consisting of prime editor expression plasmid: pegRNA plasmid: nicking sgRNA plasmid: exonuclease-encoding plasmid = 0.75 μg: 0.375 μg: 0.375 μg: 1.5 μg. For Cas9-mediated editing, 3 μg DNA per well was used at a ratio of Cas9 expression plasmid: exonuclease-encoding plasmid = 1.5 μg: 1.5 μg. To expose cells to DNA repair inhibitors, they were added to the culture media before transfection at a final concentration of 1.25 μM KU-60648 (Yeasen, 53429ES08), 25 μM DIDS sodium salt (MCE, HY-D0086), 7.5 μM RI-1 (MCE, HY-15317), 10 μM IBR2 (MCE, HY-103710), 0.25 μM NU7441 (MCE, HY-11006), 0.5 μM STL127705 (MCE, HY-122727), 0.25 μM Olaparib (MCE, HY-10162). All drug stock solutions were diluted in dimethyl sulfoxide (DMSO). At 8-12 h post-transfection, the medium containing transfection complexes was replaced with fresh, pre-warmed drug-supplemented medium. Cells were then maintained in culture until collection at designated timepoints (48 h post-transfection for Cas9, Cas12a, and PE3; 24 h post-transfection for CBE and ABE).

For nucleofection of HSPCs, 1 × 10⁶ cells were nucleofected with RNP complexes containing 90 μg of Cas9 protein and 60 μg of sgRNA. For human primary T cells, nucleofection was performed at a density of 5 × 10⁶ cells with RNP complexes comprising 30 μg of Cas9 protein and 30 μg of sgRNA.

All nucleofection procedures were performed according to the manufacturer’s instructions using the Lonza 4D-Nucleofector System. Briefly, HSPCs (1 × 10⁶ per reaction) or primary T cells (5 × 10^6^ cells per reaction) were collected by centrifugation, washed once with PBS, and resuspended in 100 μL of P3 buffer. For each reaction, ribonucleoprotein (RNP) complexes were pre-assembled by incubating Cas9 protein with sgRNA at room temperature for 10-15 min prior to nucleofection. For T5 exonuclease-assisted editing, T5 Exonuclease (New England Biolabs, M0663L; 10,000 U/mL) was added directly to the pre-assembled RNP complex immediately before mixing with cells. Specifically, for 1 × 10⁶ cells, 0.5 μL of T5 Exonuclease was used per reaction (final amount: 5 U), and the volume was scaled proportionally with increasing cell numbers if needed. The RNP ( ± T5 Exonuclease) mixture was then gently combined with the cell suspension and transferred into cuvettes, avoiding bubble formation.

Nucleofection was carried out using program DZ-100 for HSPCs or EO115 for T cells. Immediately after nucleofection, 500 μL of pre-warmed culture medium was gently added to each cuvette to facilitate cell recovery, and cells were carefully resuspended and transferred into six-well plates containing pre-equilibrated culture medium. Cells were subsequently incubated at 37 °C in a humidified atmosphere with 5% CO₂. To transiently inhibit DNA repair pathways, KU-60648 was added to the culture medium at a final concentration of 1 μM immediately after nucleofection and maintained for the indicated duration.

### Tracking-seq and Tracking-seq2

Transfected HEK293T cells expressing EGFP (for Cas9 and Cas12a) or both EGFP and mCherry (for BE and PE) were sorted using a BD FACSAria Fusion Flow Cytometer. For CD34⁺ HSPCs or primary T cells, viable cells were identified and sorted by DAPI (Invitrogen, D1306) exclusion. To evaluate the assay’s sensitivity with low-input samples, additional Tracking-seq2 experiments were conducted using serially titrated cell numbers (10,000, 50,000, 100,000, and 500,000 cells). For other experiments, 500,000 cells were used for the following steps.

Cells were centrifuged at 500 g for 5 min, and resuspended in 50 μL of wash buffer (20 mM HEPES, pH 7.5, 150 mM NaCl, 0.5 mM spermidine, 1 mg/mL Roche Complete Protease Inhibitor). Subsequently, 10 μL of Concanavalin A (ConA) beads suspension (Polysciences, 86057-3) was added. After gentle vortexing, the mixture was incubated at room temperature for 10 min, placed on a magnetic stand to remove the supernatant, and the beads were resuspended in 100 μL of antibody buffer (20 mM HEPES, pH 7.5, 150 mM NaCl, 0.5 mM spermidine, 1.2 mg/mL Roche Complete Protease Inhibitor, 0.05% digitonin, 2 mM EDTA) containing 1 μg of RPA2 primary antibody (Millipore, MABE285). The mixture was incubated on a tube rotator at room temperature for approximately 2 h to allow antibody binding, followed by washes twice with 100 μL of digitonin buffer (20 mM HEPES, pH 7.5, 150 mM NaCl, 0.5 mM spermidine, 1.2 mg/mL Roche Complete Protease Inhibitor, 0.05% digitonin). Subsequently, the beads were resuspended in 80 μL of digitonin buffer containing 56 ng pA/MNase and incubated at 4 °C for 1 h on a tube rotator. After incubation, the supernatant was removed, and the beads were washed twice with 100 μL of digitonin buffer. The beads were resuspended in 100 μL of digitonin buffer and equilibrated on ice for 2 min, followed by the addition of 2 μL of pre-cooled 100 mM CaCl₂ and incubation on ice for 30 min to initiate pA/MNase cleavage. The reaction was halted by adding 100 μL of 2 × stop buffer (0.34 M NaCl, 0.02 mM EDTA, 0.004 mM EGTA, 0.02% digitonin, 0.1 mg/mL glycogen, 0.05 mg/mL RNase A) and incubating on a tube rotator for 30 min at 37 °C to release DNA fragments. The sample was then centrifuged at 16,000 × *g* for 5 min at 4 °C, and the supernatant was collected into a 0.2 mL PCR tube using a magnet stand. Next, add 2 μL 10% SDS (Solarbio, S1010-100) and 2 μL Proteinase K (Roche, 3115887001) to the tube, followed by incubating at 56 °C for 45 min and 72 °C for 20 min. DNA was purified using 3 × VAHTS DNA Clean Beads (Vazyme, N411) and eluted in 20 μL of ultra-pure water. To eliminate background dsDNA, the eluted chromatin was treated with HL-dsDNase (Novoprotein, M127-01A) by adding 1 μL of HL-dsDNase enzyme and 2 μL of 10 × buffer, followed by incubation at 37 °C for 1 h and enzyme inactivation at 75 °C for 20 min.

Strand-specific ssDNA libraries were constructed using the VAHTS ssDNA LibraryPrep Kit for Illumina (Vazyme, ND620), according to the manufacturer’s instructions. Briefly, DNA samples were initially denatured at 95 °C for 2 min and immediately chilled on ice for 2 min. Adaptase-based adapters were ligated to the ssDNA by adding 10 μL of 3′ ligation buffer, 5 μL of 3′ ligation enzyme mix, and 5 μL of 3′ adapter, followed by incubation at 75 °C for 15 min and heat inactivation at 95 °C for 2 min. For DNA extension, 5 μL of extension primer and 35 μL of extension enzyme mix were added, and the reaction was carried out at 98 °C for 30 s, 63 °C for 15 s, and 68 °C for 5 min. The product was purified using 1.8 × VAHTS DNA Clean Beads. Subsequently, 10 μL of 5′ ligation mix and 10 μL of 5′ adapter were added and incubated at 25 °C for 15 min to facilitate adapter ligation, followed by a second purification using 1.6 × VAHTS DNA Clean Beads. Finally, the library was amplified using VAHTS HiFi Amplification Mix and sequenced on the DNBSEQ-T7 platform.

### Analysis of tracking-seq/tracking-seq2 data

The sequencing data of Tracking-seq/Tracking-seq2 were analyzed using Offtracker with default parameters. The software and a detailed pipeline are available at GitHub (https://github.com/Lan-lab/offtracker). Offtracker is an end-to-end software tailored for Tracking-seq/Tracking-seq2 data. In brief, fastq files were mapped to the hg38 reference genome with chromap^34^ in a strand-specific manner with “-l 3000 --low-mem --BED --remove-pcr-duplicates --trim-adapters --min-read-length 10 --allocate-multi-mappings” parameters. All candidate off-target sites of a given sgRNA were enumerated by blastn-short^35^ with word size = 4. Offtracker calculated Track scores for each candidate site according to the normalized read counts mapped to the forward strand and the reverse strand around the site. Offtracker deducted normalized signals in the control group from those in the experimental group to distinguish signals caused by the specific genome editor + gRNA from any source of background noise. The Track score is a normalized score reflecting signal enrichment and strand specificity for each site with an empirical threshold > 2 for calling positive off-target sites. More detailed, step-by-step codes and instructions can be found in our Nature Protocols^36^ and our GitHub page for the latest update. For replicates in the same condition, the mean Track scores were calculated for scatter plots and subsequent analyses. The exact Track scores of scatter plots can be found in Supplementary Data 1.

### GUIDE-seq-2

For HEK293T cells, cultures were maintained to approximately 80% confluency prior to nucleofection. Then, 1 × 10^6^ HEK293T cells were nucleofected with 2.25 µg of plasmid DNA (expressing both SpCas9 and sgRNA) and 25 pmol of the GUIDE-seq double-stranded oligodeoxynucleotides (dsODN; oSQT685/686) in 100 µL of P3 buffer using Lonza Nucleofector 4-D system (program CM-130) according to the manufacturer’s protocol. For human T cells from donor-B/C/D, nucleofection (program EO115) was performed with 5 × 10^6^ cells using RNP complexes containing 30 μg each of Cas9 protein and sgRNA, along with 300 pmol of dsODN, in 100 µL of P3 buffer. Immediately after nucleofection, cells were gently resuspended in 500 µL of pre-warmed medium per cuvette and transferred to six-well plates containing pre-equilibrated medium, followed by incubation at 37 °C in a humidified 5% CO₂ atmosphere. Genomic DNA was isolated approximately 72 h post-transfection using TIANamp Genomic DNA Kit (TIANGEN, DP304), following the manufacturer’s instructions. On-target dsODN integration was assessed by PCR amplification followed by Sanger sequencing.

GUIDE-seq-2 reactions were performed as described in the paper^25^. In brief, the Tn5 transposase (Robust, M0221) was prepared by combining 2 µL of Tn5 (1 U/µL), 25 pmol annealed i5 adapter oligos encoding 8-nt barcodes and 10-nt unique molecular indexes (UMIs), with 1 µL 10 × TPS buffer at 25 °C for 30 min. Then, 100 ng of gDNA was tagmented with 4 µL of the assembled Tn5 transposase, 6 µL 5 × LM buffer and 3 µL 50 mM MgCl_2_ at 55 °C for 7 min according to the manufacturer’s instructions. The reaction was halted using 30 µL of Stop buffer (1% SDS, 100 mM EDTA, and 0.4 µg/µL Proteinase K) and incubation at 55 °C for 30 min. The fragmented DNA was purified by 1.8 × (108 µL) VAHTS DNA Clean Beads and eluted in 11 µL of TE buffer. Separate PCR amplifications were carried out using dsODN sense- and antisense-specific primers with Platinum Taq DNA Polymerase (Thermo Fisher Scientific, 10966-018). The thermocycling conditions consisted of an initial denaturation at 95 °C for 5 min; 15 touchdown cycles of 95 °C for 30 s, 70 °C for 120 s (decreasing by 1 °C per cycle), and 72 °C for 30 s; 20 standard amplification cycles of 95 °C for 30 s, 55 °C for 60 s, and 72 °C for 30 s; and a final extension at 72 °C for 5 min. PCR products were purified with 1.8 × (54 µL) VAHTS DNA Clean Beads and eluted in 30 µL TE buffer. Sense and antisense libraries were quantified using a Qubit 4.0 Fluorometer and pooled in equal amounts. The analysis of GUIDE-seq-2 data was carried out using the pipeline provided by the authors on GitHub (https://github.com/tsailabSJ/guideseq) using hg38 as the reference genome.

### Targeted amplicon sequencing

Genomic DNA was extracted from FACS-sorted cells using QuickExtract DNA Extraction Solution (Lucigen, QE09050), following the manufacturer’s instructions. Cells were collected 120 h post-transfection at a ratio of approximately 2000 cells per microliter of QuickExtract solution to ensure sufficient genomic input. Amplicon PCR primers targeting potential off-target regions were designed using the Primer-BLAST tool provided by the National Center for Biotechnology Information (NCBI). To achieve specific amplification, a nested PCR strategy was employed. The first-round PCR was carried out using 2 × Phanta Max Master Mix (Vazyme, P515-03), with approximately 50 ng of genomic DNA used per off-target site. A total of 35 amplification cycles was performed. The second-round PCR was then conducted to introduce unique sequencing indices for sample multiplexing. The second round of PCR primers incorporated Illumina adapter sequences to ensure compatibility with next-generation sequencing (NGS) platforms. 10 bp UMIs were added to the second-round PCR primers. Following amplification, the PCR products were assessed by agarose gel electrophoresis to confirm quality and integrity. Target bands were excised and purified using the RaPure DNA Clean Up Kit (Magen, MD010). The purified amplicon libraries were quantified using a Qubit 4.0 Fluorometer and pooled for high-throughput sequencing. Sequencing was conducted on the Illumina NovaSeq 6000 platform. To correct sequencing errors or PCR errors, for reads with identical UMIs and mapping locations, only the most frequently observed sequences were kept. The targeted amplicon sequencing data were analyzed using CRISPResso2^37^. All displayed Δindel rates were the results of the experimental group minus the wild-type (unedited) group, clipped at zero or 0.00001 for log-scale plots. When evaluating the off-target editing for different editors: for Cas9, we regard off-target editing as indels within ±1 bp of the cleavage position (3 bp upstream the PAM); for ABE, we regard A-to-G conversion within the 20 bp sgRNA range and indel ±1 bp around the cleavage position as off-target editing; for CBE, we regard C-to-T conversion within the 20 bp sgRNA range and indel ±1 bp around the cleavage position as off-target editing; for PE, we regard all types of substitutions and indel ±10 bp around the 5′ of PAM as off-target editing. *P*-values for edited group versus unedited group were calculated by one-sided binomial tests and the exact values were listed in Supplementary Data 2 and Supplementary Data 3.

### Targeted hybrid capture sequencing

500,000 cells were collected 96 h after transfection. Genomic DNA of edited cells was extracted using TIANamp Genomic DNA Kit (TIANGEN, DP304) and then sonicated to 150-200 bp fragments. For each sample, 1 μg of fragmented DNA was used for the following steps. Then, IGT® Fast Library Prep Kit v2.0 (iGeneTech, C10022) was used to build whole-genome DNA libraries with UMIs. Biotinylated oligonucleotide probes for capturing the off-target sites were ordered from iGeneTech Bioscience Co., Ltd. and used in conjunction with TargetSeq One® Hyb & Wash Kit v2.0 (iGeneTech, C10331). The captured DNA was sequenced using DNBSEQ-T7. The adapters of fastq data were trimmed by fastp^38^ and then mapped to hg38 using bowtie2^39^. The bam files were sorted using sambamba^40^. To correct sequencing errors or PCR errors, for reads with identical UMIs and mapping locations, only the most frequently observed sequences were kept. The deduped BAM files were analyzed using CRISPRessoWGS^37^ to evaluate the editing result of each off-target site. All displayed Δindel rates were the results of the experimental group minus the wild-type (unedited) group, clipped at zero or 0.00001 for log-scale plots. Only Cas9-edited off-target sites involved the hybrid capture sequencing and the off-target editing was defined as indels within ±1 bp of the cleavage position (3 bp upstream the PAM).

### Statistics & reproducibility

No statistical method was used to predetermine sample size. The exact sample sizes are provided in the respective figure legends. In the targeted amplicon sequencing and hybrid-capture targeted sequencing data analysis, off-target sites with mapping rates below 10% or mapped reads below 1000 were considered failures and excluded. Allocation of samples was random. The investigators were not blinded to allocation during experiments and outcome assessment, as the inherently distinct protocols of different off-target detection methods preclude blinding during execution; however, all data were processed using pre-established pipelines. Statistical analyses were performed using statsmodels in Python, and the exact statistical tests and *P*-values are explicitly detailed in the respective figure legends and Supplementary Data files. Bar plots present the mean values with individual data points shown as dots. To robustly demonstrate the sensitivity of Tracking-seq2, experiments were performed independently across multiple conditions, yielding similar results across biological replicates, confirming the reproducibility of the study.

### Reporting summary

Further information on research design is available in the Nature Portfolio Reporting Summary linked to this article.

## Supplementary information

Supplementary Information

Description of Additional Supplementary Files

Supplementary Data 1

Supplementary Data 2

Supplementary Data 3

Supplementary Data 4

Reporting Summary

Transparent Peer Review file

## Source data

Source Data

## Data Availability

The data of HEK293T cells generated in this study have been deposited in NCBI GEO database under accession code GSE307225. The raw data and processed data of human primary cells have been deposited in GSA-Human database under accession code HRA013027 and OMIX database under accession code OMIX011731, respectively. For public epigenetic data, we downloaded the bigwig files from the ENCODE portal^41^ with the following identifiers: ENCFF148BGE, ENCFF416AAY, ENCFF719ZZK, ENCFF315TAU, ENCFF795ZQG, ENCFF384CAD, ENCFF157TGK, ENCFF537GCQ, and ENCFF314OSR, for DNase-seq, H3K4me3 ChIP-seq, and H3K27ac ChIP-seq of HEK293T cells, HSPCs and T cells. Off-target results and other data are available in the Supplementary Data files and the Source Data file. Source data are provided with this paper.

## Code availability

Codes and guides for analyzing Tracking-seq/Tracking-seq2 are available on GitHub^42^ (https://github.com/Lan-lab/offtracker).
