# Supplementary material for: Ultra-sensitive profiling of CRISPR-Cas off-target effects with Tracking-seq2
Source: Nat Commun. 2026 Aug 11;17:9659. doi: 10.1038/s41467-026-76778-9 (PMC13558771; doi:10.1038/s41467-026-76778-9)
Supplement: Supplementary file 1 [file 41467_2026_76778_MOESM1_ESM.pdf]

Supplementary information

Supplementary Figures 1-10

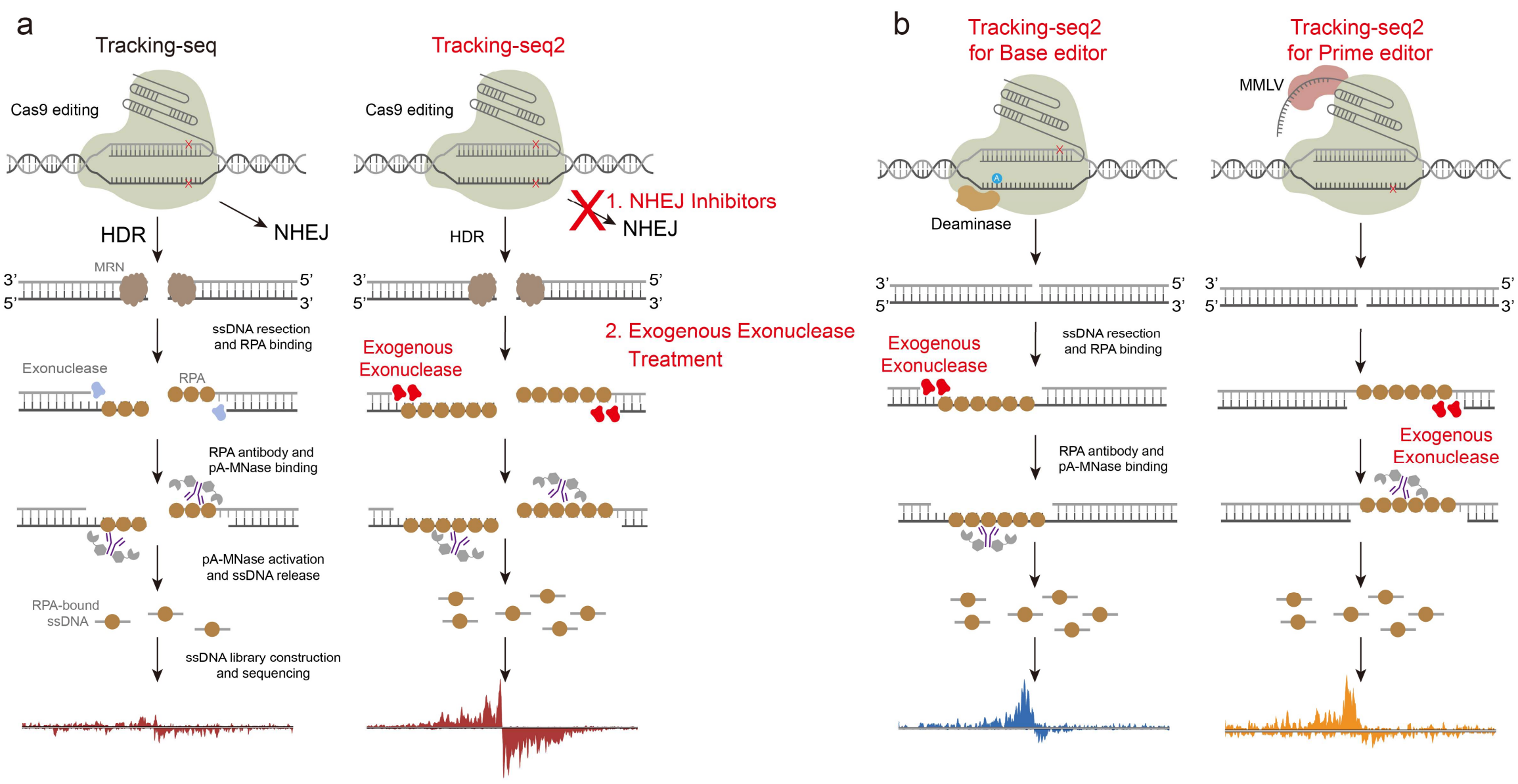

Supplementary Fig. 1. Schematic of genome-wide CRISPR off-target detection using Tracking-seq2. a, Schematic diagram illustrating the Tracking-seq2 strategy for modulating DNA repair pathways to enhance off-target detection. The approach incorporates two key interventions: (1) pharmacological inhibition of the NHEJ pathway using KU-60648 to favor homologous recombination (HR), thereby increasing the accumulation of RPA-bound single-stranded DNA (ssDNA) intermediates; and (2) application of exogenous T5 exonuclease (a 5′→3′ exonuclease) to process DNA ends and further amplify RPA-bound ssDNA signals. **b**, Schematic illustration of the Tracking-seq2 workflow for BEs and PEs. Exogenous T5 exonuclease enhances the off-target detection sensitivity for BEs and PEs. Note that NHEJ inhibition is not applied for BEs and PEs, as these editors generate SSBs rather than DSBs, and NHEJ is primarily involved in DSBs repair.

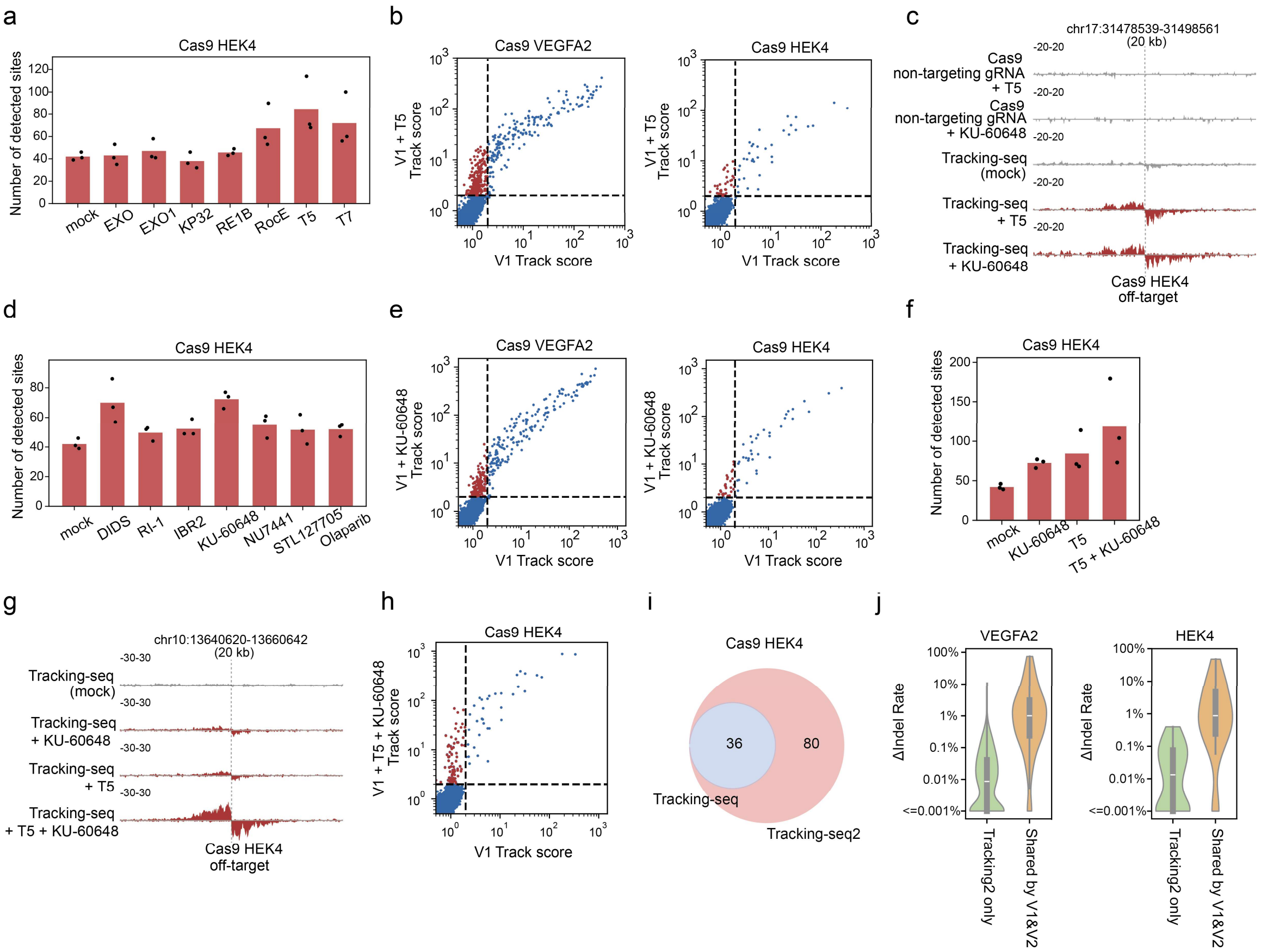

Supplementary Fig. 2. T5 and KU-60648 enhanced the sensitivity of Tracking-seq for detecting Cas9 and Cas12a off-targets. a, Numbers of off-target sites caused by Cas9 + sgRNA HEK4 detected by Tracking-seq co-transfected with various plasmids encoding 5′-3′ exonucleases. Mock: control group without exonuclease co-transfection. Each dot represents a biological replicate (n=3). **b**, Scatter plot showing the Track scores of Tracking-seq with T5 co-transfection versus those without co-transfection, edited by Cas9 + sgRNA VEGFA2 (left) and Cas9 + sgRNA HEK4 (right). Each dot represents a candidate off-target site. Dashed lines represent the thresholds of the Track score > 2 for positive sites. **c**, Signals generated by Tracking-seq at representative off-target sites caused by Cas9 + sgRNA HEK4. **d**, Numbers of off-target sites caused by Cas9 + sgRNA HEK4 detected by Tracking-seq treated with various chemical inhibitors. Mock: control group without inhibitor treatment. Each dot represents a biological replicate. **e**, Scatter plot showing the Track scores of Tracking-seq with KU-60648 treatment versus those without treatment, edited by Cas9 + sgRNA VEGFA2 (left) and Cas9 + sgRNA HEK4 (right). Each dot represents a candidate off-target site. Dashed lines represent the thresholds of the Track score > 2 for positive sites. **f**, Numbers of off-target sites caused by Cas9 + sgRNA HEK4. The combined treatment resulted in the highest sensitivity. **g**, Signals generated by Tracking-seq at representative off-target sites caused by Cas9 + sgRNA HEK4 in various conditions. **h**, Scatter plot showing the Track scores of Tracking-seq with combined T5 co-transfection and KU-60648 treatment versus those without treatment edited by Cas9 + sgRNA HEK4. Each dot represents a candidate off-target site. Dashed lines represent the thresholds of the Track score > 2 for positive sites. **i**, Venn diagram

comparing off-target sites (Cas9 + HEK4) detected by Tracking-seq without treatment (mock) or by Tracking-seq2 (combined treatment of T5 and KU-60648). **j**, Box plot showing the distribution of indel rates measured by hybrid capture targeted sequencing at off-target sites exclusively detected by Tracking-seq2 and shared by Tracking-seq and Tracking-seq2 for sgRNA VEGFA2 or sgRNA HEK4. Source data are provided as a Source Data file.

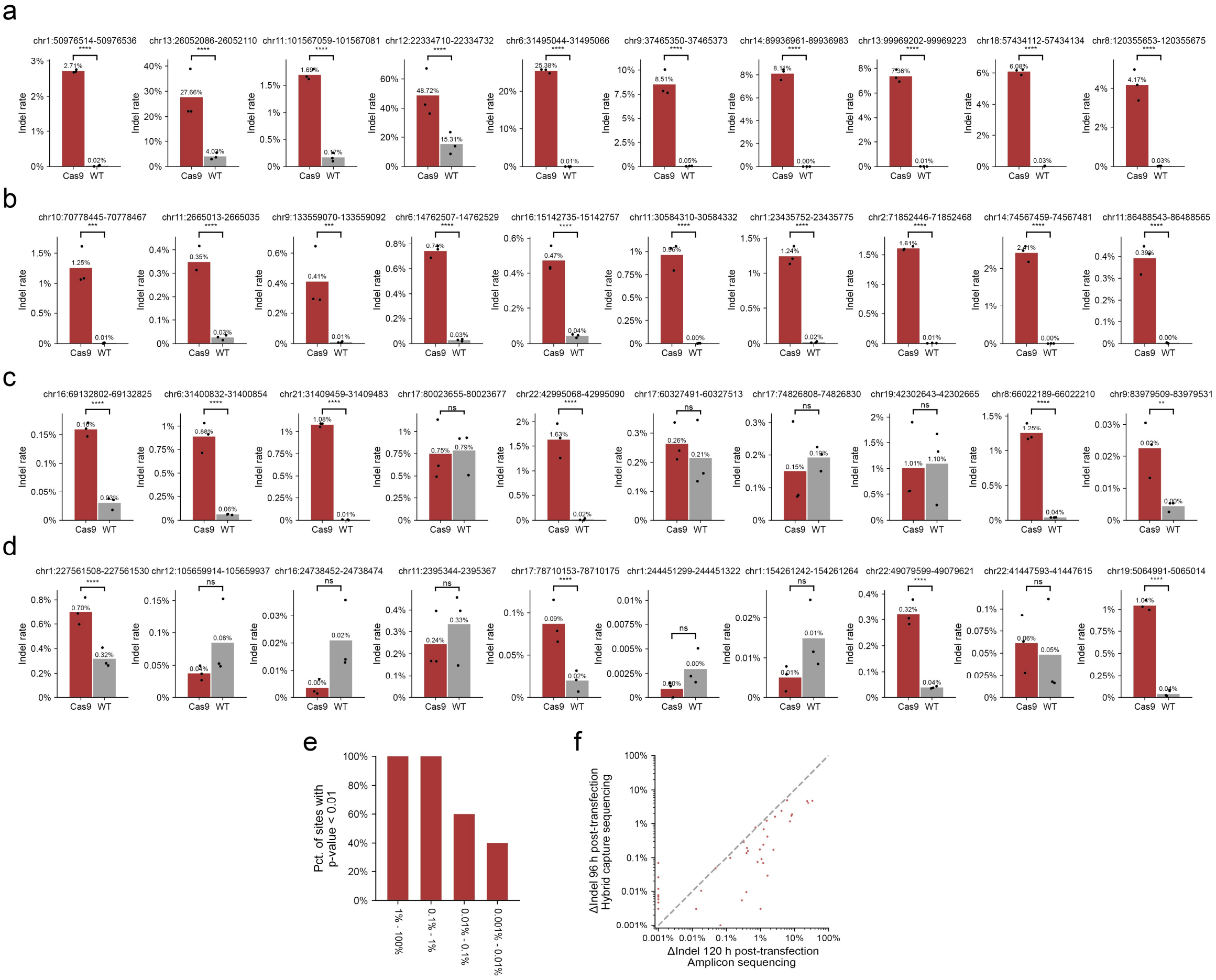

Supplementary Fig. 3. Validation of the hybrid capture sequencing results in different Δindel ranges. a-d, Bar plots showed the targeted amplicon sequencing validation (120 h post-transfection) of off-target sites with 1–10% (a), 0.1–1% (b), 0.01–0.1% (c), and 0.001–0.01% (d) Δindel rates detected by hybrid capture sequencing (96 h post-transfection). *P*-values were calculated by one-sided binomial tests without adjustment for multiple comparisons. Source Data and the exact *P*-values are listed in Supplementary Data 2. **e**, Bar plot displays the percentage of sites—within each Δindel rate bin previously determined by hybrid capture sequencing—that exhibited statistically significant editing (*p*-value < 0.01 versus WT control) in the targeted amplicon validation. **f**, Scatter plot comparing off-target Δindel rates measured by hybrid capture sequencing at 96 h post-editing versus targeted amplicon sequencing at 120 h post-editing. True off-target sites exhibited similar or higher Δindel rates as expected.

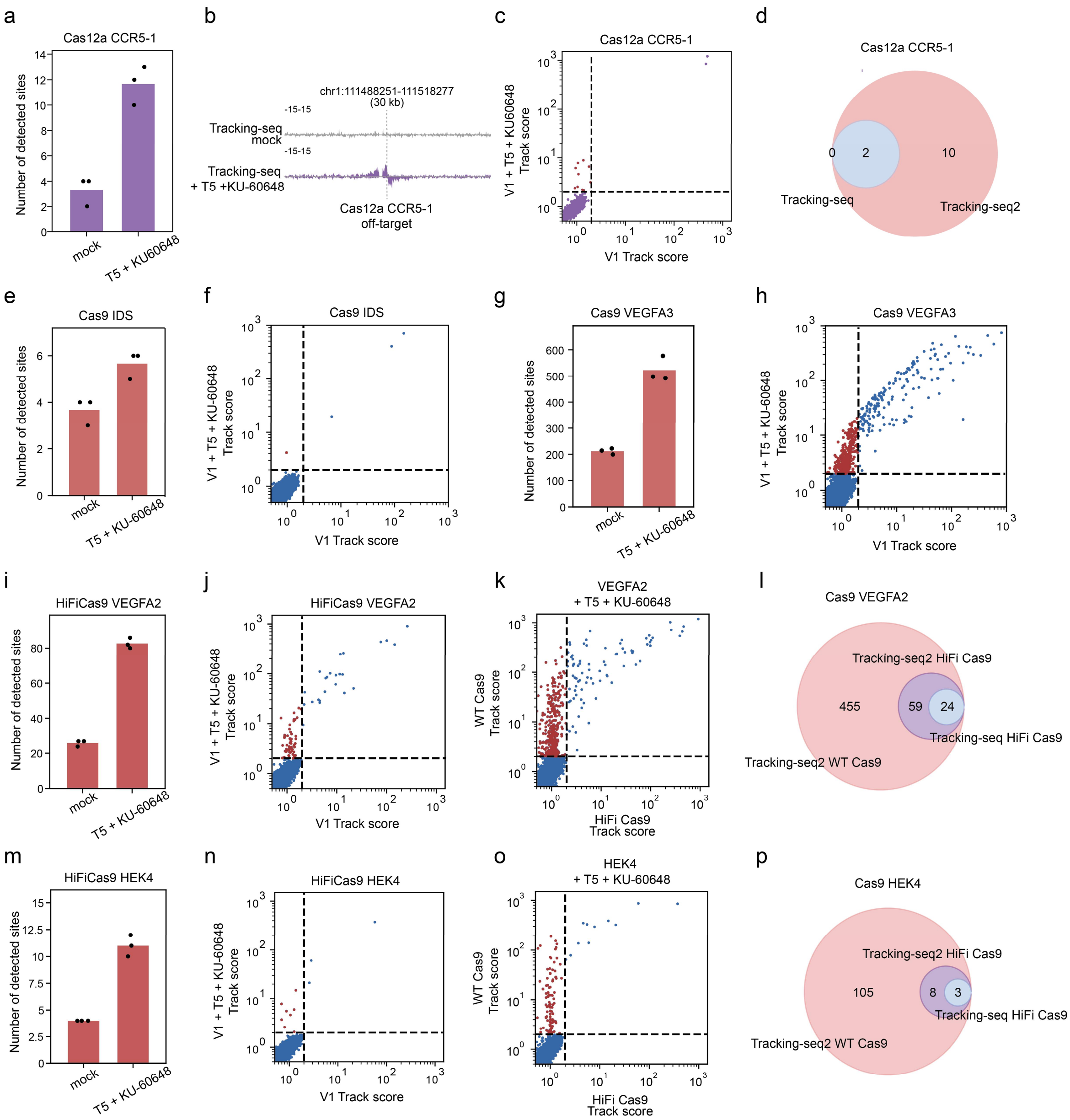

Supplementary Fig. 4. T5 exonuclease and KU-60648 enhance the sensitivity of Tracking-seq for detecting Cas12a, Cas9, and HiFi Cas9 off-target sites in HEK293T cells. a, Numbers of off-target sites caused by Cas12a + sgRNA CCR5-1 detected by Tracking-seq (mock) and Tracking-seq2 (T5 + KU-60648). Each dot represents an independent biological replicate (n=3). **b**, Signals generated by Tracking-seq/Tracking-seq2 at representative off-target sites. **c**, Scatter plot showing the Track scores of Tracking-seq and Tracking-seq2 (Cas12a + sgRNA CCR5-1). Each dot represents a candidate off-target site. Dashed lines represent the thresholds of the Track score > 2 for positive sites. **d**, Venn diagram comparing off-target sites detected by Tracking-seq and Tracking-seq2. **e**, Numbers of off-target sites detected by Tracking-seq and Tracking-seq2 (Cas9 + sgRNA IDS). Each dot represents an independent biological replicate (n=3). **f**, Scatter plot showing the Track scores of Tracking-seq and Tracking-seq2 (Cas9 + sgRNA IDS). **g**, Numbers of off-target sites detected by Tracking-seq and Tracking-seq2 (Cas9 + sgRNA VEGFA3). Each dot represents an independent biological replicate (n=3). **h**, Scatter plot showing the Track scores of Tracking-seq

and Tracking-seq2 (Cas9 + sgRNA VEGFA3). **i**, Numbers of off-target sites detected by Tracking-seq and Tracking-seq2 (HiFi Cas9 + sgRNA VEGFA2). Each dot represents an independent biological replicate (n=3). **j**, Scatter plot showing the Track scores of Tracking-seq and Tracking-seq2 (HiFi Cas9 + sgRNA VEGFA2). **k**, Scatter plots comparing the Track score of off-target sites caused by Cas9 and those caused by HiFi Cas9, in HEK293T cells (VEGFA2). **l**, Venn diagram comparing off-target sites generated by Cas9 or HiFi Cas9 + VEGFA2, detected by Tracking-seq and Tracking-seq2. **m**, Numbers of off-target sites detected by Tracking-seq and Tracking-seq2 (HiFi Cas9 + sgRNA HEK4). Each dot represents an independent biological replicate (n=3). **n**, Scatter plot showing the Track scores of Tracking-seq and Tracking-seq2 (HiFi Cas9 + sgRNA HEK4). **o**, Scatter plots comparing the Track scores of off-target sites caused by Cas9 and those caused by HiFi Cas9, in HEK293T cells (HEK4). **p**, Venn diagram comparing off-target sites detected by Tracking-seq and by Tracking-seq2 (Cas9 or HiFi Cas9 + sgRNA HEK4). Source data are provided as a Source Data file.

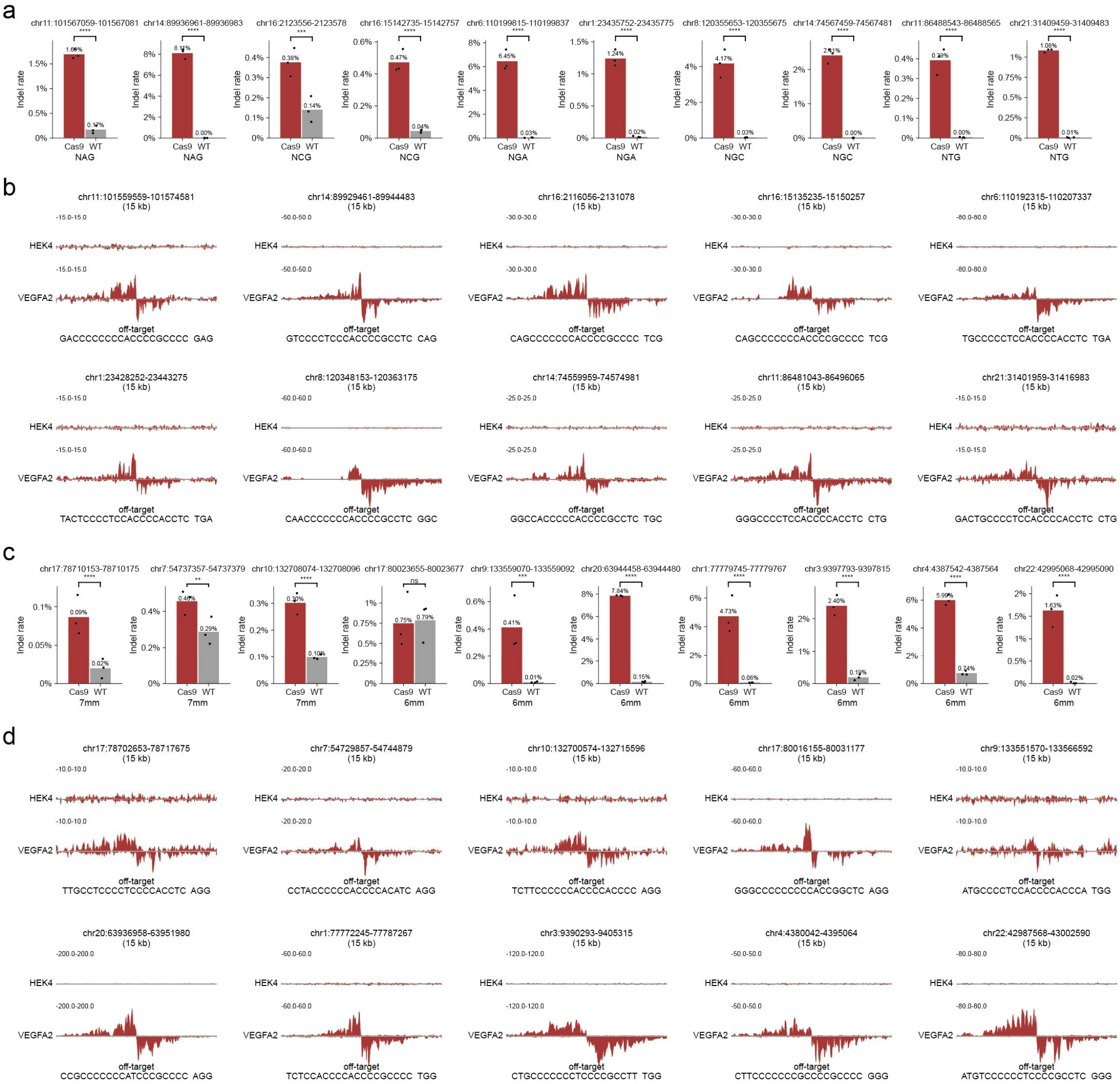

Supplementary Fig. 5. Validation of off-target sites with non-canonical PAMs and high mismatches detected by Tracking-seq2. a, Targeted amplicon sequencing validation of off-target sites with non-canonical PAMs detected by Tracking-seq2 for Cas9 + sgRNA VEGFA2 in HEK293T cells. Five non-canonical PAMs (NAG, NCG, NTG, NGA, and NGC) were identified, and two representative off-target sites for each type were validated. All tested sites exhibited bona fide off-target editing. *P*-values were calculated by one-sided binomial tests without adjustment for multiple comparisons. Source Data and the exact *P*-values are listed in Supplementary Data 2. **b**, Tracking-seq2 signals at representative off-target sites with non-canonical PAMs for Cas9 + sgRNA VEGFA2 in HEK293T cells. Distinct signals confirmed off-target cleavage with all five non-canonical PAM types. Cas9 + sgRNA HEK4 served as a non-targeting control. **c**, Targeted amplicon sequencing validation of off-target sites with 6 or 7 mismatches detected by Tracking-seq2 for Cas9 + sgRNA VEGFA2 in HEK293T cells. 9 out of 10 were verified as true off-target events. *P*-values were calculated by one-sided binomial tests without adjustment for multiple comparisons. Source Data and the exact *P*-values are listed in Supplementary Data 2. **d**, Tracking-seq2 signals at representative off-target sites with 6 or 7 mismatches for Cas9 + sgRNA VEGFA2 in HEK293T cells. Cas9 + sgRNA HEK4 served as a non-targeting control.

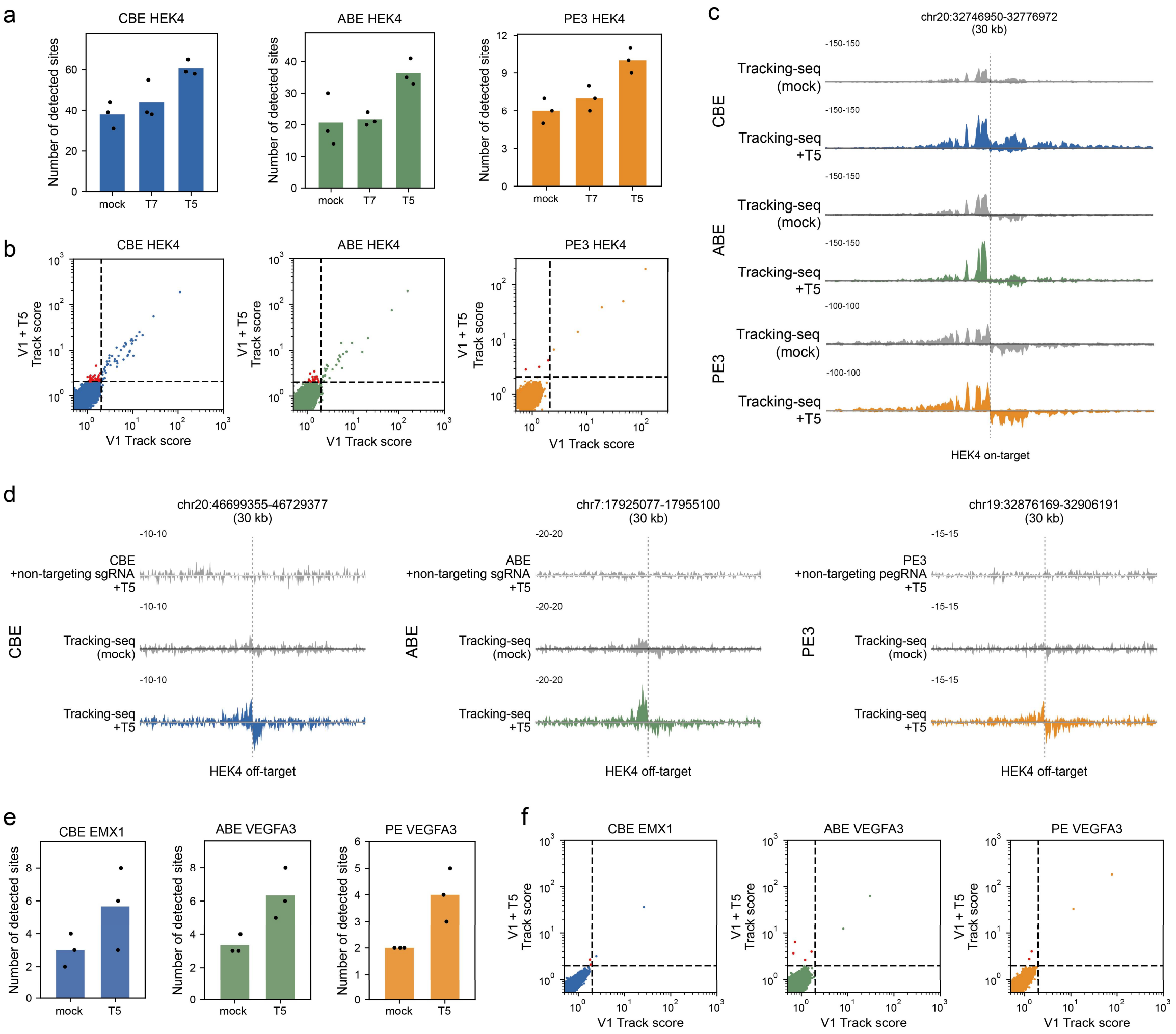

Supplementary Fig. 6. T5 enhanced the sensitivity of Tracking-seq for CBE, ABE, and PE in HEK293T cells. a, Numbers of off-target sites caused by CBE, ABE, or PE3 using sgRNA/pegRNA HEK4 detected by Tracking-seq co-transfected with plasmids encoding T5, T7, or mock. Each dot represents a biological replicate (n=3). **b**, Scatter plot showing the Track scores of Tracking-seq with T5 co-transfection versus those without co-transfection, edited by CBE, ABE, or PE3 with sgRNA/pegRNA HEK4. Each dot represents a candidate off-target site. Dashed lines represent the thresholds of the Track score > 2 for positive sites. **c-d**, Signals generated by Tracking-seq at the on-target site (c) and representative off-target sites (d) of sgRNA/pegRNA HEK4. The range in the top left of each track denotes normalized signal intensity, represented as read coverage per 10 million mapped reads. **e**, Numbers of off-target sites caused by CBEmax + sgRNA EMX1, ABE + sgRNA VEGFA3, PE3 + pegRNA VEGFA3, and detected by Tracking-seq in mock group and combined treatment of T5 and KU-60648 group. Each dot represents an independent biological replicate (n=3). **f**, Scatter plot showing the Track scores of Tracking-seq with T5 co-transfection versus those without treatment edited by CBEmax + sgRNA EMX1, ABE + sgRNA VEGFA3, PE3 + pegRNA VEGFA3, respectively. Each dot represents a candidate off-target site. Dashed lines represent the thresholds of the Track score > 2 for positive sites. Source data are provided as a Source Data file.

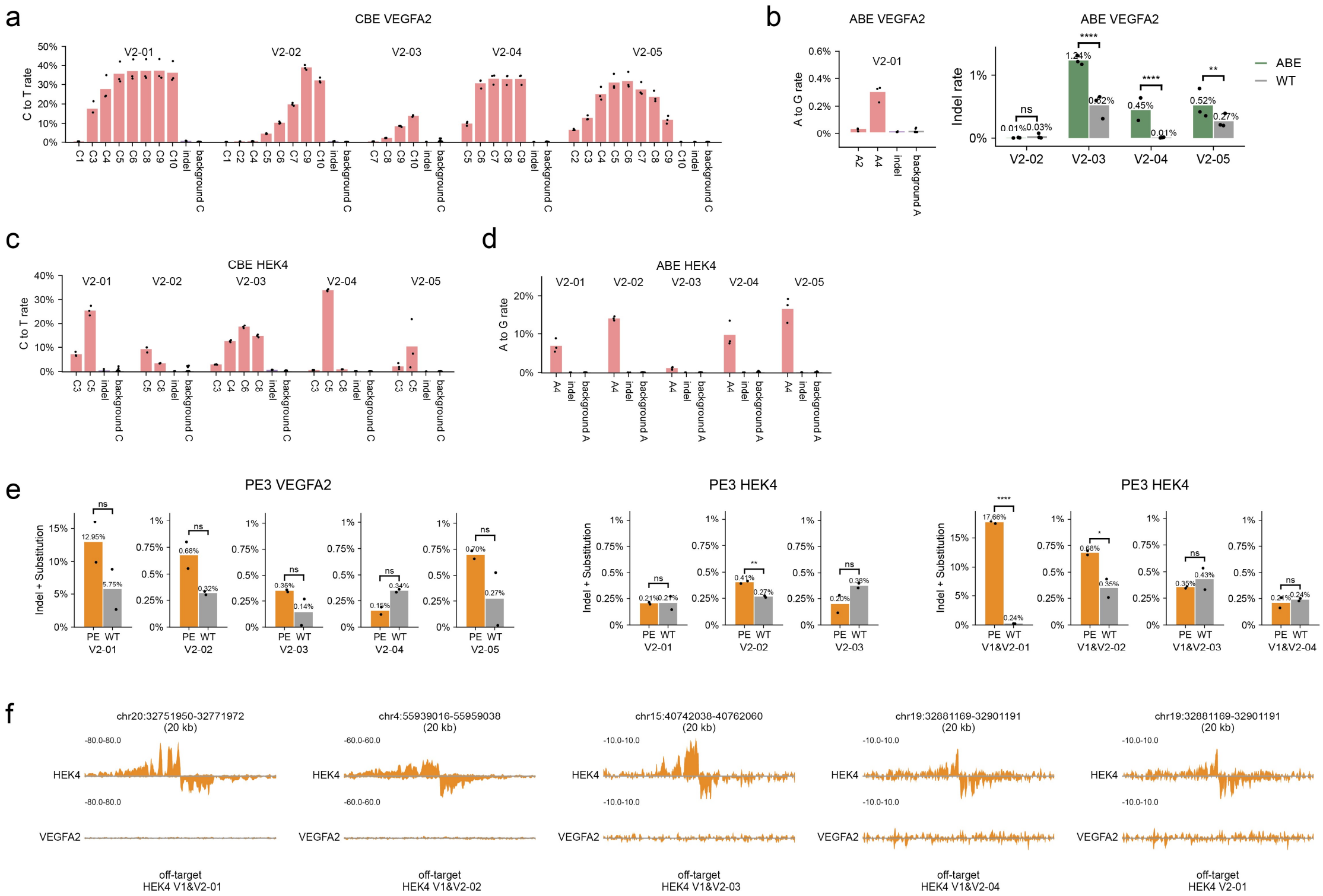

Supplementary Fig. 7. Validation of novel off-target sites detected by Tracking-seq2 for base editors and prime editors. a-d, Targeted amplicon sequencing validation of the top five novel off-target sites detected by Tracking-seq2 for CBE + sgRNA VEGFA2 (a), ABE + sgRNA VEGFA2 (b), CBE + sgRNA HEK4 (c), and ABE + sgRNA HEK4 (d) in HEK293T cells. All tested sites exhibited C-to-T editing within the editing window for CBE + sgRNA VEGFA2 or HEK4. For ABE + sgRNA VEGFA2 or HEK4, all tested sites with adenines within the editing window exhibited A-to-G editing, while 3/4 sites lacking adenines in the editing window exhibited low-frequency indels. *P*-values were calculated by one-sided binomial tests without adjustment for multiple comparisons. Source Data and the exact *P*-values are listed in Supplementary Data 3. Background C/A: C or A outside the editing window. **e**, Targeted amplicon sequencing validation of off-target sites for PE3. Novel off-target sites for PE3 + pegRNA VEGFA2 (left) and for PE3 + pegRNA HEK4 (middle) and four sites jointly detected by both Tracking-seq V1 and V2 for PE3 + pegRNA HEK4 (right). The majority of tested sites exhibited no significant editing, consistent with the high fidelity of prime editors. Only the site with the strongest Tracking-seq2 signal (V1&V2-01) contained substantial off-target editing. *P*-values were calculated by one-sided binomial tests without adjustment for multiple comparisons. Source Data and the exact *P*-values are listed in Supplementary Data 3. **f**, Tracking-seq2 signals at representative off-target sites for PE3 + pegRNA HEK4. PE3 + pegRNA VEGFA2 serves as a non-targeting control. Distinct signals were observed at these off-target sites lacking detectable mutations, suggesting that DNA nicking events occurred at these loci but are efficiently repaired in an error-free manner.

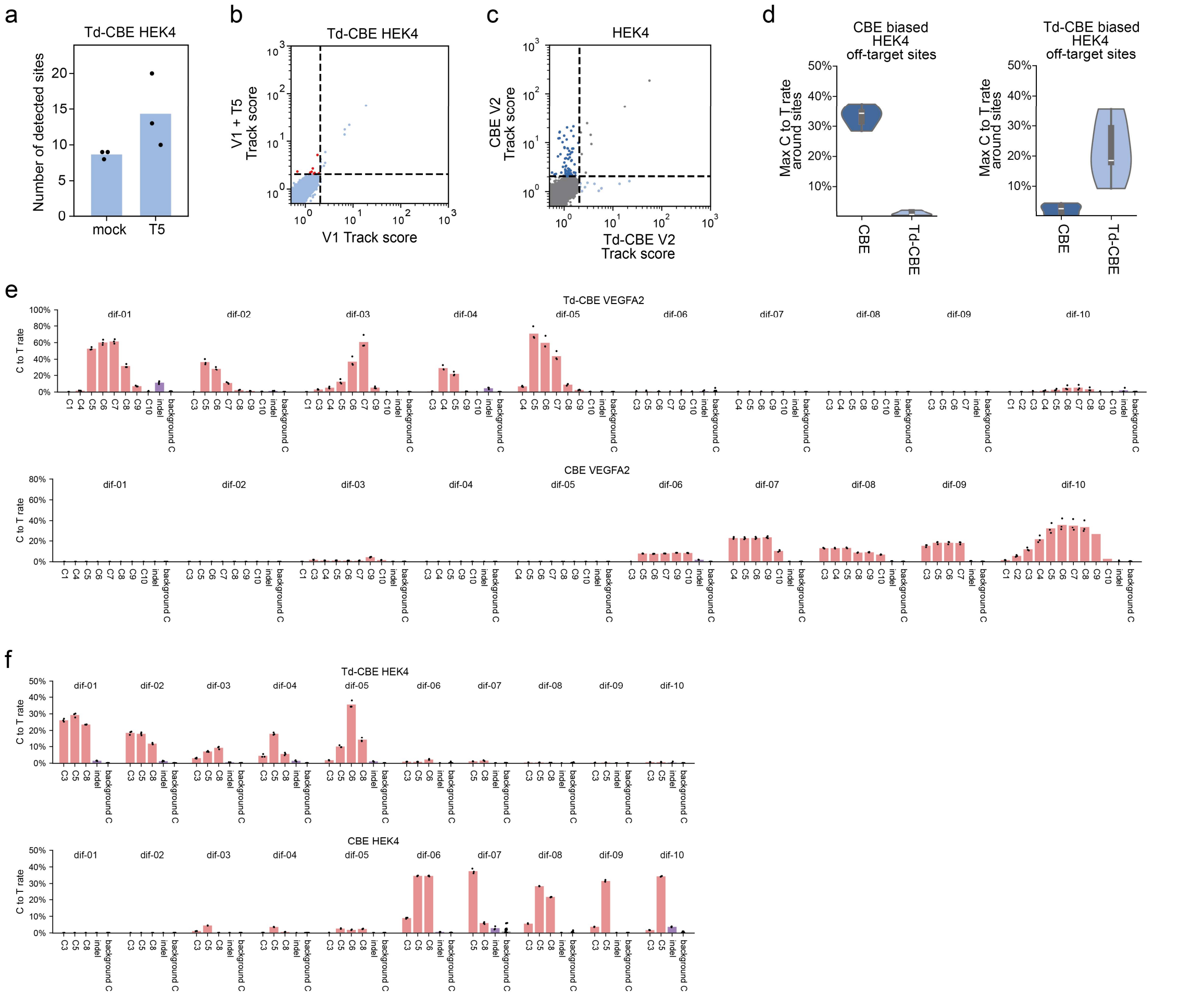

Supplementary Fig. 8. Td-CBEmax and CBE4max exhibited mutually exclusive biases of off-target sites. a, Numbers of off-target sites caused by Td-CBEmax + sgRNA HEK4 detected by Tracking-seq in mock group and T5 group. Each dot represents a biological replicate (n=3). **b**, Scatter plot showing the Track scores of Tracking-seq with T5 co-transfection treatment versus those without treatment edited by Td-CBEmax + sgRNA HEK4. Each dot represents a candidate off-target site. Dashed lines represent the thresholds of the Track score > 2 for positive sites. **c**, Scatter plots comparing the Track scores of off-target sites detected for CBE4max and Td-CBEmax in HEK293T cells using sgRNA HEK4. **d**, Box plots showing the maximum C-to-T editing efficiency of 5 tested CBE4max-biased off-target sites (left) and 5 tested Td-CBEmax-biased off-target sites (right) in HEK293T cells (sgRNA HEK4). **e-f**, Bar chart showing the C-to-T conversion rates at 5 Td-CBEmax-biased off-target sites (dif-01 to dif-05) and 5 CBE4max-biased off-target sites (dif-06 to dif-10) generated with sgRNA VEGFA2 (**e**) or sgRNA HEK4 (**f**) for the two versions of CBE. Background C: C outside the editing window. Source data are provided as a Source Data file.

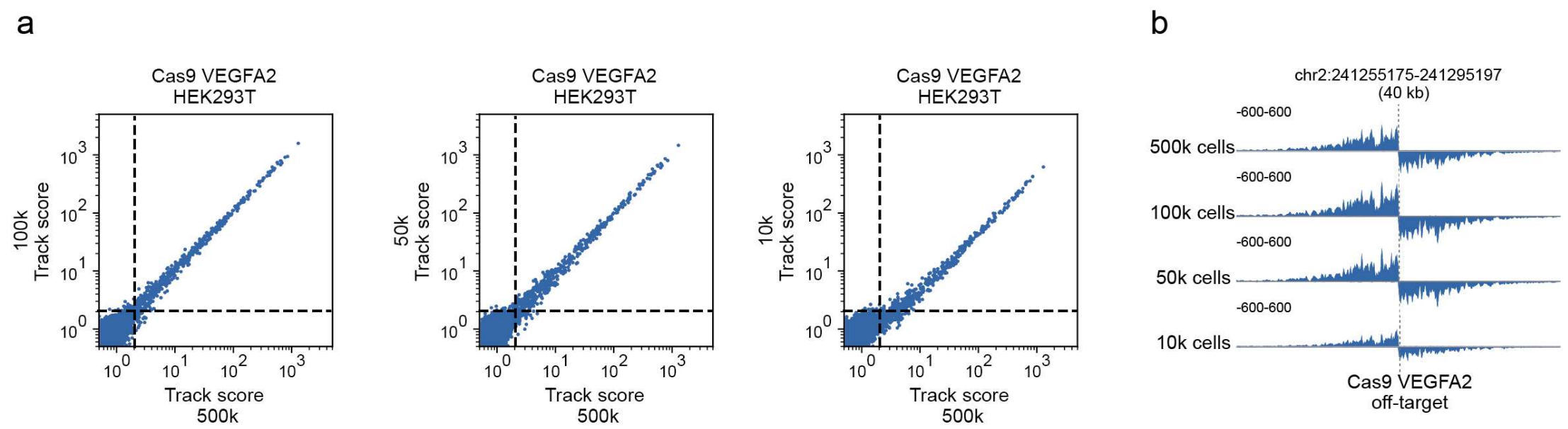

Supplementary Fig. 9. Evaluation of Tracking-seq2 sensitivity across different numbers of HEK293T cells. a, Correlation between Track scores in experiments with different HEK293T cells (10,000, 50,000, 100,000, or 500,000 cells) edited by Cas9 + sgRNA VEGFA2. Each dot represents a candidate off-target site. Dashed lines represent the thresholds of the Track score > 2 for positive sites. Sensitivity with 100,000 or 50,000 cells was similar to that with 500,000 cells, while assays using 10,000 cells missed off-target sites with low signals. **b**, Tracking-seq signals at a representative off-target site using different numbers of HEK293T cells.

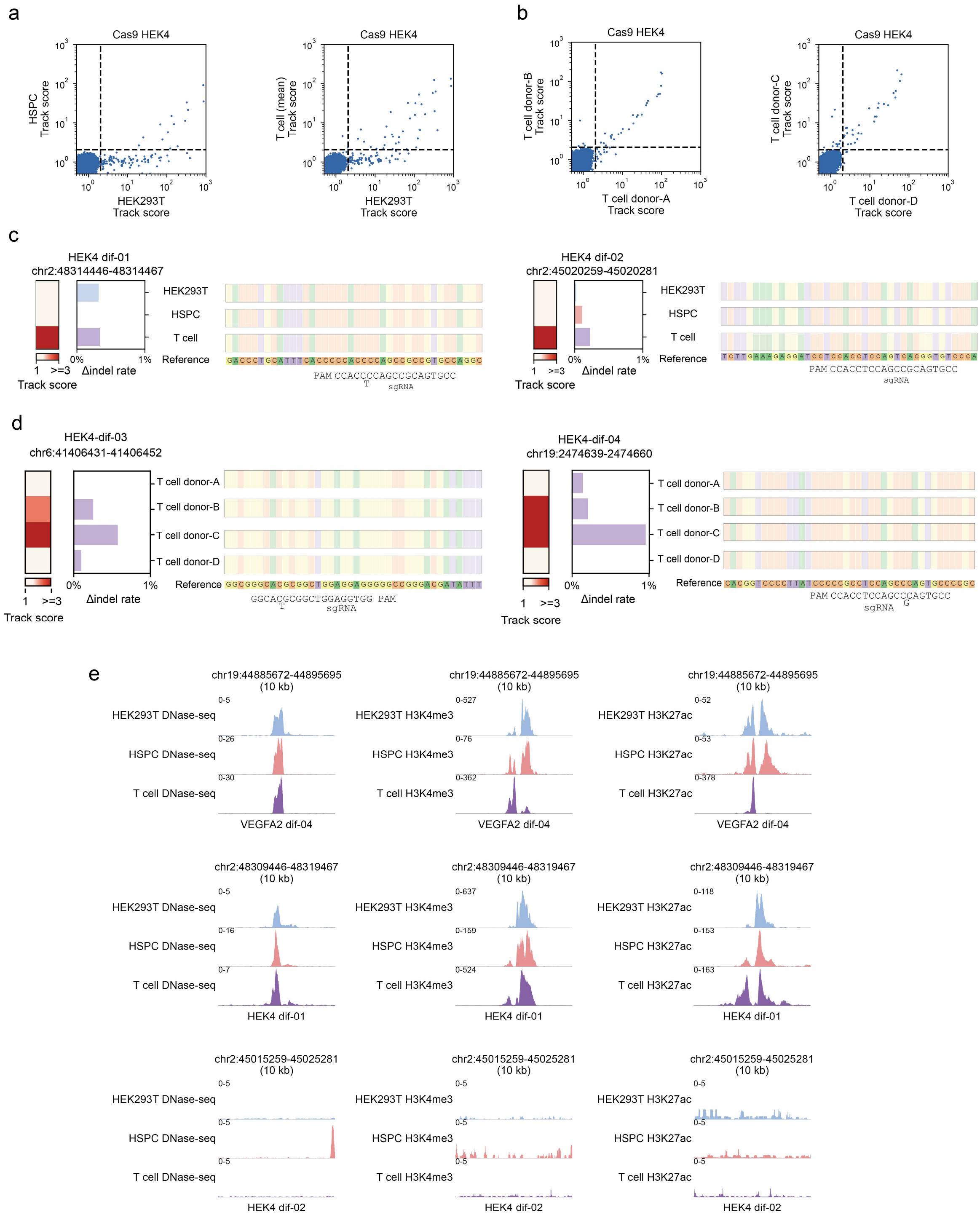

Supplementary Fig. 10. Genomic variations cannot account for all the off-target heterogeneity observed between different cell types and individuals. a, Scatter plot comparing the Track scores in HEK293T cells with HSPCs and T cells for the sgRNA HEK4. **b**, Scatter plot comparing the Track scores across different donors for the sgRNA HEK4. **c-d**, Genomic sequences of unedited HEK293T cells, HSPCs, and T cells from four different donors at the identified differential off-target sites for the sgRNA HEK4, as determined by targeted amplicon sequencing. The genomic sequences across all cell types (c) and all T cell donors (d) were homozygous and completely identical. These findings indicate that genomic variations cannot fully explain the

observed off-target heterogeneity. **e**, DNase-seq, H3K4me3 ChIP-seq, and H3K27ac ChIP-seq profiles of three cell types at differential off-target sites of sgRNA VEGFA2 and sgRNA HEK4. No obvious correlation was found with the off-target editing differences.
