# Supplementary material for: Ultra-sensitive profiling of CRISPR-Cas off-target effects with Tracking-seq2
Source: Nat Commun. 2026 Aug 11;17:9659. doi: 10.1038/s41467-026-76778-9 (PMC13558771; doi:10.1038/s41467-026-76778-9)
Supplement: Supplementary file 2 [file 41467_2026_76778_MOESM2_ESM.pdf]

Description of Additional Supplementary Files

File Name: Supplementary Data 1
Description: Track scores of off-target sites.xlsx

File Name: Supplementary Data 2
Description: Cas9 benchmarking and editing efficiency of off-targets.xlsx

File Name: Supplementary Data 3
Description: BEs and PEs editing efficiency of off-targets.xlsx

File Name: Supplementary Data 4
Description: gRNA sequences.xlsx
