# Supplementary material for: Ultra-sensitive profiling of CRISPR-Cas off-target effects with Tracking-seq2
Source: Nat Commun. 2026 Aug 11;17:9659. doi: 10.1038/s41467-026-76778-9 (PMC13558771; doi:10.1038/s41467-026-76778-9)
Supplement: Supplementary file 3 [file 41467_2026_76778_MOESM7_ESM.pdf]

nature portfolio

Corresponding author(s): Ming Zhu

Last updated by author(s): Jun 23, 2026

Reporting Summary

Nature Portfolio wishes to improve the reproducibility of the work that we publish. This form provides structure for consistency and transparency in reporting. For further information on Nature Portfolio policies, see our Editorial Policies and the Editorial Policy Checklist.

Statistics

For all statistical analyses, confirm that the following items are present in the figure legend, table legend, main text, or Methods section.

n/a	Confirmed	
☐	☒	The exact sample size (n) for each experimental group/condition, given as a discrete number and unit of measurement
☐	☒	A statement on whether measurements were taken from distinct samples or whether the same sample was measured repeatedly
☐	☒	The statistical test(s) used AND whether they are one- or two-sided<br>*Only common tests should be described solely by name; describe more complex techniques in the Methods section.*
☒	☐	A description of all covariates tested
☒	☐	A description of any assumptions or corrections, such as tests of normality and adjustment for multiple comparisons
☐	☒	A full description of the statistical parameters including central tendency (e.g. means) or other basic estimates (e.g. regression coefficient) AND variation (e.g. standard deviation) or associated estimates of uncertainty (e.g. confidence intervals)
☐	☒	For null hypothesis testing, the test statistic (e.g. F, t, r) with confidence intervals, effect sizes, degrees of freedom and P value noted<br>*Give P values as exact values whenever suitable.*
☒	☐	For Bayesian analysis, information on the choice of priors and Markov chain Monte Carlo settings
☒	☐	For hierarchical and complex designs, identification of the appropriate level for tests and full reporting of outcomes
☒	☐	Estimates of effect sizes (e.g. Cohen's d, Pearson's r), indicating how they were calculated

Our web collection on statistics for biologists contains articles on many of the points above.

Software and code

Policy information about availability of computer code

Data collection	No code was used for data collection.
Data analysis	chromap (v0.3.0); CRISPResso2 (v2.3.2); CRISPRessoWGS (v2.3.2); statsmodels (v0.14.5); bedtools (v2.30.0); bedGraphToBigWig (v4); Offtracker (v2.14.5) https://github.com/Lan-lab/offtracker ; GUIDE-seq-2(v2.0.0) https://github.com/tsailabSJ/guideseq

For manuscripts utilizing custom algorithms or software that are central to the research but not yet described in published literature, software must be made available to editors and reviewers. We strongly encourage code deposition in a community repository (e.g. GitHub). See the Nature Portfolio guidelines for submitting code & software for further information.

Data

Policy information about availability of data

All manuscripts must include a data availability statement. This statement should provide the following information, where applicable:

- Accession codes, unique identifiers, or web links for publicly available datasets
- A description of any restrictions on data availability
- For clinical datasets or third party data, please ensure that the statement adheres to our policy

The data of HEK293T cells generated in this study have been deposited in NCBI GEO database under accession code GSE307225 [https://www.ncbi.nlm.nih.gov/geo/query/acc.cgi?acc=GSE307225]. The raw data and processed data of human primary cells have been deposited in GSA-Human database under accession code HRA013027 [https://ngdc.cncb.ac.cn/gsa-human/browse/HRA013027] and OMIX database under accession code OMIX011731 [https://ngdc.cncb.ac.cn/omix/

release/OMIX011731], respectively. For epigenetic data, we downloaded the bigwig files from the ENCODE portal with the following identifiers: ENCFF148BGE [https://www.encodeproject.org/files/ENCFF148BGE], ENCFF416AAY [https://www.encodeproject.org/files/ENCFF416AAY], ENCFF719ZZK [https://www.encodeproject.org/files/ENCFF719ZZK], ENCFF315TAU [https://www.encodeproject.org/files/ENCFF315TAU], ENCFF795ZQG [https://www.encodeproject.org/files/ENCFF795ZQG], ENCFF384CAD [https://www.encodeproject.org/files/ENCFF384CAD], ENCFF157TGK [https://www.encodeproject.org/files/ENCFF157TGK], ENCFF537GCQ [https://www.encodeproject.org/files/ENCFF537GCQ], and ENCFF314OSR [https://www.encodeproject.org/files/ENCFF314OSR], for DNase-seq, H3K4me3 ChIP-seq, and H3K27ac ChIP-seq of HEK293T cells, HSPCs and T cells. Off-target results and other data are available in the Supplementary Data files and the Source Data file. Source data are provided with this paper.

Research involving human participants, their data, or biological material

Policy information about studies with human participants or human data. See also policy information about sex, gender (identity/presentation), and sexual orientation and race, ethnicity and racism.

Reporting on sex and gender	There is no human participant in this study.
Reporting on race, ethnicity, or other socially relevant groupings	There is no human participant in this study.
Population characteristics	There is no human participant in this study.
Recruitment	There is no human participant in this study.
Ethics oversight	There is no human participant in this study.

Note that full information on the approval of the study protocol must also be provided in the manuscript.

Field-specific reporting

Please select the one below that is the best fit for your research. If you are not sure, read the appropriate sections before making your selection.

☒ Life sciences ☐ Behavioural & social sciences ☐ Ecological, evolutionary & environmental sciences

For a reference copy of the document with all sections, see nature.com/documents/nr-reporting-summary-flat.pdf

Life sciences study design

All studies must disclose on these points even when the disclosure is negative.

Sample size	No statistical method was used to predetermine sample size. Key experiments were performed with 3 biological replicates for each condition, as n=3 is widely accepted in the field.
Data exclusions	In the targeted amplicon sequencing and hybrid capture targeted sequencing data analysis, off-target sites with mapping rates lower than 10% or mapped reads lower than 1000 were regarded as failures and excluded.
Replication	All attempts at replication for experimental findings were successful.
Randomization	Allocation of samples was random.
Blinding	The investigators were not blinded to allocation during experiments and outcome assessment, as the inherently distinct protocols of different off-target detection methods preclude blinding during execution, but all data were processed with pre-established pipelines.

Reporting for specific materials, systems and methods

We require information from authors about some types of materials, experimental systems and methods used in many studies. Here, indicate whether each material, system or method listed is relevant to your study. If you are not sure if a list item applies to your research, read the appropriate section before selecting a response.

Materials & experimental systems

n/a	Involved in the study
☐	☒ Antibodies
☐	☒ Eukaryotic cell lines
☒	☐ Palaeontology and archaeology
☒	☐ Animals and other organisms
☒	☐ Clinical data
☒	☐ Dual use research of concern
☒	☐ Plants

Methods

n/a	Involved in the study
☒	☐ ChIP-seq
☐	☒ Flow cytometry
☒	☐ MRI-based neuroimaging

Antibodies

Antibodies used: Anti-Replication Protein A Antibody (1:100, MABE285, Millipore)

Validation: Anti-Replication Protein A Antibody previously validated in 18 studies (https://www.sigmaaldrich.cn/CN/zh/search/mabe285?focus=papers&page=1&perpage=30&sort=relevance&term=mabe285&type=citation_search)

Eukaryotic cell lines

Policy information about cell lines and Sex and Gender in Research

Cell line source(s): HEK293T cells (ATCC, CRL-3216)

Authentication: The cell line was not formally authenticated by the authors. The appearance and growth characteristics matched expectations.

Mycoplasma contamination: The cell line tested negative for mycoplasma contamination.

Commonly misidentified lines (See ICLAC register): None.

Plants

Seed stocks: No plants were used.

Novel plant genotypes: No plants were used.

Authentication: No plants were used.

Flow Cytometry

Plots

Confirm that:

☒ The axis labels state the marker and fluorochrome used (e.g. CD4-FITC).

☒ The axis scales are clearly visible. Include numbers along axes only for bottom left plot of group (a 'group' is an analysis of identical markers).

☒ All plots are contour plots with outliers or pseudocolor plots.

☒ A numerical value for number of cells or percentage (with statistics) is provided.

Methodology

Sample preparation: Both wild-type (WT) and transfected cells were treated with 0.25% Trypsin at room temperature for 1 minute, followed by quenching using culture medium. The cells were subsequently suspended in DPBS for flow cytometry.

Instrument: Flow cytometric analysis and cell sorting were performed on BD Influx.

Software	FlowJo v.10 was used for flow cytometry data analysis.
Cell population abundance	For CRISPR/Cas9 editing experiments, the Cas9-expressing cells were GFP-positive. The abundance of the GFP+ population was >50% among CRISPR/Cas9-edited group and 0% among the WT group, respectively.
Gating strategy	In all experiments, debris was initially excluded by the morphology gate utilizing FSC-A and SSC-A parameters. Non-singlet events were subsequently excluded by a single cell gate based on FSC-A and FSC-Width. FITC-positive cells from the transfected groups were gated using the baseline fluorescence of the WT group as the reference.

☒ Tick this box to confirm that a figure exemplifying the gating strategy is provided in the Supplementary Information.
