# Supplementary material for: Ultra-sensitive profiling of CRISPR-Cas off-target effects with Tracking-seq2
Source: Nat Commun. 2026 Aug 11;17:9659. doi: 10.1038/s41467-026-76778-9 (PMC13558771; doi:10.1038/s41467-026-76778-9)
Supplement: Supplementary file 4 [file 41467_2026_76778_MOESM8_ESM.pdf]

nature portfolio

Peer Review File

Ultra-sensitive profiling of CRISPR-Cas off-target effects with Tracking-seq2

Corresponding Author: Professor Ming Zhu

Parts of this Peer Review File have been redacted as indicated to remove third-party material.

This file contains all reviewer reports in order by version, followed by all author rebuttals in order by version.

Version 0:

Reviewer comments:

Reviewer #1

(Remarks to the Author)
Summary and Overall Assessment

In this manuscript, the authors present Tracking-seq2, which they describe as an improved, broadly applicable platform for genome-wide detection of off-target editing events. While the topic is timely and relevant to the genome-editing community, this reviewer finds that the study does not provide sufficient benchmarking or methodological rigor to justify the central claims of superiority over existing tools. The comparison set is also highly problematic: Cas-OFFinder and CIRCLE-Seq are well documented to yield high rates of false positives relative to more high-fidelity approaches such as GUIDE-Seq and COSMID. As a result, the work appears to offer only incremental improvements over both these existing methods and the authors' own Tracking-seq1 system, and does not substantively address key emerging concerns in the field such as detection of large genomic abnormalities.

Major Comments

1. Conceptual framing of "improvement."
The authors assume that identifying a greater number of putative off-target sites represents an intrinsic improvement in detection capability. However, in practice, the vast majority of such sites reported by methods including CIRCLE-Seq, SITE-Seq, and GUIDE-Seq are false positives that do not validate by targeted deep sequencing in clinically relevant systems (e.g., transient delivery of Cas9, base editor, or mRNA RNPs to human primary HSPCs). Given that a single SNP within a protospacer can ablate editing, it is implausible that sites with up to seven mismatches (Fig. 2) represent true off-target events. Furthermore, the finding that "most Tracking-seq2-only sites contained non-NGG PAMs" is concerning, as Cas9 activity is well-established to be PAM-dependent. The authors should reconsider this premise and provide supporting validation data beyond the brief testing of Tracking-seq2 vs. Tracking-seq1 that is done in primary T cells and HSPCs.

2. Insufficient methodological detail.
Key experimental details are omitted or deferred to the Methods, impeding reader understanding. For example, Figures 1–2 do not specify the cell type used or whether experiments were cell-based or cell-free. Figure 1c is described only as showing "signals," without defining what is plotted. Similarly, the term "Track score" (Fig. 1g) is introduced without explanation. The manuscript would benefit from clearer figure legends and concise descriptions of experimental design throughout. In addition, nowhere are adequate details provided on the experiments in primary cells. For instance, what nucleofection system, buffer, and code was used for these experiments? Was the Cas9 high-fidelity which is now routinely included in genome editing trials as opposed to WT which has much higher off-target editing?

3. Benchmarking against unreliable comparators.
The authors benchmark Tracking-seq2 primarily against CIRCLE-Seq and Cas-OFFinder, both of which have been shown to exhibit low positive predictive value in clinically relevant contexts (Cromer et al., Mol Ther 2023). More meaningful benchmarking would include empirical, cell-based assays such as GUIDE-Seq and computational tools like COSMID, which more effectively balance sensitivity with specificity. Without these essential comparisons—particularly in human primary cells—to demonstrate that Tracking-seq2 identifies bona fide off-target sites that would be missed by these methods,

the authors' claims of superior performance are not supported. Clearly demonstrating how the reported modifications enhance sensitivity, reproducibility, or practical applicability is essential to justify publication in Nature Communications.

4. Dependence on prior Tracking-seq knowledge.
The manuscript presumes substantial familiarity with the authors' previous Tracking-seq1 work, providing insufficient detail on how Tracking-seq2 differs. This limits accessibility to new readers and weakens the authors' case for innovation. A clear summary of methodological differences and key optimizations should be added to the Introduction or early Results section.

5. Limit of detection.
The reported indel frequency of 0.005% (Fig. 1c) appears below reasonable detection thresholds. The authors should define and experimentally establish the assay's limit of detection and dynamic range.

6. Scope limited to small indels.
The study focuses exclusively on small indels and does not address large genomic alterations increasingly recognized as clinically relevant consequences of genome editing (e.g., large on-target deletions, chromosome arm loss). The fact that this is neither acknowledged in this paper is a major weakness.

7. Base and prime editing data interpretation.
The reported discrepancy between hundreds of off-target sites for Cas9 and only tens for base or prime editors is not adequately explained. Is this due to intrinsic differences in editing mechanisms or reduced assay sensitivity for single-strand or nicking systems? Additional clarification is warranted.

Minor Comments

1. Please remove overly definitive statements such as:
"Tracking-seq2 represents a significant advancement, establishing itself as the most sensitive, cell-based, and clinically applicable off-target detection technology to date."
"This enhancement significantly elevates the precision and reliability of safety evaluations in clinical genome editing."
Such claims are subjective and currently unsupported by comparative data.

2. Extended Data figure citations appear out of order (e.g., Extended Data Fig. 1l–o cited before 1j–k). Please correct the numbering and sequence of references throughout.

Overall Recommendation

While the manuscript describes a modification to an existing off-target detection platform, the current version lacks the benchmarking, methodological clarity, and novelty necessary to substantiate the authors' claims. To be considered a meaningful advance, Tracking-seq2 must be evaluated using more rigorous clinically relevant genome-editing workflows in human primary cells and directly demonstrate that it identifies bona fide off-target events missed by leading systems such as GUIDE-Seq and COSMID. Without such evidence, the work does not represent a substantive improvement over established approaches and is not suitable for publication in Nature Communications.

Reviewer #2

(Remarks to the Author)
Safe clinical translation of CRISPR-based therapeutics demands highly sensitive detection of off-target events directly within human cells. The manuscript titled "Ultra-sensitive profiling of CRISPR-Cas off-target effects with Tracking-seq2" describes an enhanced version of the Tracking-seq platform optimized for diverse CRISPR–Cas systems. Tracking-seq2 integrates T5 exonuclease treatment with inhibition of NHEJ DNA repair, thereby improving single-stranded DNA signal detection through RPA pull-down. According to the authors, these additional steps enhance sensitivity up to fivefold while maintaining high specificity.

Overall, the work presents a substantial technical advancement in off-target detection; however, it may be suitable for publication after the authors adequately address the following concerns and limitations:
1. The combined use of DNA repair inhibitors and exonuclease treatment is likely to perturb DNA repair kinetics. Figures 1G, 1K, 3B, and 4B (comparing T5-KU60648 track score vs track score mock) indicate high false discovery rate. The authors should quantify how many additional authentic off-targets (track score mock) Tracking-seq2 identifies when benchmarked against the original Tracking-seq1. It remains unclear to readers that users of Tracking-seq2 must perform two separate rounds of experiments—one using exonuclease and NHEJ inhibition to identify candidate off-targets, and another round without these modulators to confirm the true sites through targeted deep sequencing. The authors should discuss whether this workflow provides a practical advantage over Tracking-seq1. Moreover, similar validation using the "track score mock" approach was not performed for base editors and prime editors and should be included.
2. The manuscript refers to Tracking-seq2 as "unprecedentedly sensitive," "the most sensitive to date," and yet benchmarking is restricted to in silico tools and cell-free assays such as CIRCLE-seq, using only VEGFA2 and HEK4 guides. No direct comparisons with established in-cell methods (e.g., GUIDE-seq) or clinically relevant guides are

presented. These claims should be either moderated or supported by appropriate experiments.
3. The timing of sample collection (24 h and 48 h post-plasmid transfection) could influence outcomes. Since plasmid-driven Cas expression can persist beyond 48 h, additional off-target events might occur at later time points once intended target sites are modified. This possibility should be discussed.
4. The validation is based solely on indel frequency as a marker of genuine off-target activity. However, CRISPR off-target events can also result in chromosomal rearrangements, structural variations (e.g., translocations, inversions, copy number changes), or transcriptional alterations not captured by indel analysis. These unaccounted effects need to be acknowledged as limitations.
5. The authors state that Tracking-seq2 supports patient-specific safety testing using as few as 10,000–50,000 primary cells, while the Methods section specifies approximately 500,000 cells per experiment. This inconsistency requires clarification.
6. The computational pipeline is insufficiently described. Critical aspects such as “track score” calculation, peak calling strategy, background correction, replicate treatment, multimapping read management, and thresholding criteria should be explicitly detailed to ensure reproducibility.

7. The manuscript reports detection of robust Cas9 signals at sites with up to seven mismatches, suggesting extreme mismatch tolerance. This observation requires additional validation or discussion to clarify whether such sites truly reflect Cas9-mediated cleavage or arise from increased ssDNA generation under T5/NHEJ inhibition conditions.

8. Does the HSPCs and the T-cell comes from same donor? It may be important to show that HSPCs or T cells of two-three donors to support the claim the donor genotype altering the off-targets.
MINOR
Line 87 – Typo PARP1

Reviewer #3

(Remarks to the Author)
I recommend this manuscript for publishing pending several minor formatting changes. The researchers have substantially expanded upon the capabilities of TraskingSeq by implementing several known methods of increasing homologous recombination. Furthermore, Cong, Xu, Chen, Yuan , et al show the increased specificity of TrackingSeq2 when compared to in vitro off-target gene editing analysis software, and exhibit the need for such a tool in the rapidly-developing world of gene editing therapies.

Section-wise recommendations for changes are as follows:

Introduction:
The Introduction should suitably describe each of the cellular mechanisms that are relevant throughout the manuscript. There are several additions that, while not necessary, would improve communicability of the paper: these include brief descriptions of the proteins of interest in NHEJ and HR pathways (i.e. the role of RPA and Rad51 in ssDNA stability, the stabilizing effects of ssDNA donor templates, why 5’-3’ exonuclease treatment would affect HR, etc.), as well as including a brief description of the mechanism and protocol for the original TrackingSeq detection technology. One of the main goals of this manuscript is to outline how TrackingSeq2 is a significant advancement; adding a section to the introduction outlining how the current TrackingSeq assay is performed would be useful for this. Currently, TrackingSeq is described in the Introduction mainly to highlight its strengths compared to other analysis methods, which while accurate is better suited for the Discussion section.

Discussion:
Within the results, variability in the effectiveness of the assay changes is noted (i.e. Lines 145-147 denote how different target sites showed different changes in off-target detection with the addition of T5 exonuclease treatment. This points to a potentially major concern; if different sites are affected differently by the changes to the TrackingSeq2 method, then the changes introduced, while increasing sensitivity by amplifying rarer events to the point of detectability, are still insufficient to suitably replicate the action of a “real-time” CRISPR/Cas construct. This gap between the experimental conditions of the TrackingSeq2 gene editing reactions and other more complex and stochastic mechanisms at play in vivo should still be made clear.

Line-wise recommendations for changes are as follows:

Line 47 (Introduction): Please add a specific source(s) to support the statement “[Cell-free methods] exhibit poor clinical translatability due to fundamental discrepancies such as altered Cas9 kinetics in vitro and the lack of epigenetic context, therefore often suffers from high false positive rates.”

Line 80-86 (Results): This section discussing basic DSB repair mechanisms should be moved or otherwise included in the Introduction. The sentence in Line 86, “To identify potential inhibitors…” is a suitable start to this section of the Results, the rationale as to why should be in the Introduction.

Reviewer #4

(Remarks to the Author)
Dr. Cong and colleagues developed Tracking-seq2, an advanced technology that integrates 5'→3' exonuclease treatment and non-homologous end joining (NHEJ) pathway inhibitors with the original Tracking-seq.

Overall, the idea of the paper is interesting, but in its current form the work is not ready for publication in Nature Communications. Below are some overarching points that I believe are important to address in order to bring the study in line with the journal's standards:

• Increase the number of biological replicates in most experiments (at least n=5 for example in Fig. 1b, d, e, l, 3a, 4a, currently with n=1 in main figures).

• Include an appropriate time course analysis to dissect off target events generated by the different editing platforms, ideally over at least a 7 day experimental window (now just 24 or 48h post treatments in vitro).

• For CBEs, ABEs and PEs, target additional loci and, where possible, compare older versus newer editor versions, as the study currently uses CBE4max, which is not among the latest CBE variants.

• Test Tracking seq2 in vivo after several months, for example by measuring off targets in T cells or mainly in HSPCs edited ex vivo and then transplanted into immunodeficient mice. This experiment is extremely relevant to assess potential selection and expansion of edited clones in repopulating assays that better approximate human settings.

• Address off target detection in the context of homology-directed repair (HDR). It would be valuable to extend the method and discussion to HDR based therapeutic approaches. In particular, what is the impact of AAV6 mediated HDR on off target events, and how does this compare with the use of ssODN donors?

• Clarify whether HSPCs are derived from cord blood (CB) or mobilized peripheral blood (mPB), as editing efficiencies differ between these sources and may influence off target generation. I would suggest to compare off target profiles between CB and mPB derived HSPCs using Tracking seq28 at least in tow different genomic loci.

• Because each locus has a distinct chromatin environment that affects editing efficiency and off target formation, consider testing Tracking seq2 at additional loci edited with different platforms (e.g. CBE, ABE, PE, Cas9) to assess locus dependent performance.

• Expand the Discussion to place Tracking seq2 more clearly within the current landscape of off target detection and prediction tools, and to more thoroughly discuss its advantages and limitations relative to existing methods.

• Consider whether the use of NHEJ inhibitors in "clinically relevant" cells introduces biases in off target spectra (e.g. shift to MMEJ/HR): does Tracking-seq2 truly reflect physiological off target behavior, or a modified repair landscape used only for detection?

• I would suggest comparing Tracking-seq2 against other state of the art assays (e.g. DISCOVER Seq/+, ONE seq, GUIDE seq/2, CHANGE seq, SITE seq) on the same targets and in primary cells (at least another assay respect to the two mentioned here) to provide more robust findings.

• You should mention if you menage to reduce the cell numbers required for Tracking-seq2 respect to other state of the art assays. If so, this is a major technical advance (e.g. "using low input cell numbers") to explicitly mention.

• Are these differences linked to specific variants at off target sites (e.g. SNVs/indels that create or destroy PAMs/mismatches)?

Results section:
1. Mechanistic section (T5 + NHEJ inhibition)
- Strength: They nicely demonstrate that overexpressed T5 exonuclease and NHEJ inhibition (KU 60648) each increase the number and amplitude of RPA ssDNA signals, and that the combination yields ~2–2.5× more Cas9 and ~5× more Cas12a off targets versus the original Tracking seq in HEK293T cells. This directly supports their technical claim of improved sensitivity. The points I would like to raise are:

• overexpression of a bacterial exonuclease and pharmacological NHEJ inhibition substantially perturb the repair landscape. The authors should explicitly discuss to what extent the resulting off target profile reflects a "physiological" spectrum versus a sensitized, detection optimized context, especially for sites with marginal activity.

• is the increased number of sites accompanied by a shift in indel spectra or microhomology usage at on and off targets (i.e. evidence of more MMEJ)? Did you check for increased cytotoxicity or global DNA damage?

• for base and prime editors, only T5 (no KU 60648) is used. It would be helpful if the authors comment on why NHEJ inhibition is not applied there (e.g. not relevant for SSB based pathways, or toxicity/artefacts).

2. Comparison to CIRCLE seq / Cas OFFinder
- Strength: The authors validate >5,000 candidate sites by capture seq and show that the majority of Tracking seq2 only or

Tracking seq2 shared sites have measurable indel frequencies, whereas many CIRCLE seq/Cas OFFinder only sites do not. This supports a good precision/specificity argument for Tracking seq2 in cells. The points I would like to raise are:

• Sensitivity claims are relative and incomplete. They only compare against one in vitro assay (CIRCLE seq) and one in silico tool, not against other modern cell based or in situ methods (GUIDE seq/2, DISCOVER seq/+, GUIDE tag, CHANGE seq, AID seq, etc.), several of which are known to detect high mismatch and non NGG sites. I would suggest softening statements like "high specificity and sensitivity" and especially any "most sensitive" wording, or adding direct benchmarks to at least one other state of the art cellular assay if feasible.

• The conclusion that cell free/computational methods "miss substantial true off target sites" is likely correct in principle, but here it rests on one guide pair and one cell line. I would extend the comparison to additional guides and/or editors.

3. Application to BEs and PEs
- Strength: They demonstrate that T5 also boosts signals for CBE, ABE, and PE3, yielding ~3× more CBE off targets (VEGFA2), ~1.5× for HEK4, while PEs remain relatively high fidelity but gain some additional low frequency sites. This supports the claim that Tracking seq2 generalizes to SSB based editors. The points I would like to raise are:

• Please explicitly report editing frequencies (not just "novel sites") and a clearer definition of what threshold you used to call an off target for base edits versus indels. It is not clear from the methods.

• Given that PE3 is already known to be specific, it would be good if the authors show that T5 does not generate spurious "off targets" in unedited controls for these modalities, similarly to what they did for Cas9.

4. Primary cells and "personalized" heterogeneity
- Strength: They extend Tracking seq2 to RNP edited primary T cells and CD34+ HSPCs and show ~2–3× more sites vs original Tracking seq, with some sites shared and some unique per cell type. That is a clear practical advance and directly relevant to clinical applications. The points I would like to raise are:

• Heterogeneity across cell types (HEK293T vs T vs HSPC) is not the same as inter individual heterogeneity. Here the authors show that a few off targets are unique to T cells or HSPCs and validate them by amplicon sequencing. Later, they link some differential sites to sequence polymorphisms and also note sites with identical sequence but differential activity, implying chromatin or other factors.

Discussion section:
The current Discussion is very short and reads as promotional rather than balanced. It does not sufficiently position Tracking seq2 in the rapidly evolving landscape of off target detection and prediction tools, nor does it clearly articulate the limitations of the present study (limited benchmarking against other cell based assays, absence of HDR/in vivo data, small number of donors, use of T5 and NHEJ inhibition). I recommend substantially expanding and revising this section to:

• compare Tracking seq2 to existing cell based and in vitro assays and to in silico prediction tools;

• moderate claims of 'unprecedented sensitivity' and 'overcoming a major barrier' unless supported by broader head to head data;

• temper the language around 'personalized off target assessment' and clearly distinguish what is demonstrated here (donor/cell type heterogeneity in vitro) from future, clinically oriented applications;

• explicitly acknowledge method specific limitations, including the perturbed repair context and lack of long term/in vivo data."

Version 1:

Reviewer comments:

Reviewer #1

(Remarks to the Author)
The authors have carefully and comprehensively addressed the prior comments. The revisions have improved the clarity, rigor, and overall impact of the work. I appreciate the thoughtful responses and believe the manuscript is now suitable for publication.

Reviewer #2

(Remarks to the Author)
All my comments were addressed and i recommend the manuscript for publication

Reviewer #4

(Remarks to the Author)
Dr. Cong and colleagues developed Tracking-seq2, an advanced technology that integrates 5′→3′ exonuclease treatment and inhibitors of the non-homologous end joining (NHEJ) pathway with the original Tracking-seq. The overall concept is interesting, but the revised manuscript is still not yet ready for publication in Nature Communications. As the authors note in their rebuttal, they have responded to most concerns raised by the four reviewers, including performing:

- Benchmarking against additional state-of-the-art methods

- Investigation of donor-specific heterogeneity

- Evaluation of TadA-derived CBEs

- Assessment of HiFi Cas9

- Additional targeting of new loci

- Comprehensive amplicon sequencing validation

- Increased biological replicates

- Expansion of the Introduction

- Comprehensive discussion of methodology and limitations

- Refinement of the Methods section

Although most reviewer points were addressed, a few important issues included in my first revision remain that I believe should be resolved to bring the study in line with the journal's standards. I list them below; for several I understand the practical reasons for omission and accept them, but I identify one experiment that I think should be added as a minor revision.

1) Biological replicates (increase to n = 5). The authors state they agreed with the handling editor that n = 3 is acceptable for this type of NGS assay. Given the substantial cost, labor, and revision time constraints, I accept this compromise.

2) In vivo repopulation assay to evaluate selection/expansion of edited clones. The authors did not perform such an experiment. They have, however, discussed the limited temporal detection window of Tracking-seq2 as a limitation in the revised Discussion. I accept this justification.

3) Off-target detection in the context of HDR using different donor templates (AAV6 vs. ssODN). The authors did not address this; I acknowledge that this likely requires a dedicated study and accept its omission here.

4) Comparison of CB-derived HSPCs to mobilized peripheral blood (mPB)-derived HSPCs. The authors did not include this comparison and explain that obtaining mPB HSPCs is challenging. They nonetheless provide convincing evidence validating Tracking-seq2's technical sensitivity on CB-derived HSPCs. I accept this limitation.

5) Cytotoxicity and global DNA-damage assessment following T5 or NHEJ inhibitor treatment. The authors did not assess cytotoxicity or global DNA damage in treated cells. I consider this an important omission. I recommend they perform these assays (for example, viability/apoptosis assays and γH2AX or comet assays) at least in n = 3 biological replicates using CB-derived HSPCs. I view this as a reasonable minor revision that would align the manuscript with Nature Communications' quality standards.

Overall recommendation: conditional acceptance after minor revision to include cytotoxicity and global DNA damage assays (point 5). The other omissions are acceptable with the authors' provided explanations and discussion.

Open Access This Peer Review File is licensed under a Creative Commons Attribution 4.0 International License, which permits use, sharing, adaptation, distribution and reproduction in any medium or format, as long as you give appropriate credit to the original author(s) and the source, provide a link to the Creative Commons license, and indicate if changes were made.
In cases where reviewers are anonymous, credit should be given to 'Anonymous Referee' and the source.
The images or other third party material in this Peer Review File are included in the article's Creative Commons license, unless indicated otherwise in a credit line to the material. If material is not included in the article's Creative Commons license and your intended use is not permitted by statutory regulation or exceeds the permitted use, you will need to obtain permission directly from the copyright holder.
To view a copy of this license, visit https://creativecommons.org/licenses/by/4.0/

Summary of Revisions

We sincerely appreciate the handling editor and all reviewers for their careful evaluation, valuable comments, and constructive suggestions. To comprehensively address the reviewers' concerns, we have conducted substantial new experiments in the revised manuscript, including hundreds of independent Tracking-seq/Tracking-seq2 and targeted amplicon sequencing experiments, as well as 12 additional GUIDE-seq-2 assays.

The key revisions are outlined as follows:

1. **Benchmarking against additional state-of-the-art methods:** We added a direct comparison between Tracking-seq2, GUIDE-seq-2, and COSMID in both HEK293T and primary T cells. The results demonstrate that Tracking-seq2 successfully identified true off-target sites that were missed by the other two methods.

2. **Investigation of donor-specific heterogeneity:** We expanded our primary cell cohort by adding three new T cell donors, bringing the total to four distinct donors. This allowed us to confirm the presence of donor-specific differential off-target effects, some of which are highly likely driven by inter-individual genetic diversity.

3. **Evaluation of TadA-derived CBEs:** We introduced new experiments evaluating Td-CBEmax. Tracking-seq2 revealed distinct, non-overlapping off-target profiles between CBE4max and Td-CBEmax. Subsequent amplicon sequencing validated that this editor-specific bias in off-target effects is genuine.

4. **Assessment of HiFi Cas9:** We incorporated Tracking-seq2 profiling for HiFi Cas9, demonstrating that its off-target landscape represents a markedly reduced subset of the sites generated by WT Cas9.

5. **Additional target loci:** To further validate the enhanced sensitivity of Tracking-seq2, we expanded our tested panel by adding two new sgRNAs for Cas9, and one additional sgRNA each for ABE, CBE, and PE3.

6. **Comprehensive amplicon sequencing validation:** We performed hundreds of UMI-based targeted amplicon sequencing assays. These were systematically utilized to: (1) validate novel off-target sites detected by Tracking-seq2, (2) benchmark Tracking-seq2 against GUIDE-seq-2 and COSMID, (3) confirm differential off-target events, and (4) validate off-target sites harboring high mismatches or non-NGG PAM sequences.

7. **Increased replicates:** We substantially increased the number of biological replicates, upgrading the experimental design to n = 3 for the vast majority of Tracking-seq and Tracking-seq2 conditions to ensure robust reproducibility.

8. **Expansion of the Introduction:** We expanded the Introduction section to better contextualize the background and fundamental principles of Tracking-seq, including the standard workflow and the "Track score" concept. Additionally, we incorporated an overview of relevant DNA repair pathways to clearly elucidate the rationale underlying Tracking-seq2.

9. **Comprehensive Discussion of methodology and limitations:** We thoroughly expanded the Discussion section. First, we elaborated on our two-step strategy, outlining the distinct rationale behind the "detection phase" and the subsequent "validation phase". Next, we illustrated the technical advantages of Tracking-seq2 over different types of existing methods. Finally, we explicitly detailed the limitations of Tracking-seq2 and this study, including: the inability to directly capture large structural variations, the potential risk of overlooking late-occurring off-target events due to early sampling timepoints, the suboptimal sensitivity for weak off-target effects induced by CBEs, and the current lack of *in vivo* profiling evaluations.

10. **Refinement of the Methods section:** We provided more details in the Methods section, expanding the descriptions of various experimental procedures (e.g., cell electroporation conditions) and providing a more detailed pipeline of our bioinformatic analysis.

We believe these comprehensive revisions and the substantially expanded datasets have markedly strengthened the manuscript, demonstrating the value of Tracking-seq2 for off-target detection of genome editing.

Table of Contents

Summary of Revisions 1

Reviewer #1 3

Reviewer #2 15

Reviewer #3 25

Reviewer #4 28

Point-by-point responses to the reviewers' comments

Reviewer #1

(Remarks to the Author):
Summary and Overall Assessment
In this manuscript, the authors present Tracking-seq2, which they describe as an improved, broadly applicable platform for genome-wide detection of off-target editing events. While the topic is timely and relevant to the genome-editing community, this reviewer finds that the study does not provide sufficient benchmarking or methodological rigor to justify the central claims of superiority over existing tools. The comparison set is also highly problematic: Cas-OFFinder and CIRCLE-Seq are well documented to yield high rates of false positives relative to more high-fidelity approaches such as GUIDE-Seq and COSMID. As a result, the work appears to offer only incremental improvements over both these existing methods and the authors' own Tracking-seq1 system, and does not substantively address key emerging concerns in the field such as detection of large genomic abnormalities.

Reply:
Thank you very much for your critical feedback regarding the benchmarking strategy. We agree that rigorous comparison with high-fidelity cell-based methods is essential to validate the utility of Tracking-seq2. To address this concern, we have performed additional comprehensive benchmarking experiments directly comparing Tracking-seq2 against two state-of-the-art methods, GUIDE-seq-2 and COSMID.

In HEK293T cells: Tracking-seq2 exhibited superior sensitivity, identifying a significantly larger number of validated off-target sites compared to GUIDE-seq-2 and COSMID.

In primary T cells, Tracking-seq2 demonstrated sensitivity comparable to, or higher than, GUIDE-seq-2 and COSMID. Crucially, Tracking-seq2 identified unique, validated off-target sites missed by the other methods, indicating that our method offers complementary value for comprehensive safety assessment. These new data confirm that Tracking-seq2 provides robust sensitivity that meets or exceeds current high-fidelity standards.

Regarding the detection of large structural variants (SVs), we acknowledge this as an important emerging concern. While Tracking-seq2 effectively identifies the off-target cleavage sites that potentially initiate SVs, it does not directly provide any SV information. Our current validation method (targeted amplicon sequencing or hybrid capture sequencing) is limited to detecting small indels. We have clearly discussed this limitation in the revised manuscript.

Major Comments
1. Conceptual framing of "improvement."
The authors assume that identifying a greater number of putative off-target sites represents an intrinsic improvement in detection capability. However, in practice, the vast majority of such sites reported by methods including CIRCLE-Seq, SITE-Seq, and GUIDE-Seq are false positives that do not validate by targeted deep sequencing in clinically relevant systems (e.g., transient delivery of Cas9, base editor, or mRNA RNPs to human primary HSPCs). Given that a single SNP within a

protospacer can ablate editing, it is implausible that sites with up to seven mismatches (Fig. 2) represent true off-target events.

Furthermore, the finding that "most Tracking-seq2-only sites contained non-NGG PAMs" is concerning, as Cas9 activity is well-established to be PAM-dependent. The authors should reconsider this premise and provide supporting validation data beyond the brief testing of Tracking-seq2 vs. Tracking-seq1 that is done in primary T cells and HSPCs.

Reply:

Thank you for the insightful comments regarding the credibility of off-target sites with high mismatches or non-NGG PAMs. We apologize that our initial Figure 2 contained an error in the sequence alignment, in which DNA bulges were not properly displayed and led to incorrect number of mismatches in some cases. After fixing this error, we found 3 sites with 7 mismatches that were only detected by Tracking-seq2 in HEK293T cells, and there were relatively more sites with 6 mismatches and non-NGG PAM.

While such events are rare, their occurrence is consistent with recent work showing that DNA topology (e.g., negative supercoiling) can lower the energetic barrier for off-target cleavage and increase tolerance to mismatches across the protospacer (Smith, Q. M. *et al.*). Moreover, although the canonical NGG PAM is preferred, off-target editing at non-canonical PAMs is supported by previous literature (Zhang, Y. *et al.*; Lazzarotto, C. R. *et al.*). Specifically, we detected 5 types of non-canonical PAMs (NAG, NCG, NTG, NGA, and NGC) at off-target sites. For each type, we performed amplicon sequencing on two representative off-target sites for further verification. The amplicon results **(Fig. R1a)** and the distinct Tracking-seq2 signals at the off-target sites **(Fig. R1b)** confirmed that off-target cleavage could occur with these 5 non-canonical PAMs. Besides, we performed amplicon sequencing on the 3 off-target sites with 7 mismatches, as well as 7 sites with 6 mismatches. Most of them were verified as true off-target events **(Fig. R1c-d)**. Taken together, these data demonstrate off-target editing can occur at sites with up to 7 mismatches and non-canonical PAMs, indicating the unique capability of Tracking-seq2 to uncover atypical off-target sites.

Reference:

Smith, Q. M. *et al.* Structural basis of supercoiling-induced CRISPR-Cas9 off-target activity. *Nature* Advance online publication. (2026). https://doi.org:10.1038/s41586-026-10255-7

Zhang, Y. *et al.* Comparison of non-canonical PAMs for CRISPR/Cas9-mediated DNA cleavage in human cells. *Sci Rep* **4**, 5405 (2014). https://doi.org:10.1038/srep05405

Lazzarotto, C. R. *et al.* CHANGE-seq reveals genetic and epigenetic effects on CRISPR-Cas9 genome-wide activity. *Nat Biotechnol* **38**, 1317-1327 (2020). https://doi.org:10.1038/s41587-020-0555-7

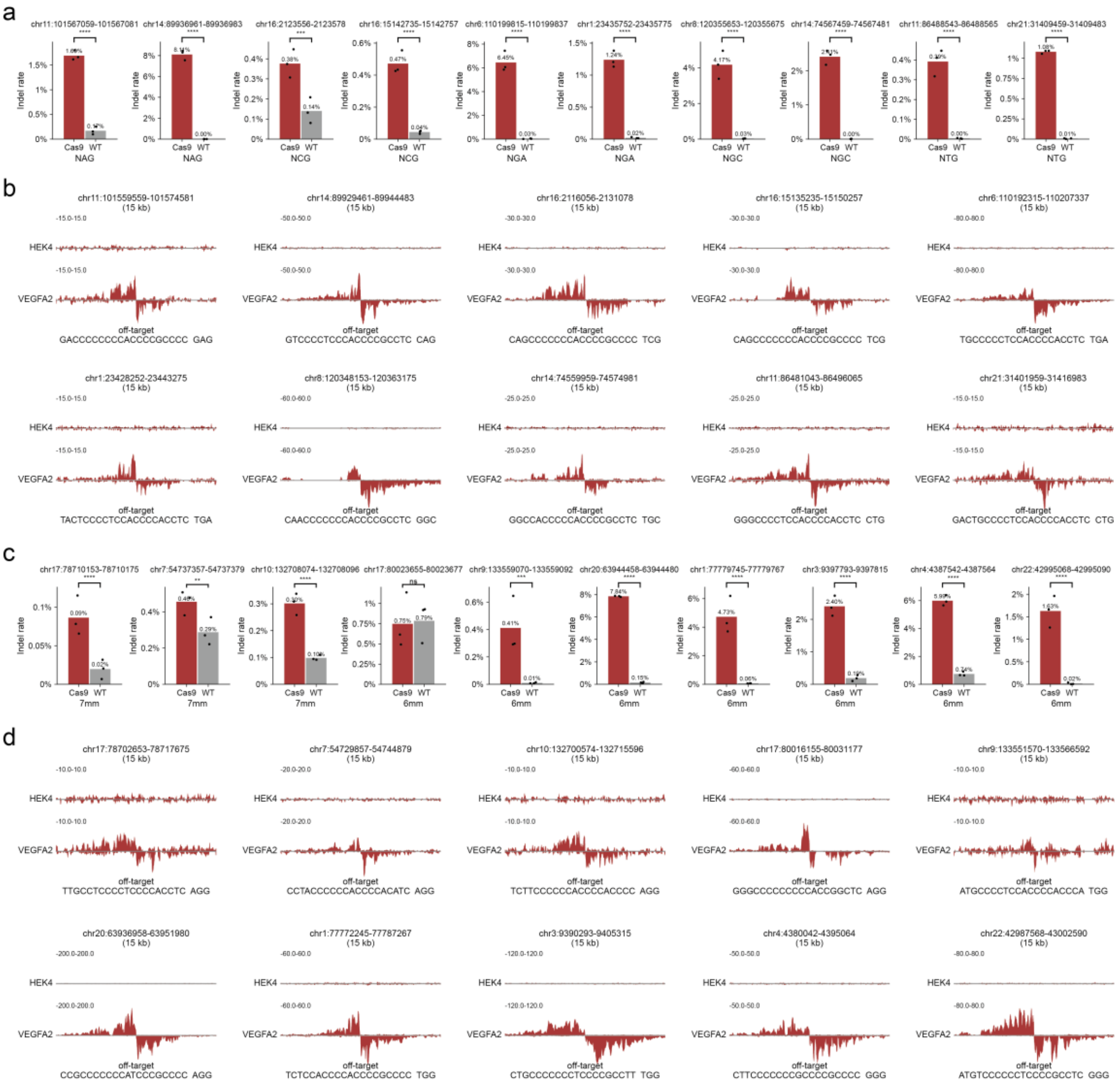

Fig. R1 (Corresponding to Extended Data Fig. 5 in the revised manuscript). Validation of off-target sites with non-canonical PAMs and high mismatches detected by Tracking-seq2. a, Targeted amplicon sequencing validation of off-target sites with non-canonical PAMs detected by Tracking-seq2 for Cas9 + sgRNA VEGFA2 in HEK293T cells. Five non-canonical PAMs (NAG, NCG, NTG, NGA, and NGC) were identified, and two representative off-target sites for each type were validated. All tested sites exhibited bona fide off-target editing. *P*-values were calculated by one-sided binomial tests and the exact value for each site is listed in Supplementary Table 2. **b**, Tracking-seq2 signals at representative off-target sites with non-canonical PAMs for Cas9 + sgRNA VEGFA2 in HEK293T cells. Distinct signals confirmed off-target cleavage with all five non-canonical PAM types. Cas9 + sgRNA HEK4 served as a non-targeting control. **c**, Targeted amplicon sequencing validation of off-target sites with 6 or 7 mismatches detected by Tracking-seq2 for Cas9 + sgRNA VEGFA2 in HEK293T cells. 9 out of 10 were verified as true off-target events. *P*-values were calculated by one-sided binomial tests and the exact value for each site is listed in Supplementary Table 2. **d**, Tracking-seq2 signals at representative off-target sites with 6 or 7 mismatches for Cas9 + sgRNA VEGFA2 in HEK293T cells. Cas9 + sgRNA HEK4 served as a non-targeting control.

2. Insufficient methodological detail.

Key experimental details are omitted or deferred to the Methods, impeding reader understanding. For example, Figures 1–2 do not specify the cell type used or whether experiments were cell-based or cell-free. Figure 1c is described only as showing "signals," without defining what is plotted. Similarly, the term "Track score" (Fig. 1g) is introduced without explanation. The manuscript would benefit from clearer figure legends and concise descriptions of experimental design throughout. In addition, nowhere are adequate details provided on the experiments in primary cells. For instance, what nucleofection system, buffer, and code was used for these experiments? Was the Cas9 high-fidelity which is now routinely included in genome editing trials as opposed to WT which has much higher off-target editing?

Reply:

We apologize for the lack of methodological clarity in the initial submission. We have extensively revised the figure legends and Methods section to provide the necessary details. Specifically, we have clarified that Tracking-seq and Tracking-seq2 are cell-based methods and the experiments in Figures 1–2 were performed in HEK293T cells. Regarding the primary cell experiments, we have added detailed protocols specifying the nucleofection system, buffers, and data analysis used for T cells and HSPCs. In addition, we have added a new Methods section of Tracking-seq/Tracking-seq2 data analysis and provided a description of Tracking-seq workflow and the concept of "Track score" in the Introduction section as follows (Lines #57-63):

"The workflow comprises four major steps: (1) capturing RPA-bound ssDNA using CUT&RUN; (2) removing double-stranded DNA (dsDNA) using HL-dsDNase; (3) performing strand-specific ssDNA library construction; and (4) next-generation sequencing (NGS) coupled with Offtracker bioinformatics analysis. The Offtracker software calculates a normalized "Track score" for each candidate off-target site, which represents the intensity of Tracking-seq signal, and the empirical threshold for a positive site is Track score > 2."

We have added clearer figure legends to provide a better understanding of each panel. As representative examples, please refer to the updated descriptions for the panels regarding 'signal' and 'Track score' shown below in **Fig. R2a-b**.

To investigate high-fidelity Cas9, we performed additional experiments comparing WT SpCas9 and HiFi Cas9 in HEK293T cells. As expected, HiFi Cas9 showed substantially fewer off-target events compared to WT Cas9 **(Fig. R2c-j)**. These results align with the consensus in the field and reinforce that HiFi Cas9 is indeed more suitable for clinical applications.

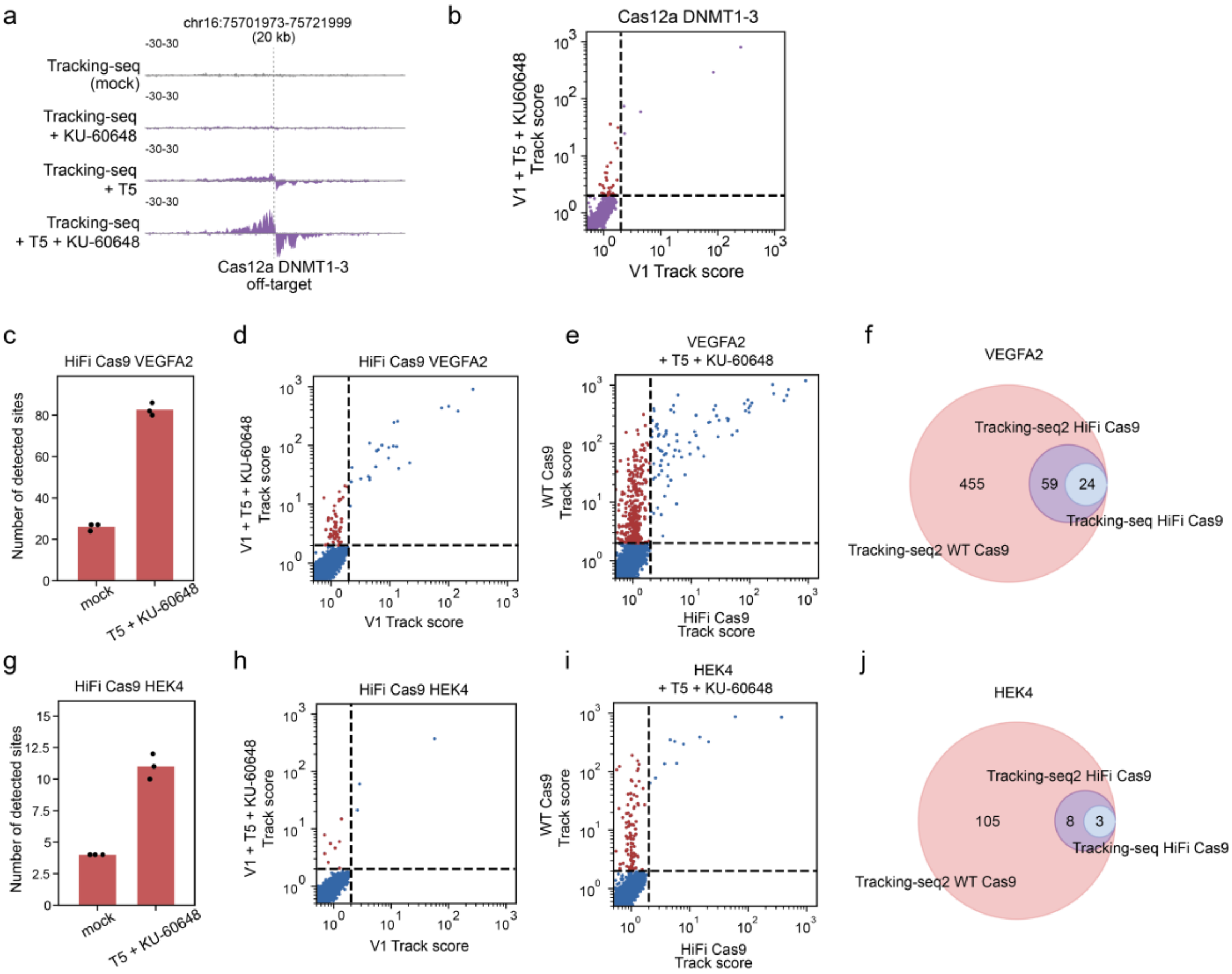

Fig. R2 (Corresponding to Fig. 1j-k and Extended Data Fig. 4i-p in the revised manuscript). a, Strand-specific signals generated by Tracking-seq at representative off-target sites caused by Cas12a + sgRNA DNMT1-3 in various conditions. The range in the top left of each track denotes normalized signal intensity, represented as read coverage per 10 million mapped reads. **b,** Scatter plot showing the Track scores of Tracking-seq with combined T5 co-transfection and KU-60648 treatment versus those without treatment edited by Cas12a + sgRNA DNMT1-3. Each dot represents a candidate off-target site. Dashed lines represent the thresholds of the Track score > 2 for positive sites. V1: Tracking-seq1. **c**, Numbers of off-target sites caused by HiFi Cas9 + sgRNA VEGFA2 detected by Tracking-seq in mock group and combined treatment of T5 and KU-60648 group. Each dot represents an independent biological replicate (n = 3). **d**, Scatter plot showing the Track scores of Tracking-seq with combined T5 co-transfection and KU-60648 treatment versus those without treatment edited by HiFi Cas9 + sgRNA VEGFA2. Each dot represents a candidate off-target site. Dashed lines represent the thresholds of the Track score > 2 for positive sites. **e**, Scatter plots comparing the Track score of off-target sites caused by Cas9 and those caused by HiFi Cas9, in HEK293T cells (VEGFA2). **f**, Venn diagram comparing off-target sites generated by Cas9 or HiFi Cas9 + VEGFA2, detected by Tracking-seq without treatment (mock) or by Tracking-seq2 (combined treatment of T5 and KU-60648). **g**, Numbers of off-target sites caused by HiFi Cas9 + sgRNA HEK4 detected by Tracking-seq in mock group and combined treatment of T5 and KU-60648 group. Each dot represents an independent biological replicate (n = 3). **h**, Scatter plot showing the Track scores of Tracking-seq with combined T5 co-transfection and KU-60648 treatment versus those without treatment edited by HiFi Cas9 + sgRNA HEK4. Each dot represents a candidate off-target site. Dashed lines represent the thresholds of the Track score > 2 for positive sites. **i**, Scatter

plots comparing the Track scores of off-target sites caused by Cas9 and those caused by HiFi Cas9, in HEK293T cells (HEK4). **j**, Venn diagram comparing off-target sites generated by Cas9 or HiFi Cas9 + sgRNA HEK4, detected by Tracking-seq without treatment (mock) or by Tracking-seq2 (combined treatment of T5 and KU-60648).

3. Benchmarking against unreliable comparators.

The authors benchmark Tracking-seq2 primarily against CIRCLE-Seq and Cas-OFFinder, both of which have been shown to exhibit low positive predictive value in clinically relevant contexts (Cromer et al., Mol Ther 2023). More meaningful benchmarking would include empirical, cell-based assays such as GUIDE-Seq and computational tools like COSMID, which more effectively balance sensitivity with specificity. Without these essential comparisons—particularly in human primary cells—to demonstrate that Tracking-seq2 identifies bona fide off-target sites that would be missed by these methods, the authors' claims of superior performance are not supported. Clearly demonstrating how the reported modifications enhance sensitivity, reproducibility, or practical applicability is essential to justify publication in Nature Communications.

Reply:

Thank you for pointing out the limitations of our previous benchmarking. We agree that comparisons with reliable cell-based methods are necessary. In the revised manuscript, we have added direct comparisons with GUIDE-seq-2 and COSMID in HEK293T cells and primary T cells.

In HEK293T cells, Tracking-seq2 demonstrated superior sensitivity. Specifically, for both HEK4 and VEGFA2 targets, Tracking-seq2 identified a substantial number of additional off-target sites while capturing the vast majority of sites detected by GUIDE-seq-2 **(Fig. R3a)**. We conducted targeted amplicon sequencing for novel off-target sites that were not included in our hybrid capture data (i.e. unique novel sites from GUIDE-seq-2 or COSMID) and analyzed the distribution of Δindel rates (indel rates of Cas9-edited samples minus indel rates of WT samples) among different subsets of sites **(Fig. R3b)**. This result confirmed that Tracking-seq2 detected a lot of bona fide off-target sites missed by GUIDE-seq-2 or COSMID, while these two methods also provided several true off-target sites missed by Tracking-seq2.

In primary T cells, three methods shared common off-target sites and also included unique sites **(Fig. R3c).** To validate these unique sites, we performed amplicon sequencing on the top-10 unique sites specific to each method. The results showed that Tracking-seq2-only sites had higher Δindel rates than the others, while the other two methods also identified several genuine editing sites **(Fig. R3d)**.

According to these results, we recommend combining these technologies in clinical applications to ensure the most comprehensive safety assessment.

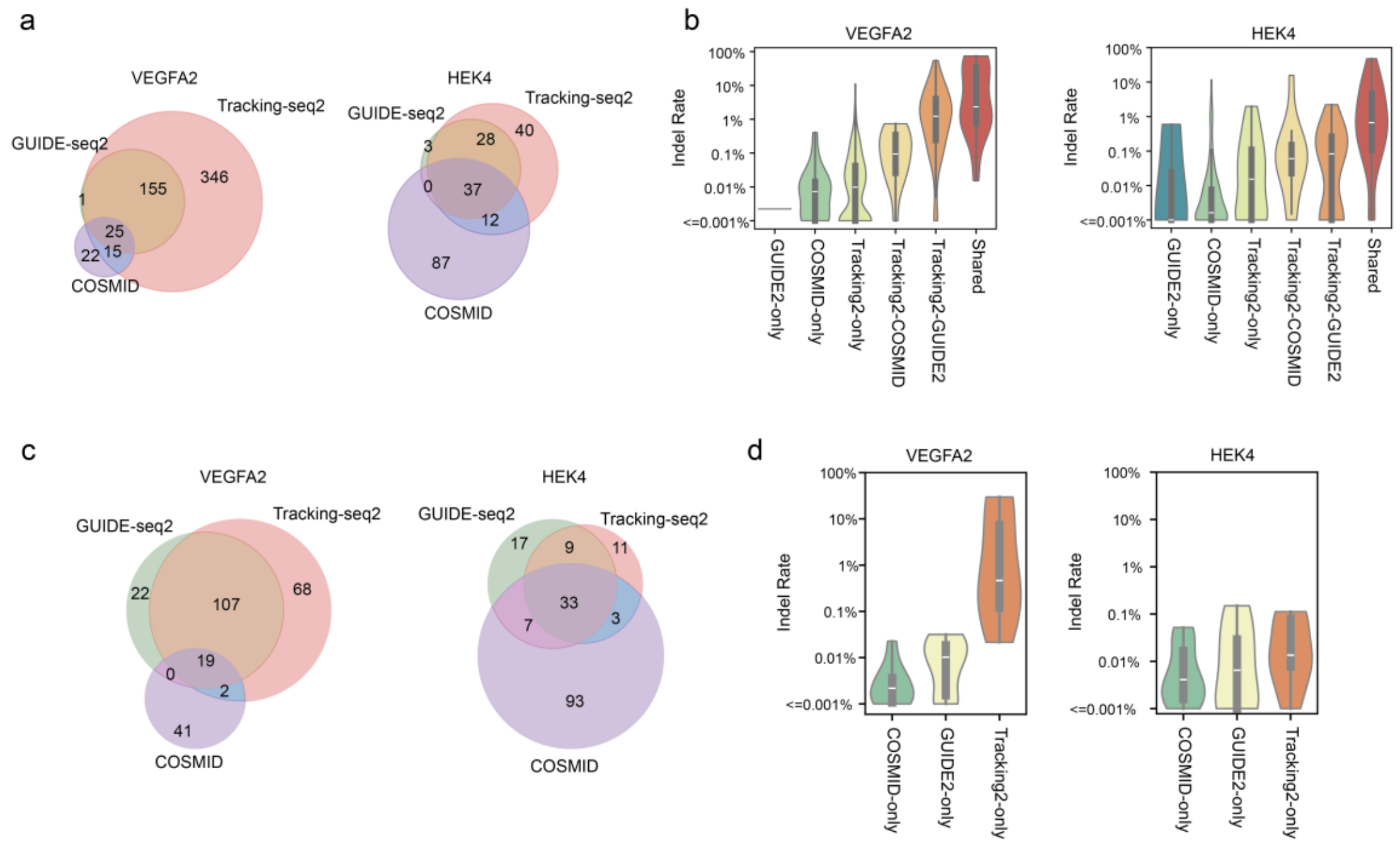

Fig. R3 (Corresponding to Fig. 2c-d and Fig. 4e-f in the revised manuscript). a, Venn diagrams comparing off-target sites detected (or predicted) by Tracking-seq2, GUIDE-seq-2, and COSMID for Cas9 + sgRNA VEGFA2 or Cas9 + sgRNA HEK4. **b**, Box plot showing the distribution of Δindel rates measured by hybrid capture targeted sequencing at off-target sites belonging to different sets for Cas9 + VEGFA2 and Cas9 + HEK4. GUIDE-seq-2 and COSMID missed a considerable number of off-target sites detected by Tracking-seq2. **c**, Venn diagrams comparing off-target sites detected (or predicted) by Tracking-seq2, GUIDE-seq-2, and COSMID for Cas9 + VEGFA2 or Cas9 + HEK4 in human T cells. **d**, Box plot showing the distribution of Δindel rates measured by targeted amplicon sequencing at method-specific off-target sites (top10 per method) for Cas9 + VEGFA2 or Cas9 + HEK4 in human T cells. Off-target sites exclusively identified by Tracking-seq2 exhibited the highest indel frequencies compared to those uniquely detected by GUIDE-seq-2 or COSMID.

4. Dependence on prior Tracking-seq knowledge.

The manuscript presumes substantial familiarity with the authors' previous Tracking-seq1 work, providing insufficient detail on how Tracking-seq2 differs. This limits accessibility to new readers and weakens the authors' case for innovation. A clear summary of methodological differences and key optimizations should be added to the Introduction or early Results section.

Reply:

Thank you for pointing out the lack of background information. To improve the accessibility of the manuscript for a broader readership, we have extensively revised the introduction section. We now provide a comprehensive overview of the original Tracking-seq methodology, clearly defining the mechanistic basis of RPA-bound ssDNA tracking. Following this, we explicitly detail the specific optimizations introduced in Tracking-seq2, including the rationale and implementation of 5′→3′ exonuclease treatment and NHEJ inhibition. Furthermore, we have updated Fig. 1a and Extended Data Fig. 1 to visually summarize these methodological differences **(Fig. R4)**, providing a clear comparison of the workflows and highlighting the key steps where Tracking-seq2 diverges to achieve enhanced sensitivity.

[Figure Redacted]

Fig. R4 (Corresponding to Fig. 1a and Extended Data Fig. 1 in the revised manuscript). a, Schematic illustration of the Tracking-seq2 workflow for Cas9 and Cas12a off-target detection. Exogenous T5 exonuclease treatment and NHEJ inhibition (using KU-60648) synergistically enhance the detection of Cas9 and Cas12a off-target sites by increasing the accumulation of RPA-bound ssDNA intermediates. The Tracking-seq2 workflow consists of two distinct phases: (1) Detection Phase—cells treated with T5 exonuclease and NHEJ inhibitor are used for the Tracking-seq2 assay to enrich ssDNA intermediates and maximize detection sensitivity; (2) Validation Phase—cells edited without T5 exonuclease and NHEJ inhibitor are used for targeted deep sequencing to measure actual off-target efficiency under physiological conditions. **b**, Schematic diagram illustrating the Tracking-seq2 strategy for modulating DNA repair pathway s to enhance off-target detection. The approach incorporates two key interventions: (1) pharmacological inhibition of the NHEJ pathway using KU-60648 to favor homologous recombination (HR), thereby increasing the accumulation of RPA-bound single-stranded DNA (ssDNA) intermediates; and (2) application of exogenous T5 exonuclease (a 5′→3′ exonuclease) to process DNA ends and further amplify RPA-bound ssDNA signals. **c**, Schematic illustration of the Tracking-seq2 workflow for BEs and PEs. Exogenous T5 exonuclease enhances the off-target detection sensitivity for BEs and PEs. Note that NHEJ inhibition is not applied for BEs and PEs, as these editors generate SSBs rather than DSBs, and NHEJ is primarily involved in DSBs repair.

5. Limit of detection.

The reported indel frequency of 0.005% (Fig. 1c) appears below reasonable detection thresholds. The authors should define and experimentally establish the assay’s limit of detection and dynamic range.

Reply:

Thank you for pointing out the importance of defining the detection limit of hybrid capture sequencing. To evaluate the reliability of hybrid capture data within different ranges of Δindel rates, we performed targeted amplicon sequencing with unique molecular identifiers (UMIs) for off-target sites stratified by editing efficiency (10 sites per tier): 1–10%, 0.1–1%, 0.01–0.1%, and 0.001–0.01% Δindel rates **(Fig. R5a-d)**. The results confirmed genuine editing at all 20 tested sites with 0.1%–10% Δindel rates, and at half of the sites within the 0.001%–0.1% range **(Fig. R5e)**. The amplicon sequencing experiments were performed with edited cells 120 hours post-transfection, therefore true off-target sites exhibited similar or higher Δindel rates as expected **(Fig. R5f)**. According to this result, approximately half of the Tracking-seq2-only sites were true off-target sites under the physiological editing condition, and sites co-identified by Tracking-seq2 and either GUIDE-seq-2 or COSMID exhibited greater reliability **(Fig. R5g-h)**.

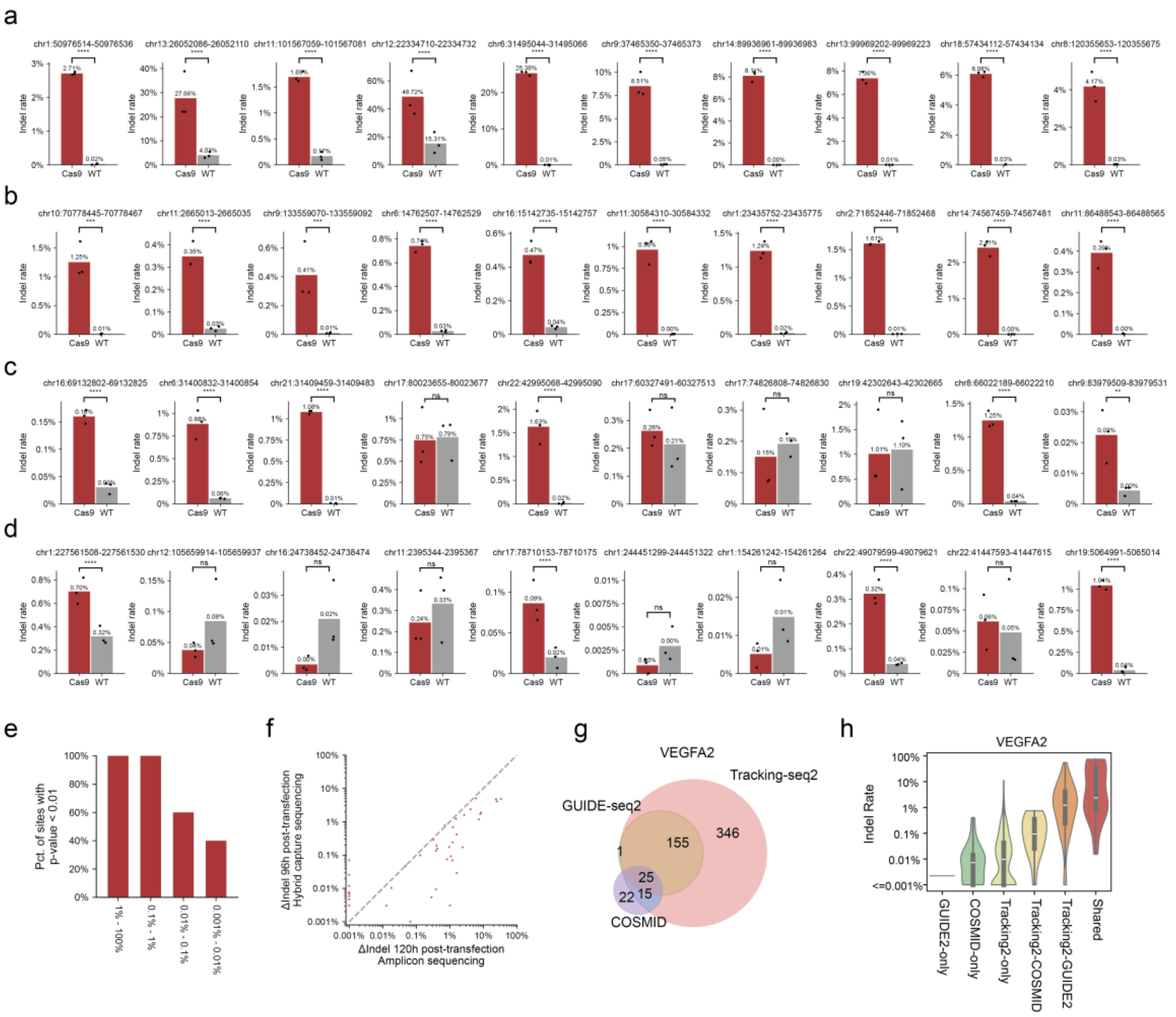

Fig. R5 (Corresponding to Extended Data Fig. 3 and Fig. 2c-d in the revised manuscript). a-d, Bar plots showed the targeted amplicon sequencing validation (120 h post-transfection) of off-target sites with 1–10% (a), 0.1–1% (b), 0.01–0.1% (c), and 0.001–0.01% (d) Δindel rates detected by hybrid capture sequencing (96 h post-transfection). *P*-values were calculated by one-sided binomial tests and the exact values site are listed in Supplementary Table 2. **e**, Bar plot displays the

percentage of sites—within each Δindel rate bin previously determined by hybrid capture sequencing—that exhibited statistically significant editing (p-value < 0.01 versus WT control) in the targeted amplicon validation. **f**, Scatter plot comparing off-target Δindel rates measured by hybrid capture sequencing at 96 h post-editing versus targeted amplicon sequencing at 120 h post-editing. True off-target sites exhibited similar or higher Δindel rates as expected. **g**, Venn diagrams comparing off-target sites detected (or predicted) by Tracking-seq2, GUIDE-seq-2, and COSMID for Cas9 + sgRNA VEGFA2 or Cas9 + sgRNA HEK4. **h**, Box plot showing the distribution of Δindel rates measured by hybrid capture targeted sequencing at off-target sites belonging to different sets for Cas9 + VEGFA2 and Cas9 + HEK4. GUIDE-seq-2 and COSMID missed a considerable number of off-target sites detected by Tracking-seq2.

6. Scope limited to small indels.

The study focuses exclusively on small indels and does not address large genomic alterations increasingly recognized as clinically relevant consequences of genome editing (e.g., large on-target deletions, chromosome arm loss). The fact that this is neither acknowledged in this paper is a major weakness.

Reply:

Thank you for highlighting this limitation. We agree that large genomic alterations are a critical safety concern. Tracking-seq2 is designed to detect genome-wide off-target cleavage sites. Theoretically, these cleavage sites are the potential origin of large structural variations (SVs). However, Tracking-seq2 does not directly provide information on SVs and our current validation method (targeted amplicon sequencing or hybrid capture sequencing) is limited to detecting small indels. We acknowledge this as a limitation of the current study and added this in the Discussion.

7. Base and prime editing data interpretation.

The reported discrepancy between hundreds of off-target sites for Cas9 and only tens for base or prime editors is not adequately explained. Is this due to intrinsic differences in editing mechanisms or reduced assay sensitivity for single-strand or nicking systems? Additional clarification is warranted.

Reply:

Thank you for the question. As we reported in our previous Tracking-seq1 paper, different genome editing tools targeting the same locus exhibit heterogeneous off-target profiles. In our previous Tracking-seq1 study, we also found that Tracking-seq1 detected fewer off-target sites with BEs and PE compared to Cas9 **(Fig. R6a)**. Targeted amplicon sequencing on representative differential off-target sites confirmed that most off-target editing events were editor-specific **(Fig. R6b-f),** with the exception of a few weak CBE-induced off-target events missed by Tracking-seq1. This indicates that the observed discrepancy is largely due to intrinsic differences in editing mechanisms, as nicking systems inherently produce fewer off-targets.

[Figure Redacted]

Fig. R6 (from our previous study (Zhu, M. *et al.*)). a, Scatter plots comparing the off-target sites caused by Cas9 and those caused by CBE, ABE or PEs, respectively, in HEK293T cells (HEK293_site_4). Each dot represents a candidate editing site. **b-f,** Bar chart showing the editing efficiency of 10 tested CBE-biased off-target sites (**b**, dark blue dots in **a**), five tested Cas9-biased off-target sites (**c**, red dots in **a**), seven tested ABE-biased off target sites (**d**, yellow dots in **a**), five tested Cas9-biased off-target sites (**e**, pink dots in **a**) and five tested Cas9-biased off-target sites (**f**, purple dots in **a**). Dots in the bar charts indicate biological replicates.

Reference:

Zhu, M. *et al.* Tracking-seq reveals the heterogeneity of off-target effects in CRISPR-Cas9-mediated genome editing. *Nature Biotechnology* **43**, 799–810. (2024). https://doi.org:10.1038/s41587-024-02307-y

Minor Comments

1. Please remove overly definitive statements such as:

"Tracking-seq2 represents a significant advancement, establishing itself as the most sensitive, cell-based, and clinically applicable off-target detection technology to date."

"This enhancement significantly elevates the precision and reliability of safety evaluations in clinical genome editing."

Such claims are subjective and currently unsupported by comparative data.

Reply:

Thank you for pointing out the inappropriate tone of these statements. We agree that these claims were subjective and not fully supported by the initial data. We have removed these overstated descriptions from the manuscript.

2. Extended Data figure citations appear out of order (e.g., Extended Data Fig. 1l–o cited before 1j–k). Please correct the numbering and sequence of references throughout.

Reply:

We apologize for the confusion caused by the disordered figure citations. We have carefully checked and corrected the numbering and sequence of all references to Figures throughout the manuscript, ensuring they now appear in the correct logical order.

Overall Recommendation

While the manuscript describes a modification to an existing off-target detection platform, the current version lacks the benchmarking, methodological clarity, and novelty necessary to substantiate the authors' claims. To be considered a meaningful advance, Tracking-seq2 must be evaluated using more rigorous clinically relevant genome-editing workflows in human primary cells and directly demonstrate that it identifies bona fide off-target events missed by leading systems such as GUIDE-Seq and COSMID. Without such evidence, the work does not represent a substantive improvement over established approaches and is not suitable for publication in Nature Communications.

Reply:

We sincerely thank the reviewer for the overall assessment and constructive criticism. We fully agree that rigorous benchmarking in clinically relevant contexts is essential. In response, we have extensively revised the manuscript to include direct comparisons with GUIDE-seq-2 and COSMID in both HEK293T cells and primary T cells, as detailed in our responses to specific points above. These new data demonstrate that Tracking-seq2 offers superior or comparable sensitivity and identifies bona fide off-target sites missed by other methods. We believe these additions substantiate the value of Tracking-seq2 as a meaningful advance for publication.

Reviewer #2

(Remarks to the Author):

Safe clinical translation of CRISPR-based therapeutics demands highly sensitive detection of off-target events directly within human cells. The manuscript titled “Ultra-sensitive profiling of CRISPR-Cas off-target effects with Tracking-seq2” describes an enhanced version of the Tracking-seq platform optimized for diverse CRISPR–Cas systems. Tracking-seq2 integrates T5 exonuclease treatment with inhibition of NHEJ DNA repair, thereby improving single-stranded DNA signal detection through RPA pull-down. According to the authors, these additional steps enhance sensitivity up to fivefold while maintaining high specificity. Overall, the work presents a substantial technical advancement in off-target detection;

Reply:

Thank you very much for the accurate summary of our work and for recognizing the importance of sensitive off-target detection for the safe clinical translation of CRISPR-based therapeutics. We are encouraged by your acknowledgement of our strategies, and we have addressed the specific points raised in the following comments.

however, it may be suitable for publication after the authors adequately address the following concerns and limitations:

1. The combined use of DNA repair inhibitors and exonuclease treatment is likely to perturb DNA repair kinetics. Figures 1G, 1K, 3B, and 4B (comparing T5-KU60648 track score vs track score mock) indicate high false discovery rate. The authors should quantify how many additional authentic off-targets (track score mock) Tracking-seq2 identifies when benchmarked against the original Tracking-seq1.

Reply:

Thank you for this concern. We sincerely apologize for the lack of clarity regarding certain potentially misleading terms. In the revised manuscript, we have explicitly clarified the exact meanings of 'mock' and 'Track score'.

Samples co-transfected with the Cas9/sgRNA-expressing PX458 plasmid and a plasmid encoding a specific exonuclease (or treated with drugs like KU-60648) served as the experimental groups, while samples co-transfected with the PX458 plasmid and an empty vector (or without adding drugs) served as control groups named “mock”. The “mock” samples represent the results of Tracking-seq1.

“Track score” is an algorithmic score calculated by the Offtracker software, reflecting the signal intensity of RPA-ssDNA enrichment for each candidate off-target site. The empirical threshold for a positive site is Track score > 2. The term “track score mock” represents the Track scores from Tracking-seq1, and “T5-KU60648 track score” represents the Track scores from Tracking-seq2 (adding the T5 and KU-60648).

We apologize for these misleading terms, and we renamed them as “V1 Track score”, “V1 + T5 Track score”, “V1 + KU-60648 Track score”, “V1 + T5 + KU-60648 Track score” to indicate the conditions more clearly. We also revised the figure legends to enhance the clarity of the article.

For example, **Fig. R7a** compared the Track scores between Tracking-seq1 and Tracking-seq2. Each dot indicates a candidate off-target site. The dashed line indicates the threshold of Track score

for calling a positive off-target site (Track score > 2). The red dots indicate off-target sites exclusively detected by Tracking-seq2.

To validate the additional off-target sites detected by Tracking-seq2 for Cas9 editing in HEK293T cells. We performed hybrid capture sequencing for all detected sites and the results showed that approximately half of the newly detected sites by Tracking-seq2 exhibited Δindel rates > 0.01% **(Fig. R7b)**.

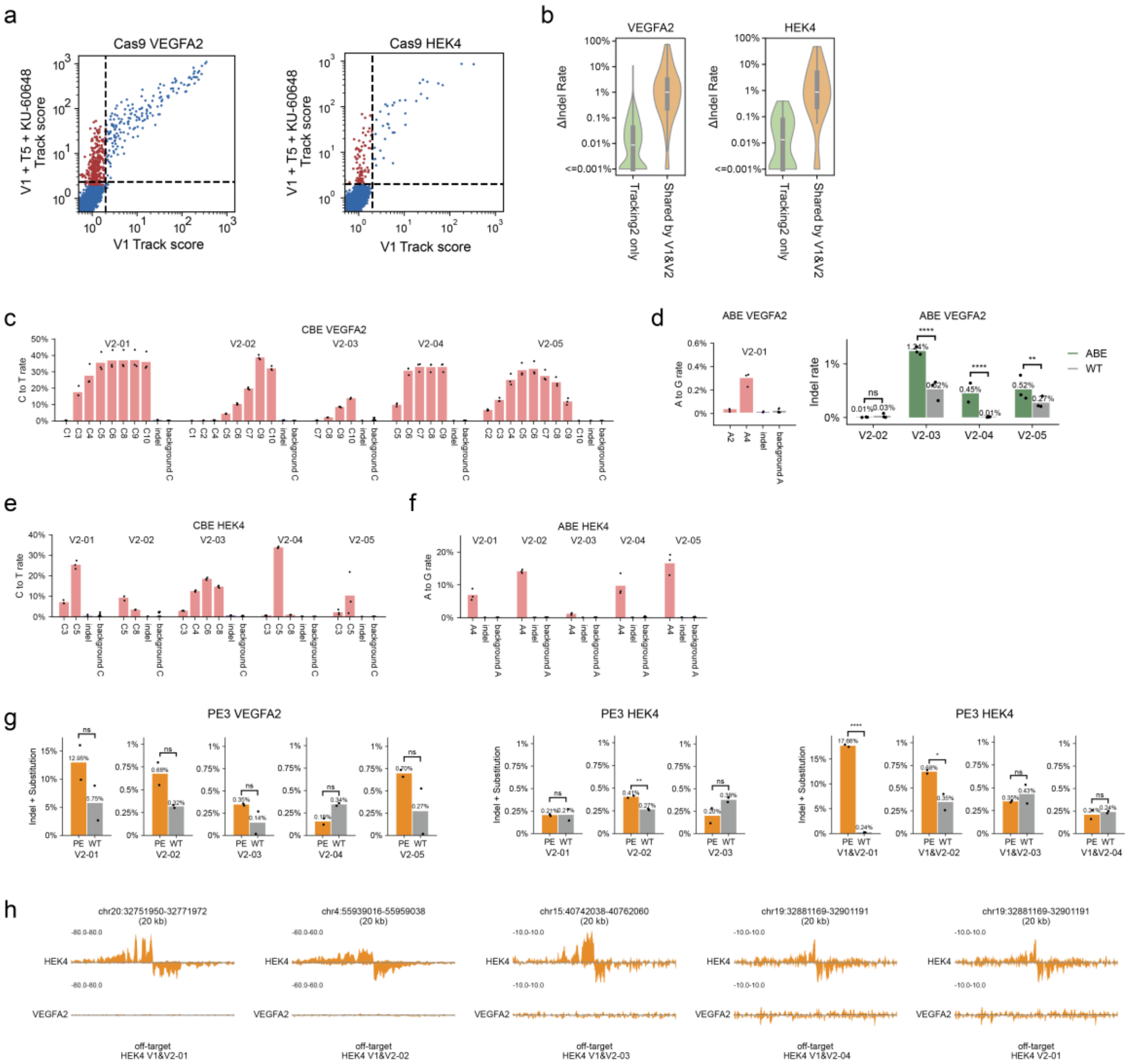

Fig. R7 (Corresponding to Fig. 1g, Extended Data Fig. 2h, 2j, and Extended Data Fig. 7 in the revised manuscript). a, Scatter plot showing the Track scores of Tracking-seq with combined T5 co-transfection and KU-60648 treatment versus those without treatment edited by Cas9 + sgRNA VEGFA2 or HEK4. Each dot represents a candidate off-target site. Dashed lines represent the thresholds of the Track score > 2 for positive sites. V1: Tracking-seq1. **b**, Box plot showing the distribution of indel rates measured by hybrid capture targeted sequencing at off-target sites exclusively detected by Tracking-seq2 and shared by Tracking-seq and Tracking-seq2 for sgRNA VEGFA2 or sgRNA HEK4. Approximately half of the additional off-target sites detected by Tracking-seq2 were validated as true off-target sites in the physiological condition (without T5 or KU-60648 treatment). **c-f**, Targeted amplicon sequencing validation of the top five novel off-target sites detected by Tracking-seq2 for CBE + sgRNA VEGFA2 (a), ABE + sgRNA VEGFA2 (b), CBE + sgRNA HEK4 (c), and ABE + sgRNA HEK4 (d) in HEK293T cells. All tested sites exhibited C-

to-T editing within the editing window for CBE + sgRNA VEGFA2 or HEK4. For ABE + sgRNA VEGFA2 or HEK4, all tested sites with adenines within the editing window exhibited A-to-G editing, while 3/4 sites lacking adenines in the editing window exhibited low-frequency indels. *P*-values were calculated by one-sided binomial tests and the exact value for each site is listed in Supplementary Table 3. Background C/A: C or A outside the editing window. **g**, Targeted amplicon sequencing validation of off-target sites for PE3. Novel off-target sites for PE3 + pegRNA VEGFA2 (left) and for PE3 + pegRNA HEK4 (middle) and four sites jointly detected by both Tracking-seq V1 and V2 for PE3 + pegRNA HEK4 (right). The majority of tested sites exhibited no significant editing, consistent with the high fidelity of prime editors. Only the site with the strongest Tracking-seq2 signal (V1&V2-01) contained substantial off-target editing. *P*-values were calculated by one-sided binomial tests and the exact value for each site is listed in Supplementary Table 3. **h**, Tracking-seq2 signals at representative off-target sites for PE3 + pegRNA HEK4. PE3 + pegRNA VEGFA2 serves as a non-targeting control. Distinct signals were observed at these off-target sites lacking detectable mutations, suggesting that DNA nicking events occurred at these loci but are efficiently repaired in an error-free manner.

To validate the additional off-target sites detected by Tracking-seq2 for CBE, ABE, and PE in HEK293T cells, we performed amplicon sequencing at the top five novel sites under each condition (CBE-VEGFA2, CBE-HEK4, ABE-VEGFA2, ABE-HEK4, PE3-VEGFA2, and PE3-HEK4). For CBE and ABE, all tested sites exhibited C to T or A to G editing, if there were C or A within the editing window **(Fig. R7c-f)**. For 4 sites of ABE lacking an A in the editing window, low-frequency indels were detected in 3 of them **(Fig. R7d)**. For PE3, we evaluated all types of substitutions and indels for off-target editing, but the majority exhibited no significant editing **(Fig. R7g)**. Given that only three novel off-target sites were identified for PE3-HEK4, we additionally validated four sites jointly detected by both Tracking-seq V1 and V2. The results revealed that only the site with the strongest Tracking-seq2 signal (V1&V2-01) contained substantial off-target editing. Considering the presence of distinct Tracking-seq2 signals at mutation-negative sites **(Fig. R7h)**, we hypothesize that while DNA nicking events do occur at these loci, they are efficiently repaired in an error-free manner. Our findings are consistent with prior reports[22,31] demonstrating that PE exhibited high fidelity with much lower off-target effects.

Reference:

22. Anzalone, A. V. *et al.* Search-and-replace genome editing without double-strand breaks or donor DNA. *Nature* **576**, 149-157 (2019). https://doi.org:10.1038/s41586-019-1711-4

31. Gao, R. Z. *et al.* Genomic and Transcriptomic Analyses of Prime Editing Guide RNA-Independent Off-Target Effects by Prime Editors. *Crispr Journal* **5**, 276-293 (2022). https://doi.org:10.1089/crispr.2021.0080

It remains unclear to readers that users of Tracking-seq2 must perform two separate rounds of experiments—one using exonuclease and NHEJ inhibition to identify candidate off-targets, and another round without these modulators to confirm the true sites through targeted deep sequencing. The authors should discuss whether this workflow provides a practical advantage over Tracking-seq1.

Reply:

Thank you for highlighting this critical aspect of the Tracking-seq2 workflow and prompting us to clarify its practical implications.

Indeed, Tracking-seq2 utilizes a two-step workflow: an initial "detection phase" using exonuclease and NHEJ inhibition to maximize sensitivity, followed by a "validation" phase using targeted deep sequencing under standard physiological conditions (without modulators) to confirm true off-target effects. We added a clearer workflow as Fig. 1a in the revised manuscript.

This two-step workflow is also required by many other off-target detection methods, such as GUIDE-seq, GUIDE-seq-2 and DISCOVER-seq⁺. To ensure this workflow is entirely clear, we have explicitly illustrated the two-step process in the manuscript and, as suggested, have added a discussion in the Discussion section detailing its trade-offs and its advantages over single-step methods like Tracking-seq1.

[Figure Redacted]

Fig. R8 (Corresponding to Fig. 1a in the revised manuscript). Schematic illustration of the Tracking-seq2 workflow for Cas9 and Cas12a off-target detection. Exogenous T5 exonuclease treatment and NHEJ inhibition (using KU-60648) synergistically enhance the detection of Cas9 and Cas12a off-target sites by increasing the accumulation of RPA-bound ssDNA intermediates. The Tracking-seq2 workflow consists of two distinct phases: (1) Detection Phase—cells treated with T5 exonuclease and NHEJ inhibitor are used for the Tracking-seq2 assay to enrich ssDNA intermediates and maximize detection sensitivity; (2) Validation Phase—cells edited without T5 exonuclease and NHEJ inhibitor are used for targeted deep sequencing to measure actual off-target efficiency under physiological conditions.

Moreover, similar validation using the "track score mock" approach was not performed for base editors and prime editors and should be included.

Reply:

Thank you for this suggestion. We have now included the validation for BEs and PE in the revised manuscript, as described in the response above.

2. The manuscript refers to Tracking-seq2 as "unprecedentedly sensitive," "the most sensitive to date," and yet benchmarking is restricted to in silico tools and cell-free assays such as CIRCLE-seq, using only VEGFA2 and HEK4 guides. No direct comparisons with established in-cell methods (e.g., GUIDE-seq) or clinically relevant guides are presented. These claims should be either moderated or supported by appropriate experiments.

Reply:

Thank you for pointing this out. We agree that the initial claims were overstated given the limited benchmarking data provided. We have removed the phrases "unprecedentedly sensitive" and "the most sensitive to date", and the manuscript now objectively describes the sensitivity improvements based on the comparative data.

Furthermore, we have added direct benchmarking comparisons with the state-of-the-art in-cell method GUIDE-seq-2 and another computational tool COSMID. These experiments were conducted in both HEK293T cells and primary human T cells.

In HEK293T cells, Tracking-seq2 demonstrated superior sensitivity. Specifically, for both HEK4 and VEGFA2 targets, Tracking-seq2 identified a substantial number of additional off-target sites while capturing the vast majority of sites detected by GUIDE-seq-2 **(Fig. R9a)**. We conducted targeted amplicon sequencing for novel off-target sites that were not included in our hybrid capture data (i.e. unique novel sites from GUIDE-seq-2 or COSMID) and analyzed the distribution of Δindel rates (indel rates of Cas9-edited samples minus indel rates of WT samples) among different subsets of sites **(Fig. R9b)**. This result confirmed that Tracking-seq2 detected a lot of bona fide off-target sites missed by GUIDE-seq-2 or COSMID, while these two methods also provided several true off-target sites missed by Tracking-seq2.

In primary T cells, three methods shared common off-target sites and also included unique sites **(Fig. R9c).** To validate these unique sites, we performed amplicon sequencing on the top-10 unique sites specific to each method. The results showed that Tracking-seq2-only sites had higher Δindel rates than the others, while the other two methods also identified several genuine editing sites **(Fig. R9d)**.

These new data provide solid evidence that Tracking-seq2 identified additional off-target sites missed by the other methods, demonstrating sensitivity comparable to, or exceeding that of current methods.

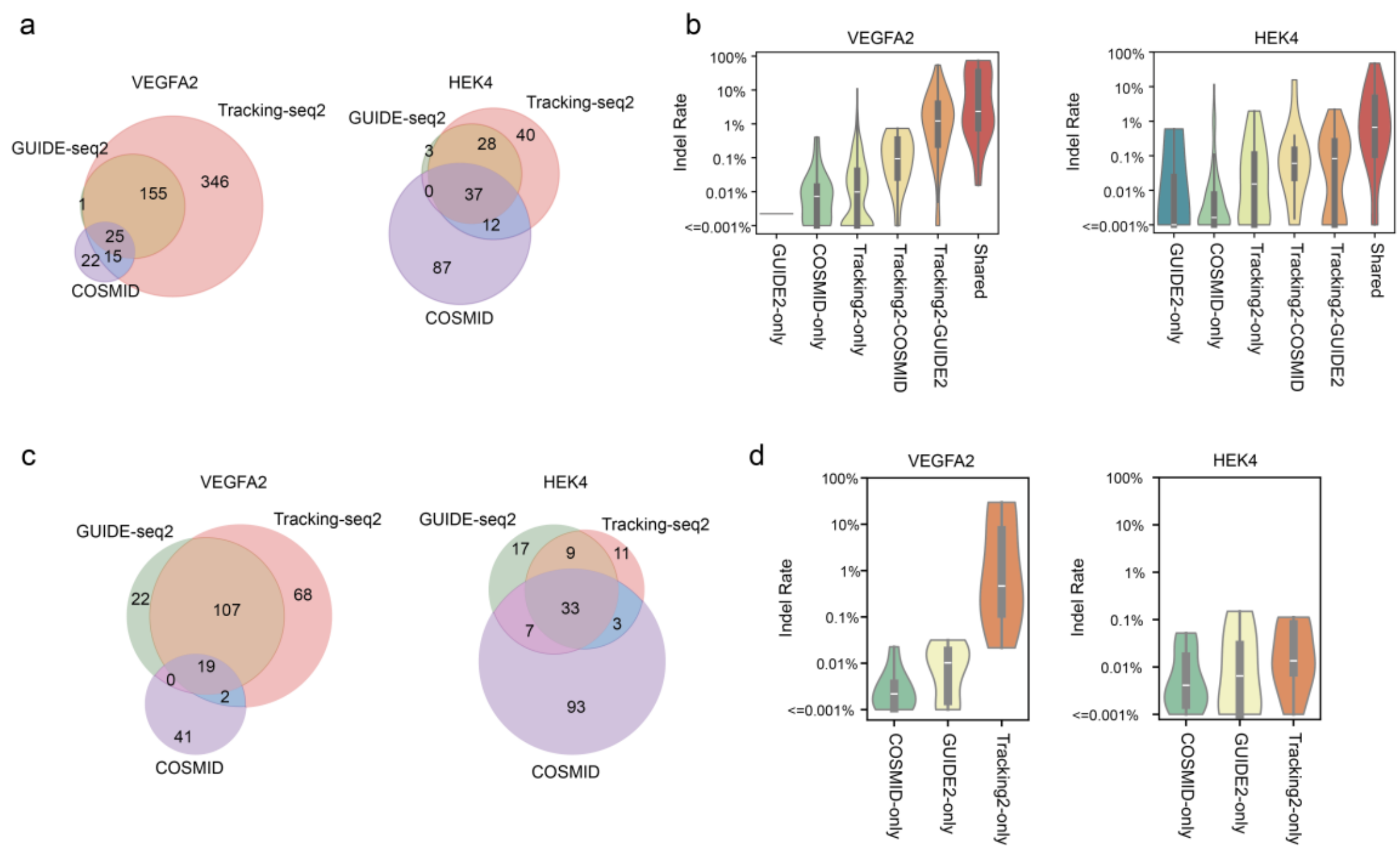

Fig. R9 (Corresponding to Fig. 2c-d and Fig. 4e-f in the revised manuscript). a, Venn diagrams comparing off-target sites detected (or predicted) by Tracking-seq2, GUIDE-seq-2, and COSMID for Cas9 + sgRNA VEGFA2 or Cas9 + sgRNA HEK4. **b**, Box plot showing the distribution of Δindel rates measured by hybrid capture targeted sequencing at off-target sites belonging to different sets for Cas9 + VEGFA2 and Cas9 + HEK4. GUIDE-seq-2 and COSMID missed a considerable number of off-target sites detected by Tracking-seq2. **c**, Venn diagrams comparing off-target sites detected (or predicted) by Tracking-seq2, GUIDE-seq-2, and COSMID for Cas9 + VEGFA2 or Cas9 + HEK4 in human T cells. **d**, Box plot showing the distribution of Δindel rates measured by targeted

amplicon sequencing at method-specific off-target sites (top10 per method) for Cas9 + VEGFA2 or Cas9 + HEK4 in human T cells. Off-target sites exclusively identified by Tracking-seq2 exhibited the highest indel frequencies compared to those uniquely detected by GUIDE-seq-2 or COSMID.

3. The timing of sample collection (24 h and 48 h post-plasmid transfection) could influence outcomes. Since plasmid-driven Cas expression can persist beyond 48 h, additional off-target events might occur at later time points once intended target sites are modified. This possibility should be discussed.

Reply:

Thank you for this insightful comment regarding the kinetics of off-target accumulation. We agree that plasmid-driven Cas9 expression can persist beyond 48 hours, potentially leading to off-target events at later time points that might be missed by early sampling.

Our chosen timepoints were based on our previous Tracking-seq1 study, where we performed a time-course experiment at 12, 24, 48, 72, and 96 hours post-transfection. By measuring the intensity of the captured RPA-bound single-stranded DNA (ssDNA) signals at each timepoint, we determined that the peak signal occurred at 24 h for BEs and 48 h for Cas9 and PEs **(Fig. R10)**. We therefore selected these specific peak timepoints to maximize the discovery sensitivity of Tracking-seq2. To ensure we captured late-occurring edits in the validation phase, we extended the culture time to 96 h post-transfection for targeted amplicon sequencing and hybrid capture sequencing.

Furthermore, during our direct benchmarking against other methods, we noticed that Tracking-seq2 missed some validated low-frequency off-target sites. We hypothesize that these events were missed because the editing likely occurred after our sample collection window. We have expanded the Discussion section to illustrate this limitation.

[Figure Redacted]

Fig. R10 (from our previous study (Zhu, M. *et al.*)). The temporal dynamics of Tracking-seq signals. Timepoints represent the intervals after the transfection of HEK293T cells. 'Background' refers to the mean score of all negative candidate off-target sites

Reference:

Zhu, M. *et al.* Tracking-seq reveals the heterogeneity of off-target effects in CRISPR-Cas9-mediated genome editing. *Nature Biotechnology* **43**, 799–810. (2024). https://doi.org:10.1038/s41587-024-02307-y

4. The validation is based solely on indel frequency as a marker of genuine off-target activity. However, CRISPR off-target events can also result in chromosomal rearrangements, structural variations (e.g., translocations, inversions, copy number changes), or transcriptional alterations not captured by indel analysis. These unaccounted effects need to be acknowledged as limitations.

Reply:

Thank you for this insightful comment. We agree that a comprehensive safety assessment extends beyond indel frequency to include structural variations (SVs) such as large deletions, translocations, and copy number changes.

Tracking-seq2 is designed to map CRISPR-induced DNA cleavage loci genome-wide, which represent the initiating events that could potentially lead to such SVs. However, Tracking-seq2 does not provide the direct information of SVs and our current validation method (targeted amplicon sequencing and hybrid capture sequencing) is limited to detecting small indels and cannot capture SVs. We have revised the Discussion section to explicitly acknowledge this limitation.

5. The authors state that Tracking-seq2 supports patient-specific safety testing using as few as 10,000–50,000 primary cells, while the Methods section specifies approximately 500,000 cells per experiment. This inconsistency requires clarification.

Reply:

We apologize for the ambiguity regarding cell input. We have clarified that "10,000–50,000 cells" represents the minimal viable input, while "~500,000 cells" is the standard condition for optimal sensitivity.

In the revised manuscript, we added experiments to compare the results from inputs of 10,000, 50,000, 100,000, and 500,000 cells in HEK293T and primary T cells. Compared to the 500,000-cell baseline, Tracking-seq2 with 50,000 or 100,000 cells resulted in only a slight reduction in detected off-target sites for both cell types **(Fig. R11)**. However, with an input of 10,000 cells, weak off-target signals became indistinguishable from background noise. Therefore, we recommend ≥50,000 cells for primary cells to balance sensitivity with sample availability. We have updated the Results sections in the revised manuscript to explicitly state these results.

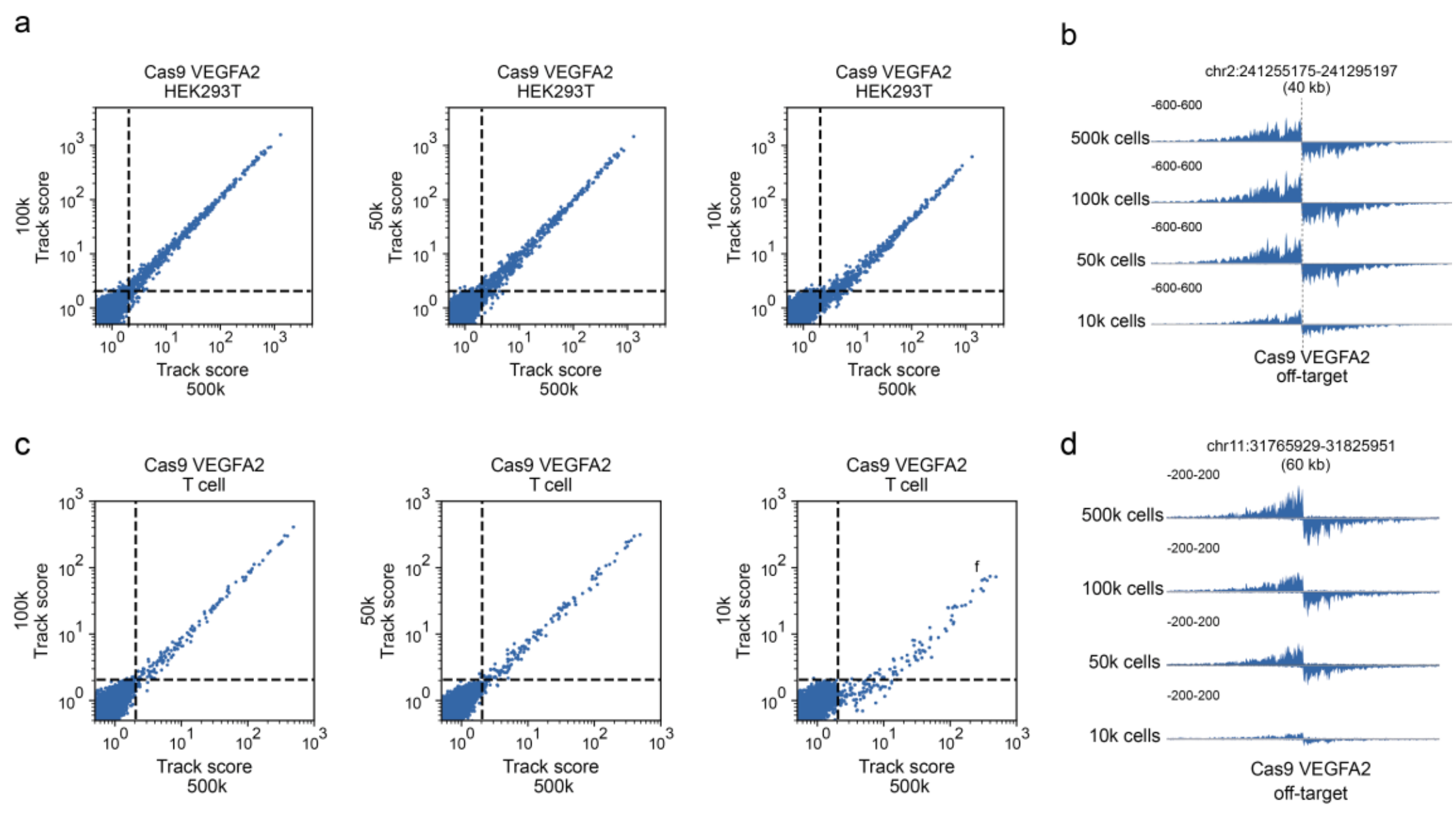

Fig. R11 (Corresponding to Extended Data Fig. 9 and Fig. 4g-h in the revised manuscript). a, Correlation between Track scores in experiments with different HEK293T cells (10,000, 50,000, 100,000, or 500,000 cells) edited by Cas9 + sgRNA VEGFA2. Each dot represents a candidate off-target site. Dashed lines represent the thresholds of the Track score > 2 for positive sites. Sensitivity with 100,000 or 50,000 cells was similar to that with 500,000 cells, while assays using 10,000 cells

missed off-target sites with low signals. **b**, Tracking-seq signals at a representative off-target site using different numbers of HEK293T cells. **c,** Correlation between Track scores in experiments with varying human T cell inputs (10,000, 50,000, 100,000, and 500,000 cells). Each dot represents a candidate off-target site. Sensitivity with 100,000 or 50,000 cells was only slightly lower than that with 500,000 cells, while assays using 10,000 cells missed off-target sites with low signals. **d**, Tracking-seq2 signals at a representative off-target site using different numbers of human T cells.

6. The computational pipeline is insufficiently described. Critical aspects such as "track score" calculation, peak calling strategy, background correction, replicate treatment, multimapping read management, and thresholding criteria should be explicitly detailed to ensure reproducibility.

Reply:

Thank you for this important comment. We added a brief description of computational analysis in the Introduction section: "The Offtracker software calculates a normalized "Track score" for each candidate off-target site, which represents the intensity of Tracking-seq signal, and the empirical threshold for a positive site is Track score > 2."

The analyses of Tracking-seq/Tracking-seq2 data were performed with Offtracker, a tailored end-to-end software without peak calling. To ensure the pipeline is fully transparent and reproducible, we added an "Analysis of Tracking-seq and Tracking-seq2 data" section in the Methods section as follows:

"The sequencing data of Tracking-seq/Tracking-seq2 were analyzed using Offtracker with default parameters. The software and a detailed pipeline are available at GitHub (https://github.com/Lan-lab/offtracker). Offtracker is an end-to end software tailored for Tracking-seq/Tracking-seq2 data. In brief, fastq files were mapped to hg38 reference genome with chromap[34] in a strand-specific manner with "-l 3000 --low-mem --BED --remove-pcr-duplicates --trim-adapters --min-read-length 10 --allocate-multi-mappings" parameters. All candidate off-target sites of a given sgRNA were enumerated by blastn-short[35] with word size = 4. Offtracker calculated Track scores for each candidate site according to the normalized read counts mapped to the forward strand and the reverse strand around the site. Offtracker deducted normalized signals in the control group from those in the experimental group to distinguish signals caused by the specific genome editor + gRNA from any source of background noise. The Track score is a normalized score reflecting signal enrichment and strand specificity for each site with an empirical threshold > 2 for calling positive off-target sites. More detailed, step-by-step codes and instruction can be found in our Nature Protocols[36] and our GitHub page for the latest update. For replicates in the same condition, the mean Track scores were calculated for scatter plots and subsequent analyses."

Reference:

34 Zhang, H. *et al.* Fast alignment and preprocessing of chromatin profiles with Chromap. *Nat Commun* **12**, 6566 (2021). https://doi.org:10.1038/s41467-021-26865-w

35 Sayers, E. W. *et al.* Database resources of the national center for biotechnology information. *Nucleic Acids Res* **50**, D20-d26 (2022). https://doi.org:10.1093/nar/gkab1112

36 Xu, R. *et al.* Tracking-seq: a universal off-target detection approach for CRISPR-Cas genome editing. *Nat Protoc* Advance online publication. (2026). https://doi.org:10.1038/s41596-025-01331-9

7. The manuscript reports detection of robust Cas9 signals at sites with up to seven mismatches, suggesting extreme mismatch tolerance. This observation requires additional validation or discussion to clarify whether such sites truly reflect Cas9-mediated cleavage or arise from increased ssDNA generation under T5/NHEJ inhibition conditions.

Reply:

Thank you for the insightful comments regarding the credibility of off-target sites with high mismatches. We found 3 sites with 7 mismatches that were only detected by Tracking-seq2 in HEK293T cells, and there were relatively more sites with 6 mismatches, some of which were also detected by other methods.

To further validate such sites, we performed amplicon sequencing on the 3 off-target sites with 7 mismatches, as well as 7 sites with 6 mismatches. Most of them were verified as true off-target events **(Fig. R12)**. All these amplicon sequencing experiments were performed with cells under physiological conditions (without T5/NHEJ inhibition) 120 hours post-transfection. These results have been added to the relevant sections of the revised manuscript.

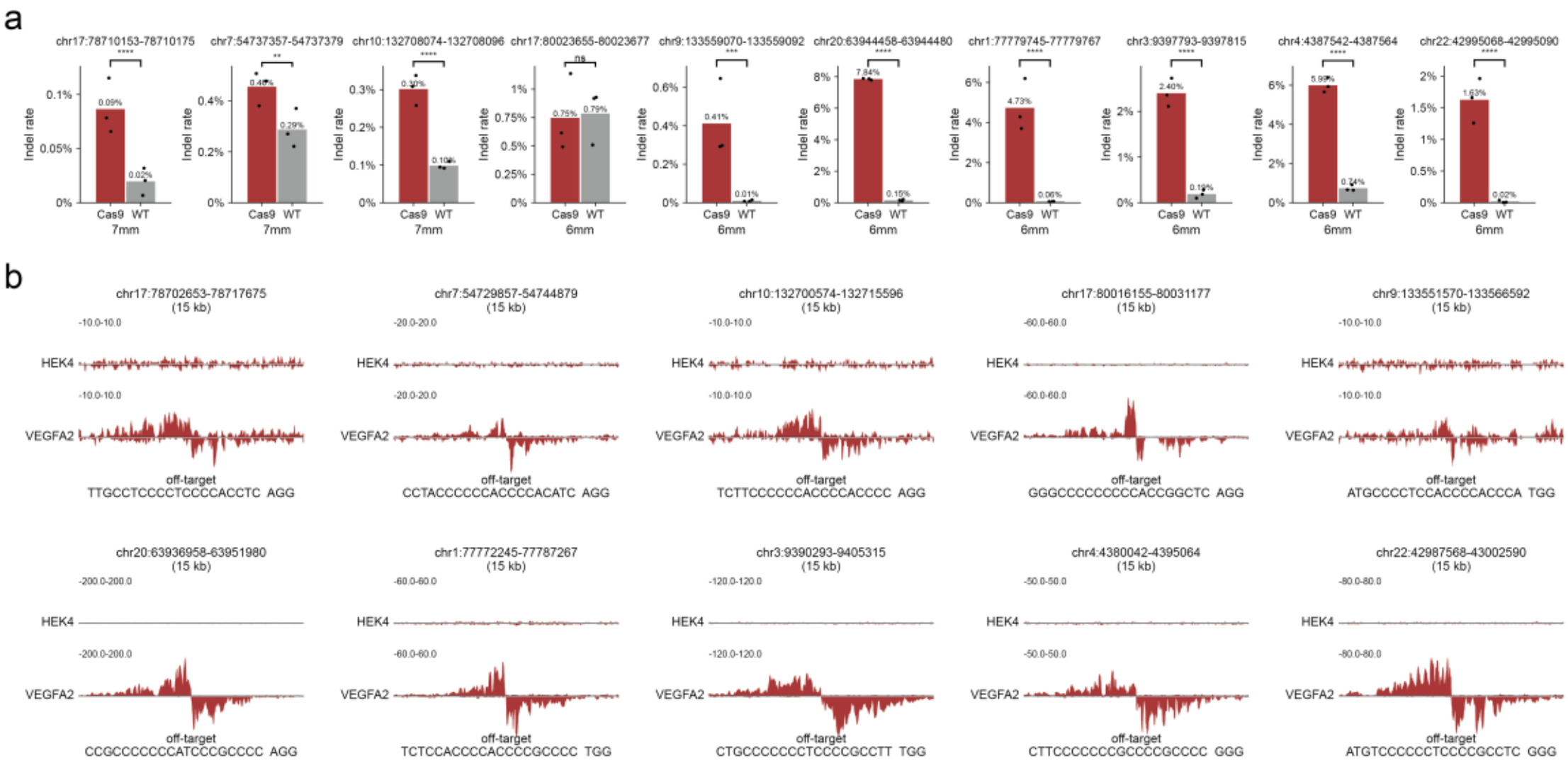

Fig. R12 (Corresponding to Extended Data Fig. 5c-d in the revised manuscript). a, Targeted amplicon sequencing validation of off-target sites with 6 or 7 mismatches detected by Tracking-seq2 for Cas9 + sgRNA VEGFA2 in HEK293T cells. 9 out of 10 were verified as true off-target events. *P*-values were calculated by one-sided binomial tests and the exact value for each site is listed in Supplementary Table 2. **b**, Tracking-seq2 signals at representative off-target sites with 6 or 7 mismatches for Cas9 + sgRNA VEGFA2 in HEK293T cells. Cas9 + sgRNA HEK4 served as a non-targeting control.

8. Does the HSPCs and the T-cell comes from same donor? It may be important to show that HSPCs or T cells of two-three donors to support the claim the donor genotype altering the off-targets.

Reply:

Thank you for this insightful comment. We clarify that the HSPCs and T-cells used in the original figures were derived from different donors.

In the revised manuscript, we performed additional experiments of primary T-cells, bringing the total to four distinct donors in total. Our results revealed evident inter-individual genetic diversity that is likely to derive differential off-target activity in particular donor’s cells **(Fig. R13)**.

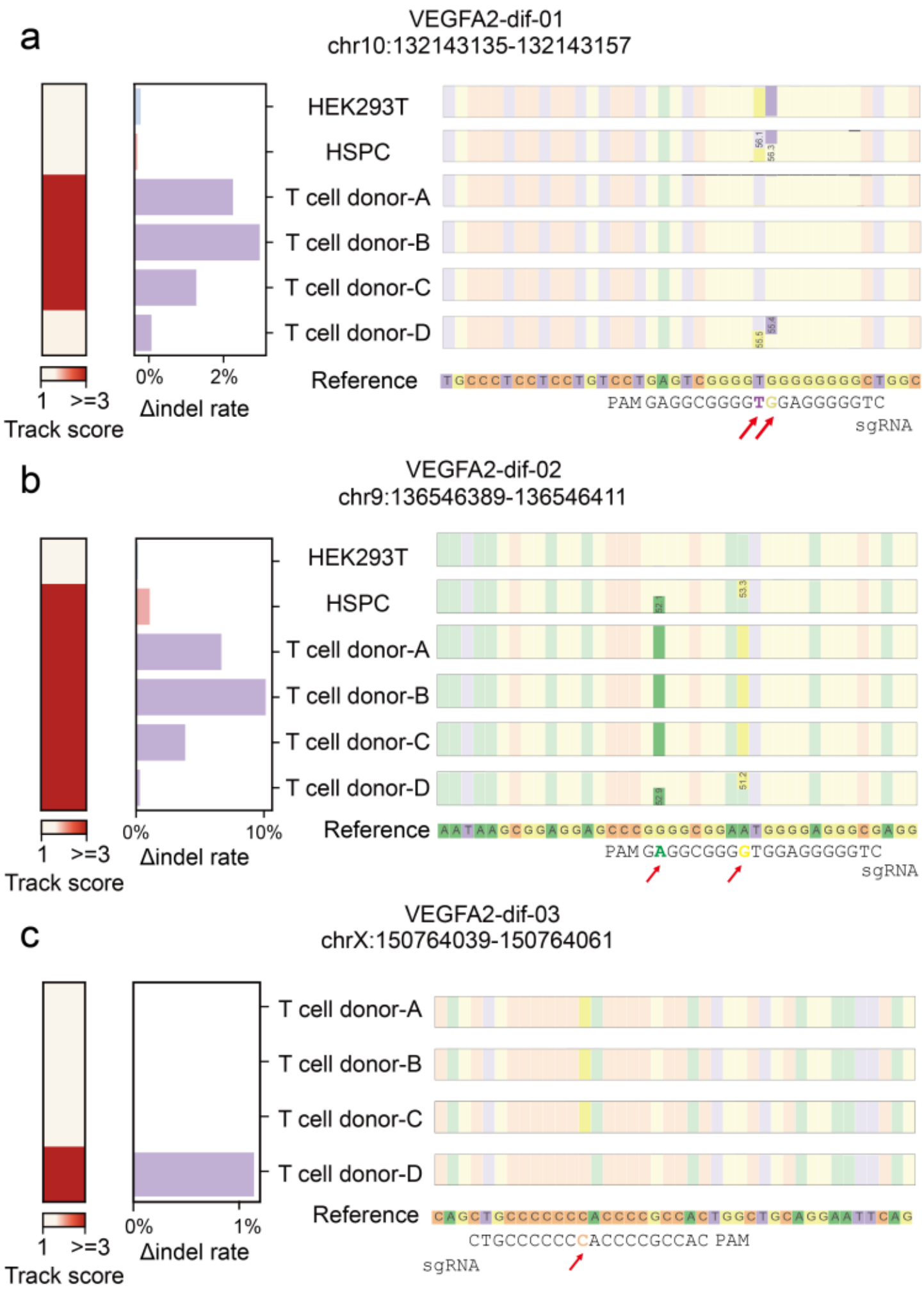

Fig. R13 (Corresponding to Fig. 5e-g in the revised manuscript). a-c, Genomic sequences of unedited HEK293T cells, HSPCs, and T cells from four different donors at the identified differential off-target sites for the sgRNA VEGFA2, as determined by targeted amplicon sequencing. For the VEGFA2-dif-01 locus (**a**), T cells from donors A, B, and C harbored a genomic sequence identical to the hg38 reference, which matched the sgRNA at the marked two nucleotides. In contrast, T cells from donor D and the HSPCs were heterozygous at these two nucleotides, while HEK293T cells were homozygous for the variant, both resulting in mismatches with the sgRNA sequence. For VEGFA2-dif-02 (**b**), T cells from donors A, B, and C contained two homozygous nucleotide polymorphisms that matched the sgRNA sequence, whereas HEK293T cells were homozygous at these two nucleotides that mismatched the sgRNA sequence. HSPCs and T cells from donor D were heterozygous at these positions, resulting in weak off-target editing. For VEGFA2-dif-03 (**c**), T cells from donors A, B, and C contained a homozygous nucleotide polymorphism that mismatched the sgRNA sequence, while T cells from donor D matched the nucleotide.

MINOR

Line 87 – Typo PARP1

Reply:

Thank you for their careful reading. We have corrected the typo regarding "PARP1" in the revised manuscript.

Reviewer #3

(Remarks to the Author):

I recommend this manuscript for publishing pending several minor formatting changes. The researchers have substantially expanded upon the capabilities of TraskingSeq by implementing several known methods of increasing homologous recombination. Furthermore, Cong, Xu, Chen, Yuan, et al show the increased specificity of TrackingSeq2 when compared to in vitro off-target gene editing analysis software, and exhibit the need for such a tool in the rapidly-developing world of gene editing therapies.

Reply:

Thank you very much for the positive evaluation of our study and the recommendation for publication. We have carefully revised the manuscript, expanded the Introduction and Discussion sections, and corrected the formatting issues to ensure compliance with the journal's standards.

Section-wise recommendations for changes are as follows:

Introduction:

The Introduction should suitably describe each of the cellular mechanisms that are relevant throughout the manuscript. There are several additions that, while not necessary, would improve communicability of the paper: these include brief descriptions of the proteins of interest in NHEJ and HR pathways (i.e. the role of RPA and Rad51 in ssDNA stability, the stabilizing effects of ssDNA donor templates, why 5′-3′ exonuclease treatment would affect HR, etc.), as well as including a brief description of the mechanism and protocol for the original TrackingSeq detection technology. One of the main goals of this manuscript is to outline how TrackingSeq2 is a significant advancement; adding a section to the introduction outlining how the current TrackingSeq assay is performed would be useful for this. Currently, TrackingSeq is described in the Introduction mainly to highlight its strengths compared to other analysis methods, which while accurate is better suited for the Discussion section.

Reply:

Thank you for these constructive suggestions to improve the clarity and structure of the Introduction. We have added a description of the original Tracking-seq workflow, as well as relevant cellular mechanisms detailing the HR and NHEJ pathways to introduce the rationale behind using exonuclease and NHEJ inhibitors in Tracking-seq2, as follows (Line #53-91):

"Replication protein A (RPA) is a single-stranded DNA (ssDNA) binding protein that is involved in both double-strand breaks (DSBs) and single-strand breaks (SSBs) repair pathways17,18. The original Tracking-seq technology captures editing-induced DNA lesions by tracking the genome-wide binding patterns of RPA, enabling the direct detection of off-target sites for diverse genome-editing tools. The workflow comprises four major steps: (1) capturing RPA-bound ssDNA using CUT&RUN; (2) removing double-stranded DNA (dsDNA) using HL-dsDNase; (3) performing strand-specific ssDNA library construction; and (4) next-generation sequencing (NGS) coupled with Offtracker bioinformatics analysis. The Offtracker software calculates a normalized "Track score" for each candidate off-target site, which represents the intensity of Tracking-seq signal, and the empirical threshold for a positive site is Track score > 2.

Cells primarily repair DSBs through two competing pathways: homologous recombination (HR) and non-homologous end joining (NHEJ). NHEJ directly ligates broken ends without extensive

DNA processing, whereas HR requires extensive end resection driven by endogenous exonucleases19, generating long ssDNA intermediates at the DSB ends. These intermediates are rapidly coated by RPA to stabilize the structures and are finally displaced by RAD5120. Therefore, the extensive RPA-ssDNA complexes make HR the main source of the signal detected by Tracking-seq for DSB-inducing editors (such as Cas9 and Cas12a). Based on this mechanism, we hypothesized that because HR and NHEJ are mutually competitive, inhibiting NHEJ would force a greater proportion of DSBs into the HR pathway, thereby increasing endogenous ssDNA generation. Additionally, the exogenous addition of a 5′→3′ exonuclease could extend the length of end resection. Consequently, combining NHEJ inhibition with exonuclease treatment should amplify the RPA-ssDNA signal and enhance the sensitivity of Tracking-seq for DSB-based editors.

In contrast, BEs and PEs function primarily through SSB repair mechanisms21,22. During SSB repair, ssDNA intermediates are formed and coated by RPA to stabilize the local structure and coordinate the recruitment of downstream repair factors18. Since NHEJ predominantly processes DSBs, inhibiting the NHEJ pathway is not applicable for SSB-generating editors such as BEs or PEs. Nevertheless, exogenous 5′→3′ exonuclease treatment can extend ssDNA regions at nicked DNA sites, thereby amplifying the detection signal of Tracking-seq.

Guided by this mechanistic understanding of distinct DNA repair pathways, we developed Tracking-seq2 by implementing editor-specific optimizations. For DSB-dependent editors such as Cas9 and Cas12a, we integrated 5′→3′ exonuclease treatment with inhibition of the NHEJ pathway. For SSB-dependent BEs and PEs, only 5′→3′ exonuclease treatment was applied to amplify off-target signals. These refinements enable Tracking-seq2 to achieve substantially improved sensitivity in genome-wide off-target profiling, while functioning effectively within patient-derived primary cells.”

In addition, as suggested, we have moved the detailed comparison between Tracking-seq2 and other methods to the Discussion section. The Introduction now focuses on the biological background and the rationale of Tracking-seq2.

Discussion:

Within the results, variability in the effectiveness of the assay changes is noted (i.e. Lines 145-147 denote how different target sites showed different changes in off-target detection with the addition of T5 exonuclease treatment. This points to a potentially major concern; if different sites are affected differently by the changes to the TrackingSeq2 method, then the changes introduced, while increasing sensitivity by amplifying rarer events to the point of detectability, are still insufficient to suitably replicate the action of a “real-time” CRISPR/Cas construct. This gap between the experimental conditions of the TrackingSeq2 gene editing reactions and other more complex and stochastic mechanisms at play in vivo should still be made clear.

Reply:

Thank you for raising this critical point. We agree that the site-specific variability in response to T5 exonuclease treatment points to an inherent gap between the Tracking-seq2 condition and the practical editing condition without T5 or drug treatment. Consequently, the amplified rare off-target events under the Tracking-seq2 condition may not always result in true off-target editing under an unperturbed condition. In fact, our downstream validation via targeted amplicon and hybrid capture sequencing confirmed that several newly detected off-target sites lacked detectable editing in unperturbed cells. This indicates that while Tracking-seq2 has enhanced sensitivity, it can

overestimate certain rare off-target events and raise false positives, underscoring the necessity of downstream validation (e.g., amplicon sequencing) in an unperturbed cellular environment to quantify authentic off-target editing frequencies. In response to your comment, we have expanded the Discussion to clearly articulate this limitation and the rationale behind our two-step validation strategy as follows (Line #299-313):

"Tracking-seq2 employs a two-step workflow to balance detection sensitivity with physiological relevance. The first "detection phase" utilizes T5 exonuclease and NHEJ inhibitors to perturb the repair landscape and amplify RPA-ssDNA signals, thereby maximizing the discovery of low-frequency off-target sites. As this treatment amplifies RPA-ssDNA intermediates and may overestimate rare off-target events, the second "validation phase" leverages targeted amplicon or hybrid capture sequencing under standard physiological conditions (without modulators) to accurately measure true off-target editing efficiencies. While the signal amplification during the detection phase alters the native repair environment, our validation phase confirmed that a considerable amount of the newly identified candidates represented genuine off-target events under physiological conditions. Although this two-step paradigm increases experimental complexity, it is also required by other state-of-arts methods (e.g., GUIDE-seq/GUIDE-seq-2, DISCOVER-seq^{+}). More importantly, this trade-off yields markedly enhanced sensitivity over the original Tracking-seq, fully justifying the additional effort."

Line-wise recommendations for changes are as follows:

Line 47 (Introduction): Please add a specific source(s) to support the statement "[Cell-free methods] exhibit poor clinical translatability due to fundamental discrepancies such as altered Cas9 kinetics in vitro and the lack of epigenetic context, therefore often suffers from high false positive rates."

Reply:

Thank you for pointing this out. We have added appropriate citations to support the statement about cell-free methods and their limitation in clinical translatability (Cromer, M. K. *et al.*).

Reference:

Cromer, M. K. *et al.* Comparative analysis of CRISPR off-target discovery tools following ex vivo editing of CD34^{+} hematopoietic stem and progenitor cells. *Molecular Therapy* **31**, 1074-1087 (2023). https://doi.org:10.1016/j.ymthe.2023.02.011

Line 80-86 (Results): This section discussing basic DSB repair mechanisms should be moved or otherwise included in the Introduction. The sentence in Line 86, "To identify potential inhibitors…" is a suitable start to this section of the Results, the rationale as to why should be in the Introduction.

Reply:

Thank you for this valuable feedback. We agree that the Results section should focus on the specific experimental outcomes rather than general background knowledge. We have moved the text describing basic DSB repair mechanisms to the Introduction. We believe this change improves the overall readability of the manuscript.

Reviewer #4

(Remarks to the Author):
Dr. Cong and colleagues developed Tracking-seq2, an advanced technology that integrates 5′→3′ exonuclease treatment and non-homologous end joining (NHEJ) pathway inhibitors with the original Tracking-seq. Overall, the idea of the paper is interesting, but in its current form the work is not ready for publication in Nature Communications.
Reply:
Thank you very much for your constructive assessment of our manuscript. Your insightful feedback has been valuable in helping us refine our work and strengthen the overall quality of our study.

Below are some overarching points that I believe are important to address in order to bring the study in line with the journal's standards:

1. Increase the number of biological replicates in most experiments (at least n=5 for example in Fig. 1b, d, e, I, 3a, 4a, currently with n=1 in main figures).
Reply:
Thank you for emphasizing the importance of biological replicates. We completely agree that increasing the number of biological replicates is essential for demonstrating the reproducibility and robustness of our results.

To address this, we have substantially expanded our experimental dataset. While we deeply respect the reviewer's suggestion to increase the replicates to n = 5, performing this across all the experimental conditions would require approximately 300 independent Tracking-seqV1/V2 assays (including new conditions like Td-CBE and HiFi Cas9 editing in the revised manuscript). Given the substantial cost, labor and the revision time limit, we communicated this with the handling editor and we reached an agreement that n = 3 is accepted for this type of NGS assay, considering experiments with different sgRNAs can be regarded as a kind of replication to demonstrate the robustness of Tracking-seq2 enhancement.

In the revised manuscript, we have conducted 151 additional Tracking-seqV1/V2 experiments to make sure n = 3 in each condition, including almost all Tracking-seqV1/V2 panels in main figures and Extended Data Figures. Regarding the primary cell datasets, given the high cost of acquiring and culturing HSPCs, we directed our resources toward primary T cells, expanding this dataset to n = 5 (2 independent samples from donor-A and 3 samples from donor-B, donor-C, and donor-D, respectively).

We hope this substantially expanded dataset firmly establishes the reproducibility of our method and robustly supports our conclusions.

2. Include an appropriate time course analysis to dissect off target events generated by the different editing platforms, ideally over at least a 7 day experimental window (now just 24 or 48h post treatments in vitro).
Reply:
We appreciate the reviewer's suggestion regarding a time-course analysis. We have already conducted such experiments in our previous Tracking-seq1 study, where we performed a time-course experiment at 12, 24, 48, 72, and 96 hours post-transfection. The results showed that signals

(measured by Track score) were strongest at 48 h post transfection for Cas9 and PE, as well as 24 h post transfection for ABE and CBE (Fig). We therefore selected these specific peak timepoints to maximize the discovery sensitivity.

[Figure Redacted]

Fig. R14 (from our previous study (Zhu, M. *et al.*)). The temporal dynamics of Tracking-seq signals. Timepoints represent the intervals after the transfection of HEK293T cells. 'Background' refers to the mean score of all negative candidate off-target sites

Zhu, M. *et al.* Tracking-seq reveals the heterogeneity of off-target effects in CRISPR-Cas9-mediated genome editing. *Nature Biotechnology* **43**, 799–810. (2024). https://doi.org:10.1038/s41587-024-02307-y

3. For CBEs, ABEs and PEs, target additional loci and, where possible, compare older versus newer editor versions, as the study currently uses CBE4max, which is not among the latest CBE variants.

Reply:

Thank you for suggesting the inclusion of additional loci and updated editor versions to enhance the comprehensiveness of our study. We have expanded the experiments as follows:

We have added two additional target loci for Cas9 (IDS and VEGFA3), and one additional target locus for CBE (EMX1), ABE (VEGFA3), and PE3 (VEGFA3) for comparison between Tracking-seq2 and the original Tracking-seq. The results consistently demonstrate that Tracking-seq2 exhibits better sensitivity in detecting off-target events **(Fig. R15a-d)**.

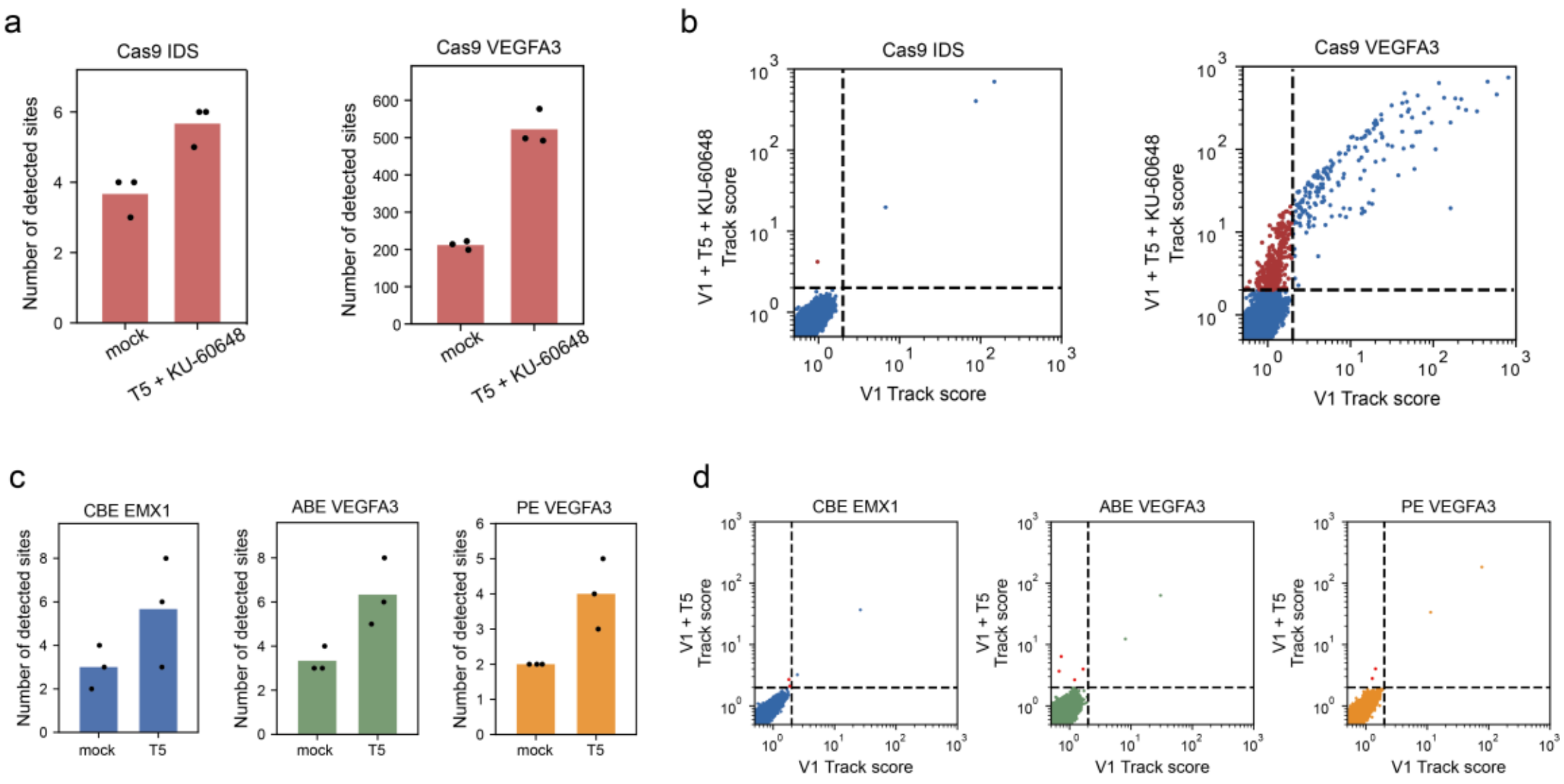

Fig. R15 (Corresponding to Extended Data Fig. 4e-h and Extended Data Fig. 6e-f in the revised manuscript). a, Numbers of off-target sites caused by Cas9 + sgRNA IDS or sgRNA VEGFA3 detected by Tracking-seq in mock group and combined treatment of T5 and KU-60648 group. Each dot represents an independent biological replicate (n=3). **b**, Scatter plot showing the

Track scores of Tracking-seq with combined T5 co-transfection and KU-60648 treatment versus those without treatment edited by Cas9 + sgRNA IDS or sgRNA VEGFA3. Each dot represents a candidate off-target site. Dashed lines represent the thresholds of the Track score > 2 for positive sites. **c**, Numbers of off-target sites caused by CBEmax + sgRNA EMX1, ABE + sgRNA VEGFA3, PE3 + pegRNA VEGFA3, and detected by Tracking-seq in mock group and combined treatment of T5 and KU-60648 group. Each dot represents an independent biological replicate (n = 3). **d**, Scatter plot showing the Track scores of Tracking-seq with T5 co-transfection versus those without treatment edited by CBEmax + sgRNA EMX1, ABE + sgRNA VEGFA3, PE3 + pegRNA VEGFA3, respectively. Each dot represents a candidate off-target site.

For the suggestion of latest CBE versions, we included a TadA-derived CBE variant, Td-CBEmax (Td-CBE) **(Fig. R16)**. We tested Td-CBE with sgRNAs VEGFA2 or HEK4, and then compared its off-target profile with that of CBE4max. Though Td-CBE generated fewer off-target sites than CBE4max, the two versions of CBE produced mutually exclusive off-target sites (Fig. **R16c, g)**, which were validated by amplicon sequencing **(Fig. R16d, h-j)**.

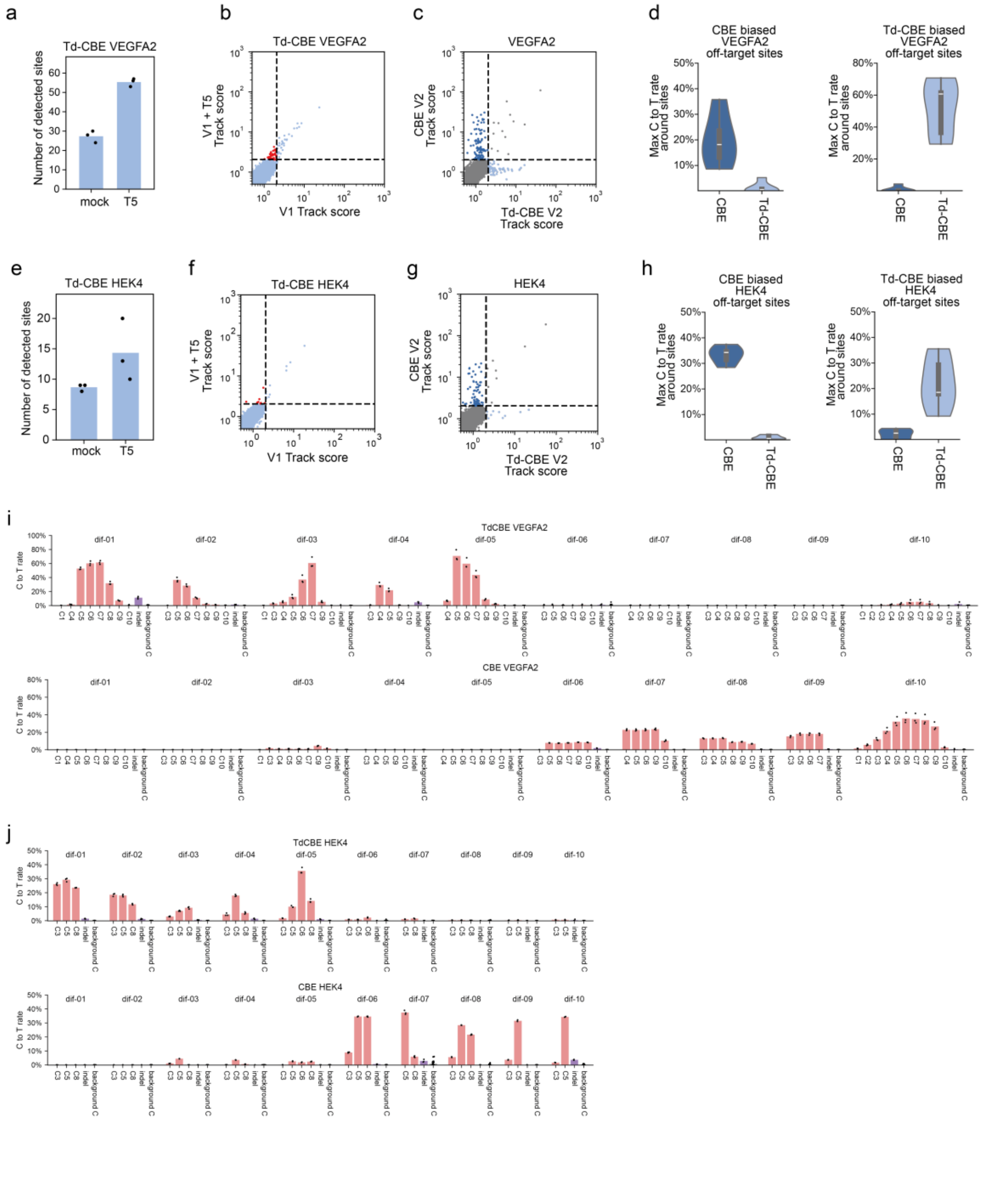

Fig. R16 (Corresponding to Fig. 3e-h and Extended Data Fig. 8 in the revised manuscript). a, Numbers of off-target sites caused by Td-CBEmax (a TadA-derived CBE variant) + sgRNA VEGFA2 detected by Tracking-seq in mock group and T5 group. Each dot represents a biological replicate (n = 3). **b**, Scatter plot showing the Track scores of Tracking-seq with T5 co-transfection treatment versus those without treatment edited by Td-CBEmax + sgRNA VEGFA2. Each dot represents a candidate off-target site. Dashed lines represent the thresholds of the Track score > 2 for positive sites. **c**, Scatter plots comparing the off-target sites detected for CBE4max and Td-CBEmax in HEK293T cells using sgRNA VEGFA2. **d**, Box plots showing the maximum C-to-T editing efficiency of 5 tested CBE4max-biased off-target sites (left) and 5 tested Td-CBEmax-biased off-target sites (right) in HEK293T cells (sgRNA VEGFA2). **e**, Numbers of off-target sites caused by Td-CBEmax + sgRNA HEK4 detected by Tracking-seq in mock group and T5 group. Each dot represents a biological replicate (n=3). **f**, Scatter plot showing the Track scores of Tracking-seq with T5 co-transfection treatment versus those without treatment edited by Td-CBEmax + sgRNA HEK4. Each dot represents a candidate off-target site. Dashed lines represent the thresholds of the Track score > 2 for positive sites. **g**, Scatter plots comparing the Track scores of off-target sites detected for CBE4max and Td-CBEmax in HEK293T cells using sgRNA HEK4. **h**, Box plots showing the maximum C-to-T editing efficiency of 5 tested CBE4max-biased off-target sites (left) and 5 tested Td-CBEmax-biased off-target sites (right) in HEK293T cells (sgRNA HEK4). **i-j**, Bar chart showing the C-to-T conversion rates at 5 Td-CBEmax-biased off-target sites (dif-01 to dif-05) and 5 CBE4max-biased off-target sites (dif-06 to dif-10) generated with sgRNA VEGFA2 (**i**) or sgRNA HEK4 (**j**) for the two versions of CBE. Background C: C outside the editing window.

Besides, we also added experiments with HiFi Cas9 in HEK293T cells in the revised manuscript. As expected, HiFi Cas9 showed substantially fewer detected off-target sites compared to WT Cas9 **(Fig. R17)**. These data confirm that Tracking-seq2 effectively captures off-target profiles for different editors, providing insights into their specificity.

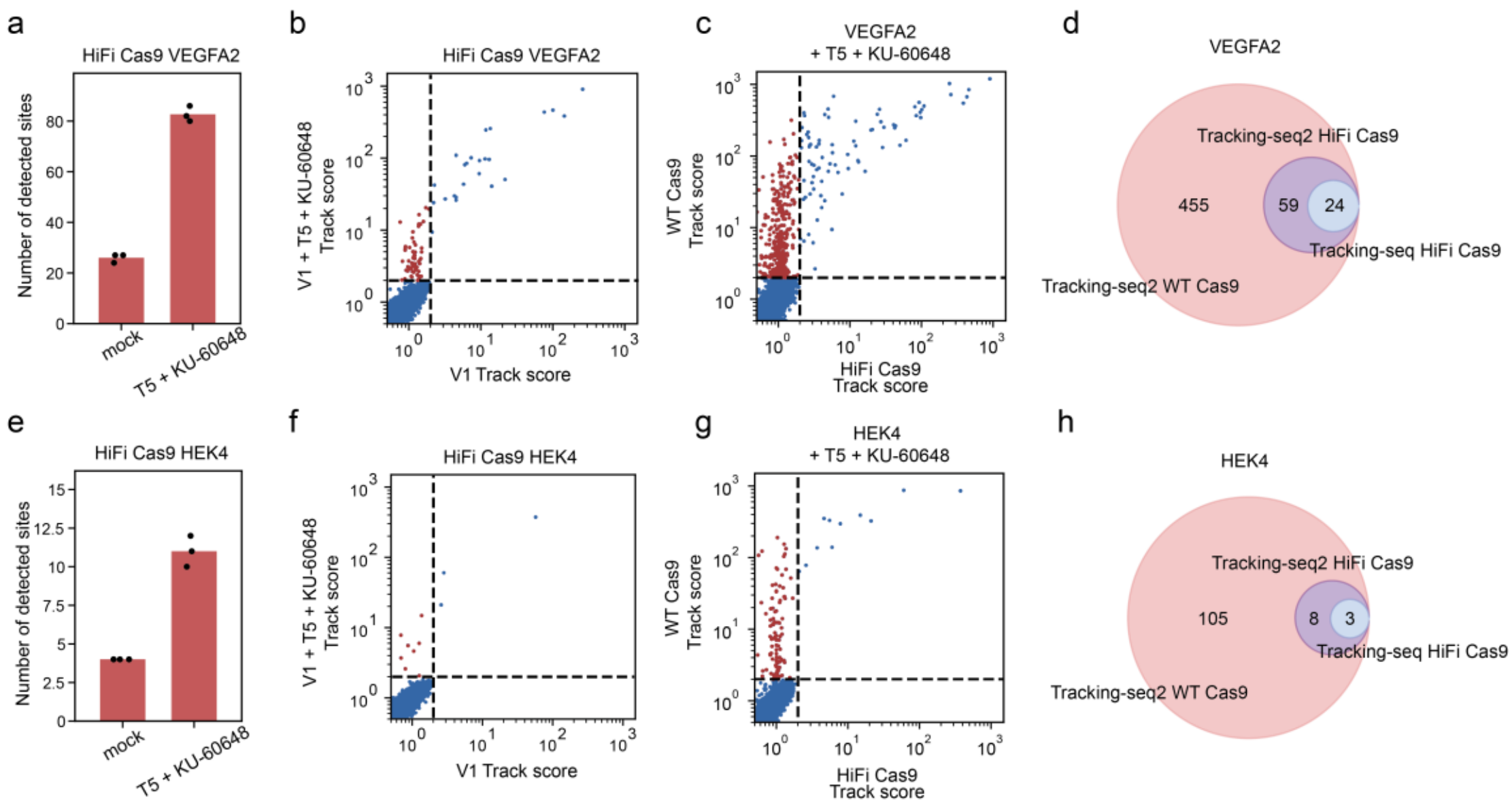

Fig. R17 (Corresponding to Extended Data Fig. 4i-p in the revised manuscript). a, Numbers of off-target sites caused by HiFi Cas9 + sgRNA VEGFA2 detected by Tracking-seq in mock group and combined treatment of T5 and KU-60648 group. Each dot represents an independent biological

replicate (n = 3). **b**, Scatter plot showing the Track scores of Tracking-seq with combined T5 co-transfection and KU-60648 treatment versus those without treatment edited by HiFi Cas9 + sgRNA VEGFA2. Each dot represents a candidate off-target site. Dashed lines represent the thresholds of the Track score > 2 for positive sites. **c**, Scatter plots comparing the Track score of off-target sites caused by Cas9 and those caused by HiFi Cas9, in HEK293T cells (VEGFA2). **d**, Venn diagram comparing off-target sites generated by Cas9 or HiFi Cas9 + VEGFA2, detected by Tracking-seq without treatment (mock) or by Tracking-seq2 (combined treatment of T5 and KU-60648). **e**, Numbers of off-target sites caused by HiFi Cas9 + sgRNA HEK4 detected by Tracking-seq in mock group and combined treatment of T5 and KU-60648 group. Each dot represents an independent biological replicate (n = 3). **f**, Scatter plot showing the Track scores of Tracking-seq with combined T5 co-transfection and KU-60648 treatment versus those without treatment edited by HiFi Cas9 + sgRNA HEK4. Each dot represents a candidate off-target site. Dashed lines represent the thresholds of the Track score > 2 for positive sites. **g**, Scatter plots comparing the Track scores of off-target sites caused by Cas9 and those caused by HiFi Cas9, in HEK293T cells (HEK4). **h**, Venn diagram comparing off-target sites generated by Cas9 or HiFi Cas9 + sgRNA HEK4, detected by Tracking-seq without treatment (mock) or by Tracking-seq2 (combined treatment of T5 and KU-60648).

4. Test Tracking seq2 in vivo after several months, for example by measuring off targets in T cells or mainly in HSPCs edited ex vivo and then transplanted into immunodeficient mice. This experiment is extremely relevant to assess potential selection and expansion of edited clones in repopulating assays that better approximate human settings.

Reply:

We sincerely thank the reviewer for this suggestion. We agree that investigating the long-term clonal dynamics of edited cells in a transplant setting is highly valuable for clinical translation. However, both Tracking-seq and Tracking-seq2 are based on capturing RPA-bound ssDNA intermediates during active DNA repair pathways, thus they cannot detect off-target sites after the editing event is finished after several months. Such long-term study can be performed with tag-based off-target detection methods, which insert specific DNA tags into the off-target sites permanently. We have discussed the restricted temporal detection window of Tracking-seq2 as a limitation in the revised Discussion section (Line #336-338) as follows:

"Second, because the assay captures transient RPA-ssDNA intermediates, it is inherently constrained to specific temporal windows of editing and may overlook late-occurring off-target events beyond early sampling timepoints."

5. Address off target detection in the context of homology-directed repair (HDR). It would be valuable to extend the method and discussion to HDR based therapeutic approaches. In particular, what is the impact of AAV6 mediated HDR on off target events, and how does this compare with the use of ssODN donors?

Reply:

We appreciate the reviewer's suggestion to address off-target detection in the context of HDR. We agree that understanding the impact of donor templates (such as AAV6 vs. ssODN) on off-target profiles is of great significance for therapeutic applications. However, given the limited time of revision, the main objective of the current study is to validate the enhanced technical sensitivity of Tracking-seq2 as a detection method. A systematic comparison of HDR-mediated editing involves

distinct biological variables that warrant a dedicated investigation, which warrants further investigation in the future.

6. Clarify whether HSPCs are derived from cord blood (CB) or mobilized peripheral blood (mPB), as editing efficiencies differ between these sources and may influence off target generation. I would suggest to compare off target profiles between CB and mPB derived HSPCs using Tracking seq28 at least in tow different genomic loci.

Reply:

Thank you for pointing this out. We have clarified in the revised Methods section that the HSPCs used in this study were derived from cord blood (CB).

Regarding the comparison between CB- and mPB-derived HSPCs, we respectfully note that obtaining mPB-derived HSPCs is challenging for us. More importantly, the primary focus of the current study is to establish Tracking-seq2 and validate its technical sensitivity. We agree that your excellent suggestion of comparing off-target profiles in different HSPC sources is a valuable direction for dedicated biological studies.

7. Because each locus has a distinct chromatin environment that affects editing efficiency and off target formation, consider testing Tracking seq2 at additional loci edited with different platforms (e.g. CBE, ABE, PE, Cas9) to assess locus dependent performance.

Reply:

Thank you for this insightful comment. We have expanded our study to include additional sgRNAs (two for Cas9, and one each for CBE, ABE, and PE3), as described above. These additional data confirm that Tracking-seq2 maintains better sensitivity across different loci.

Furthermore, as the off-target sites for a given sgRNA are widely distributed across the genome, we analyzed the chromatin environments surrounding all these sites. For the three highly active sgRNAs (HEK4, VEGFA2, and VEGFA3, each yielding >100 off-target sites), we profiled DNase-seq, H3K4me3, and H3K27ac signals within a 1kb region around each off-target site **(Fig. R18)**. Most detected off-target sites were located in regions with low chromatin accessibility, they spanned a wide variety of epigenetic contexts with similar distribution patterns across different sgRNAs. Collectively, these findings indicate that Tracking-seq2 effectively captures off-target events across a broad spectrum of epigenetic landscapes.

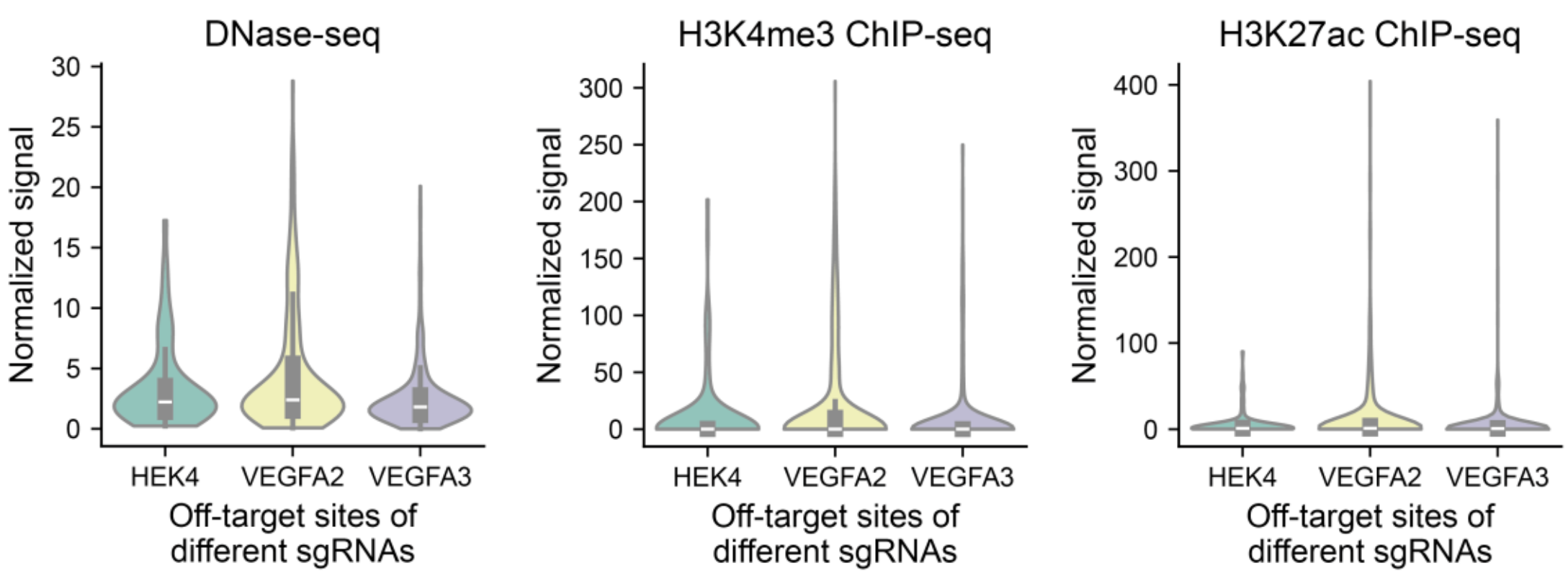

Fig. R18. The distribution of signal intensity of DNase-seq, H3K4me3 ChIP-seq, and H3K27ac ChIP-seq 1kb around the detected off-target sites derived from different sgRNAs. The off-target

sites shared similar signal distribution across different sgRNAs. The normalized signal is calculated by (mean read counts within 1kb region around a given off-target site)/ (genome-wide mean read counts).

8. Expand the Discussion to place Tracking seq2 more clearly within the current landscape of off target detection and prediction tools, and to more thoroughly discuss its advantages and limitations relative to existing methods.

Reply:

Thank you for the suggestion. We have expanded the Discussion section to more clearly position Tracking-seq2 within the current landscape of off-target detection methods. We have added a detailed comparison with existing tools, discussing the technical advantages of Tracking-seq2, such as its high sensitivity and applicability to primary cells with low requirement of cells. Additionally, we have explicitly discussed the limitations of the method, including its time-dependent nature of the signal capture. The content is added at Line #314-347, as follows:

"Compared to existing methods, Tracking-seq2 offers distinct technical advantages. First of all, most off-target detection technologies require DSBs and are thus mainly confined to Cas9 system (e.g. GUIDE-seq[12]/GUIDE-seq-2[25], DISCOVER-seq[13]/DISCOVER-seq^{+}[14], and CIRCLE-seq[8]) and other editors require dedicated methods (e.g. Detect-seq[16] for CBEs). Besides, GUIDE-seq/GUIDE-seq-2 requires the integration of exogenous oligodeoxynucleotide at off-target sites, which may trigger apoptosis due to high levels of free DNA ends in fragile primary cells[33]. Furthermore, while DISCOVER-seq[13]/DISCOVER-seq^{+}[14] requires 1-5 million input cells, Tracking-seq2 operates with substantially lower cell inputs (as few as 50,000 cells)—a critical asset for scarce clinical samples. Unlike cell-free biochemical assays (e.g., CIRCLE-seq[8,] SITE-seq[10]) and in silico tools (e.g., Cas-OFFinder[24] and COSMID[26]), Tracking-seq2 functions within native chromatin contexts, thereby eliminating the vast number of false positives typically associated with naked DNA templates or simple sequence alignment. Our direct benchmarking against GUIDE-seq-2 and COSMID demonstrated that Tracking-seq2 achieves comparable, if not superior, sensitivity in primary T cells. Importantly, each method identified unique bona fide off-target sites missed by the others. This underscores that these technologies are complementary and that their combined use offers the most rigorous strategy for comprehensive clinical safety assessment.

Despite these strengths, several limitations of the current study warrant consideration. First, while Tracking-seq2 effectively identifies off-target cleavage or nicking events, it does not provide information about structural variations, such as large deletions or translocations. Second, the sensitized condition of T5/KU-60648 treatment may overestimate certain weak off-target cleavage/nicking events, which may not lead to detectable off-target editing at such sites under physiological conditions. Third, because the assay captures transient RPA-ssDNA intermediates, it is inherently constrained to specific temporal windows of editing and may overlook late-occurring off-target events beyond early sampling timepoints. Fourth, for CBEs, detection sensitivity remains suboptimal for certain weak off-target effects. The SSB repair cascades are currently poorly characterized and lack potent small-molecule inhibitors, hindering further signal amplification for base editors. We recommend Detect-seq as a complementary cell-based method tailored for CBEs. Finally, our study is restricted to in vitro and ex vivo systems, and the performance of Tracking-seq2 for in vivo off-target profiling deserves further investigation."

9. Consider whether the use of NHEJ inhibitors in "clinically relevant" cells introduces biases in off target spectra (e.g. shift to MMEJ/HR): does Tracking-seq2 truly reflect physiological off target behavior, or a modified repair landscape used only for detection?

Reply:

Thank you for raising this critical point regarding the physiological relevance of our detection conditions. We have clarified in the revised manuscript that our experimental workflow consists of two distinct parts:

Tracking-seq2 utilizes a two-step workflow: an initial "detection phase" using exonuclease and NHEJ inhibition to maximize sensitivity, followed by a "validation" phase using targeted deep sequencing under standard physiological conditions (without modulators) to confirm true off-target effects. We added a clearer workflow as Fig. 1a in the revised manuscript.

This two-step workflow is also required by many other off-target detection methods, such as GUIDE-seq, GUIDE-seq-2 and DISCOVER-seq^{+}. To ensure this workflow is entirely clear, we have explicitly illustrated the two-step process in the manuscript and, as suggested, have added a discussion in the Discussion section detailing its trade-offs and its advantages over single-step methods like Tracking-seq1.

[Figure Redacted]

Fig. R19 (Corresponding to Fig. 1a in the revised manuscript). Schematic illustration of the Tracking-seq2 workflow for Cas9 and Cas12a off-target detection. Exogenous T5 exonuclease treatment and NHEJ inhibition (using KU-60648) synergistically enhance the detection of Cas9 and Cas12a off-target sites by increasing the accumulation of RPA-bound ssDNA intermediates. The Tracking-seq2 workflow consists of two distinct phases: (1) Detection Phase—cells treated with T5 exonuclease and NHEJ inhibitor are used for the Tracking-seq2 assay to enrich ssDNA intermediates and maximize detection sensitivity; (2) Validation Phase—cells edited without T5 exonuclease and NHEJ inhibitor are used for targeted deep sequencing to measure actual off-target efficiency under physiological conditions.

10. I would suggest comparing Tracking-seq2 against other state of the art assays (e.g. DISCOVER Seq/+, ONE seq, GUIDE seq/2, CHANGE seq, SITE seq) on the same targets and in primary cells (at least another assay respect to the two mentioned here) to provide more robust findings.

Reply:

Thank you for this constructive suggestion. To provide more robust findings and benchmark our method against the current landscape, we have performed additional comparison of Tracking-seq2 against two other state-of-the-art methods, GUIDE-seq-2 (cell-based) and COSMID (in silico), in HEK293T cells and primary T cells.

In HEK293T cells, Tracking-seq2 demonstrated superior sensitivity. Specifically, for both HEK4 and VEGFA2 targets, Tracking-seq2 identified a substantial number of additional off-target sites

while capturing the vast majority of sites detected by GUIDE-seq-2 **(Fig. R20a)**. We conducted targeted amplicon sequencing for novel off-target sites that were not included in our hybrid capture data (i.e. unique novel sites from GUIDE-seq-2 or COSMID) and analyzed the distribution of Δindel rates (indel rates of Cas9-edited samples minus indel rates of WT samples) among different subsets of sites **(Fig. R20b)**. This result confirmed that Tracking-seq2 detected a lot of bona fide off-target sites missed by GUIDE-seq-2 or COSMID, while these two methods also provided several true off-target sites missed by Tracking-seq2.

In primary T cells, three methods shared common off-target sites and also included unique sites **(Fig. R20c).** To validate these unique sites, we performed amplicon sequencing on the top-10 unique sites specific to each method. The results showed that Tracking-seq2-only sites had higher Δindel rates than the others, while the other two methods also identified several genuine editing sites **(Fig. R20d)**.

According to these results, we recommend combining these technologies in clinical applications to ensure the most comprehensive safety assessment.

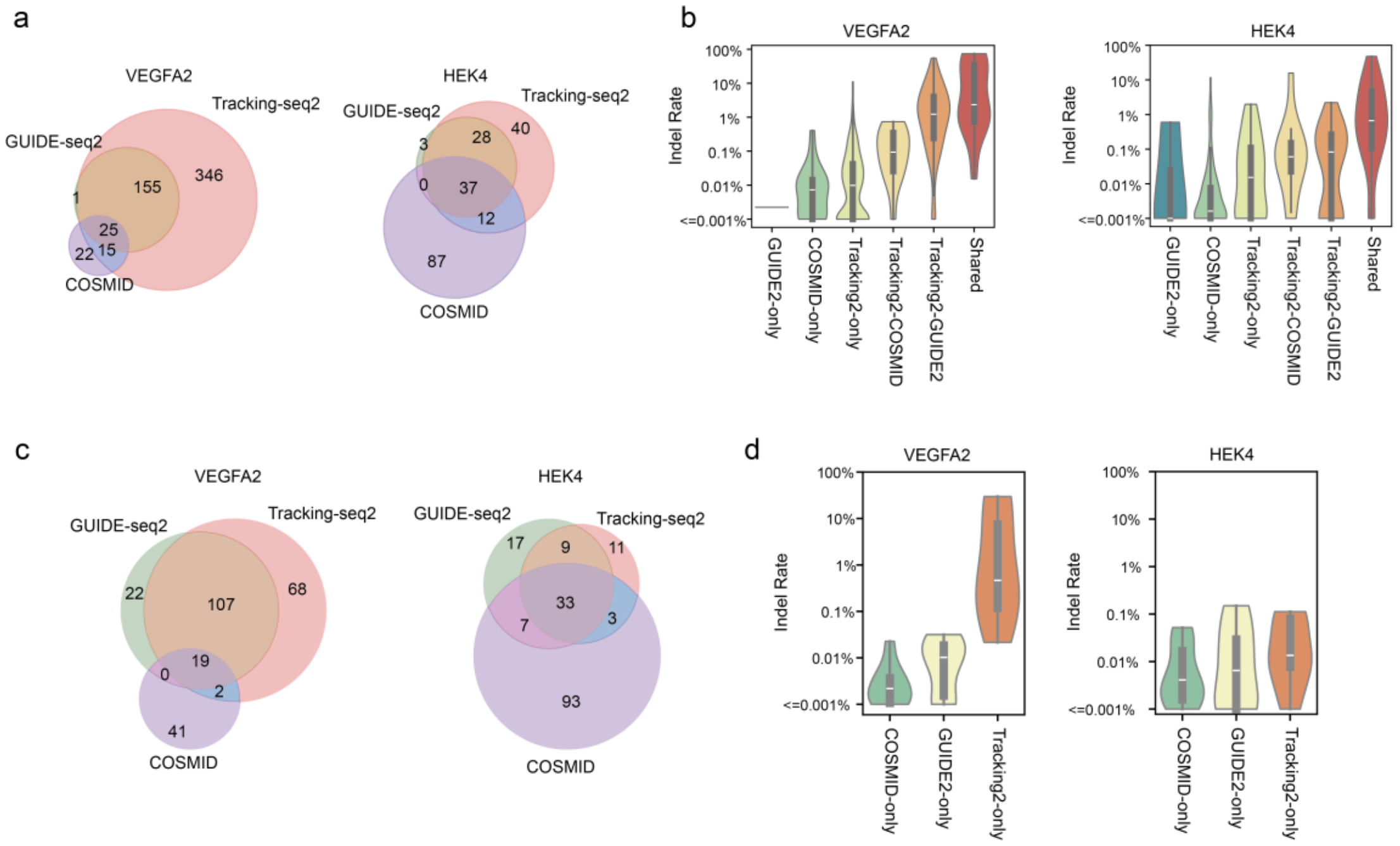

Fig. R20 (Corresponding to Fig. 2c-d and Fig. 4e-f in the revised manuscript). a, Venn diagrams comparing off-target sites detected (or predicted) by Tracking-seq2, GUIDE-seq-2, and COSMID for Cas9 + sgRNA VEGFA2 or Cas9 + sgRNA HEK4. **b**, Box plot showing the distribution of Δindel rates measured by hybrid capture targeted sequencing at off-target sites belonging to different sets for Cas9 + VEGFA2 and Cas9 + HEK4. GUIDE-seq-2 and COSMID missed a considerable number of off-target sites detected by Tracking-seq2. **c**, Venn diagrams comparing off-target sites detected (or predicted) by Tracking-seq2, GUIDE-seq-2, and COSMID for Cas9 + VEGFA2 or Cas9 + HEK4 in human T cells. **d**, Box plot showing the distribution of Δindel rates measured by targeted amplicon sequencing at method-specific off-target sites (top10 per method) for Cas9 + VEGFA2 or Cas9 + HEK4 in human T cells. Off-target sites exclusively identified by Tracking-seq2 exhibited the highest indel frequencies compared to those uniquely detected by GUIDE-seq-2 or COSMID.

11. You should mention if you menage to reduce the cell numbers required for Tracking-seq2 respect to other state of the art assays. If so, this is a major technical advance (e.g. "using low input cell numbers") to explicitly mention.

Reply:

Thank you for highlighting the importance of clarifying the cell input requirements. In the revised manuscript, we added experiments to compare the results from inputs of 10,000, 50,000, 100,000, and 500,000 cells in HEK293T and primary T cells. Compared to the 500,000-cell baseline, Tracking-seq2 with 50,000 or 100,000 cells resulted in only a slight reduction in detected off-target sites for both cell types **(Fig. R21)**. However, with an input of 10,000 cells, weak off-target signals became indistinguishable from background noise. Therefore, we recommend ≥50,000 cells for primary cells to balance sensitivity with sample availability. We have updated the Results sections in the revised manuscript to explicitly state these results and added this advance in the Discussion section as mentioned above.

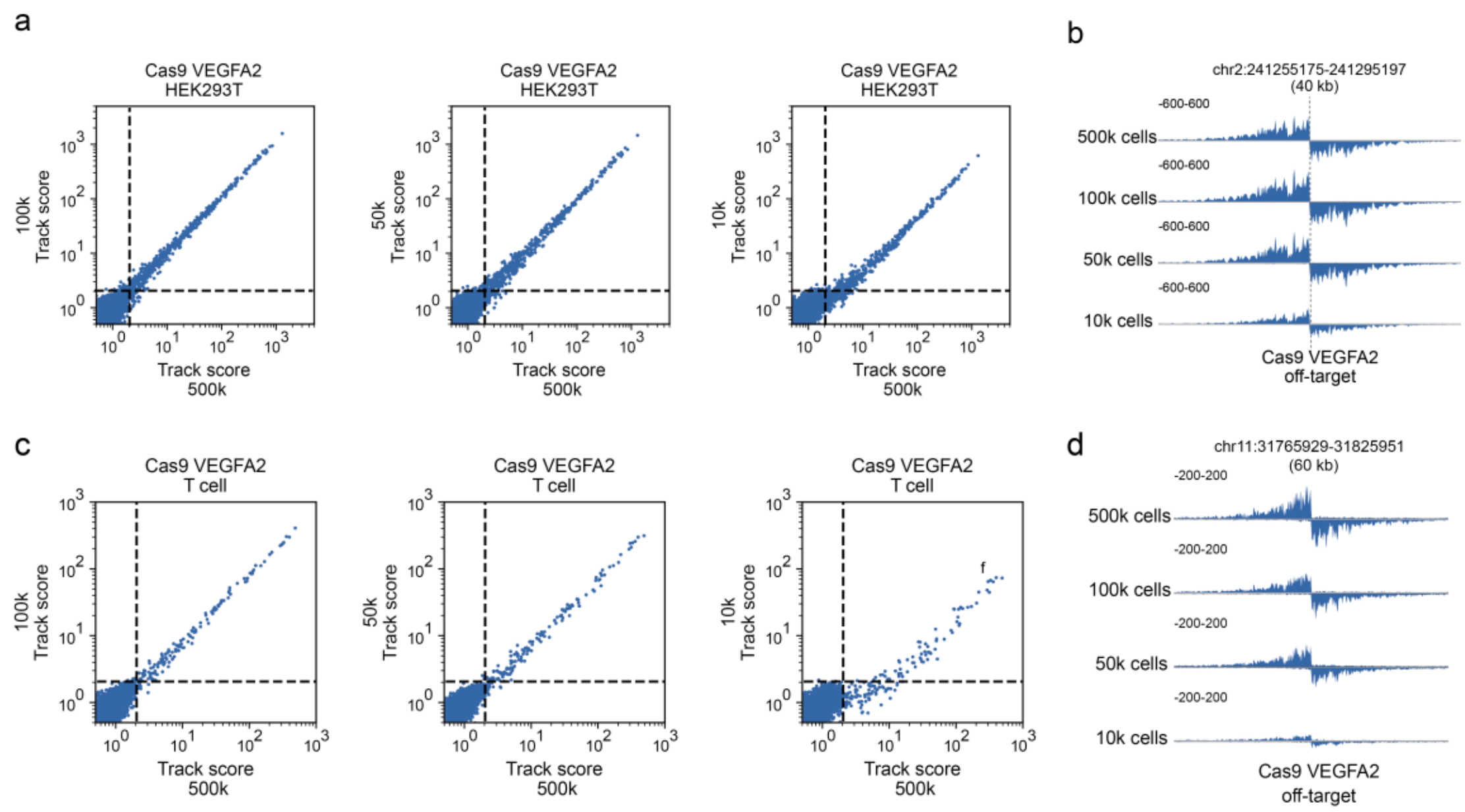

Fig. R21 (Corresponding to Extended Data Fig. 9 and Fig. 4g-h in the revised manuscript). a, Correlation between Track scores in experiments with different HEK293T cells (10,000, 50,000, 100,000, or 500,000 cells) edited by Cas9 + sgRNA VEGFA2. Each dot represents a candidate off-target site. Dashed lines represent the thresholds of the Track score > 2 for positive sites. Sensitivity with 100,000 or 50,000 cells was similar to that with 500,000 cells, while assays using 10,000 cells missed off-target sites with low signals. **b**, Tracking-seq signals at a representative off-target site using different numbers of HEK293T cells. **c,** Correlation between Track scores in experiments with varying human T cell inputs (10,000, 50,000, 100,000, and 500,000 cells). Each dot represents a candidate off-target site. Sensitivity with 100,000 or 50,000 cells was only slightly lower than that with 500,000 cells, while assays using 10,000 cells missed off-target sites with low signals. **d**, Tracking-seq2 signals at a representative off-target site using different numbers of human T cells.

12. Are these differences linked to specific variants at off target sites (e.g. SNVs/indels that create or destroy PAMs/mismatches)?

Reply:

Thank you for this insightful question regarding the genetic variants of the observed differences. For a subset of the differential off-target sites, we found that the differences were indeed linked to specific variants **(Fig. R22)**.

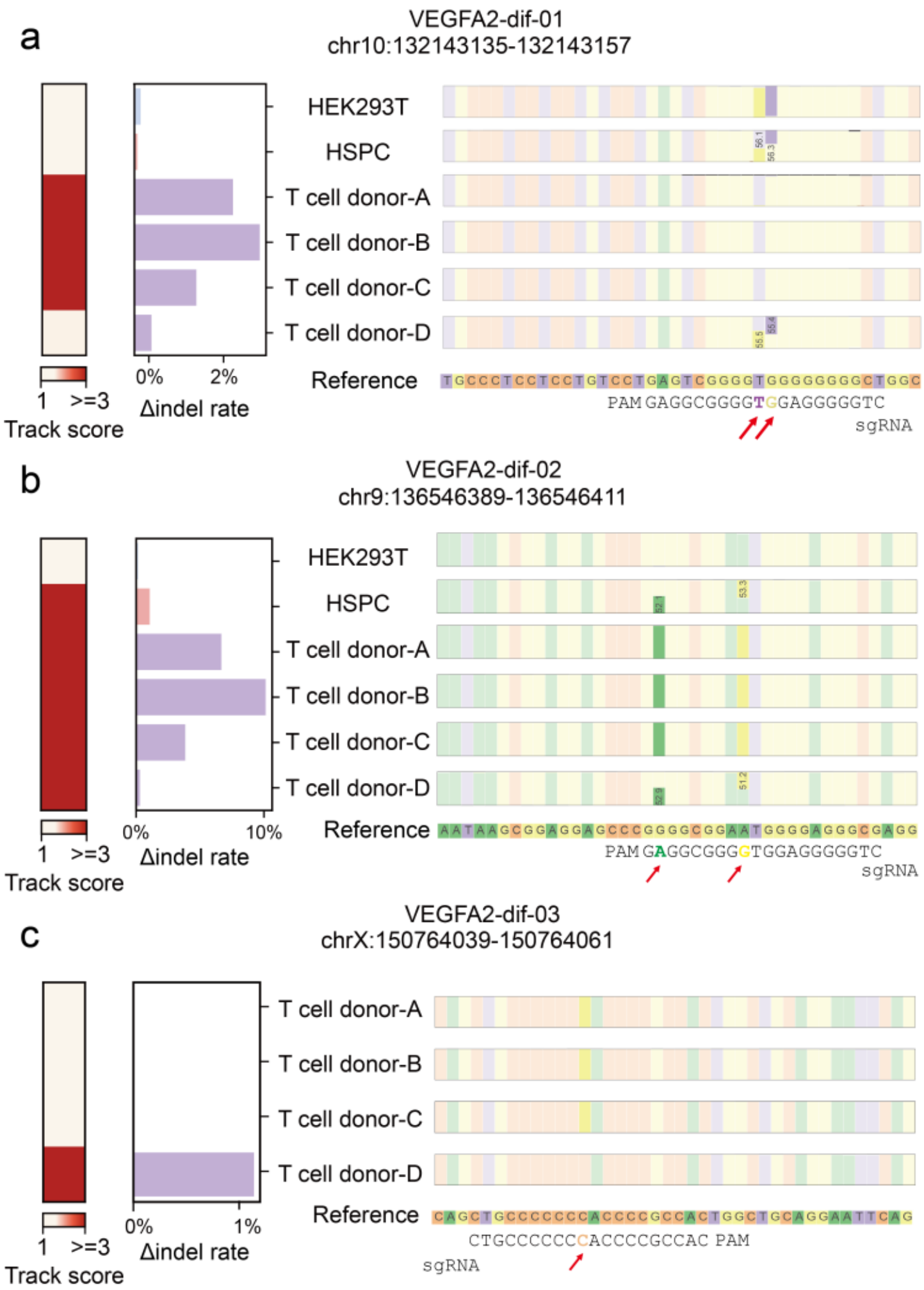

Fig. R22 (Corresponding to Fig. 5e-g in the revised manuscript). a-c, Genomic sequences of unedited HEK293T cells, HSPCs, and T cells from four different donors at the identified differential off-target sites for the sgRNA VEGFA2, as determined by targeted amplicon sequencing. For the VEGFA2-dif-01 locus (**a**), T cells from donors A, B, and C harbored a genomic sequence identical to the hg38 reference, which matched the sgRNA at the marked two nucleotides. In contrast, T cells from donor D and the HSPCs were heterozygous at these two nucleotides, while HEK293T cells were homozygous for the variant, both resulting in mismatches with the sgRNA sequence. For VEGFA2-dif-02 (**b**), T cells from donors A, B, and C contained two homozygous nucleotide polymorphisms that matched the sgRNA sequence, whereas HEK293T cells were homozygous at these two nucleotides that mismatched the sgRNA sequence. HSPCs and T cells from donor D were heterozygous at these positions, resulting in weak off-target editing. For VEGFA2-dif-03 (**c**), T cells from donors A, B, and C contained a homozygous nucleotide polymorphism that mismatched the sgRNA sequence, while T cells from donor D matched the nucleotide.

Results section:

Mechanistic section (T5 + NHEJ inhibition)

- Strength: They nicely demonstrate that overexpressed T5 exonuclease and NHEJ inhibition (KU 60648) each increase the number and amplitude of RPA ssDNA signals, and that the combination yields ~2–2.5× more Cas9 and ~5× more Cas12a off targets versus the original Tracking seq in HEK293T cells. This directly supports their technical claim of improved sensitivity. The points I would like to raise are:

13. overexpression of a bacterial exonuclease and pharmacological NHEJ inhibition substantially perturb the repair landscape. The authors should explicitly discuss to what extent the resulting off target profile reflects a "physiological" spectrum versus a sensitized, detection optimized context, especially for sites with marginal activity.

Reply:

Thank you for pointing out this concern regarding the physiological relevance. As described in the response to the NHEJ inhibitors condition above (comment #9), all the validations of off-target editing were performed using cells without additional treatment (i.e., reflect physiological spectrum of off-targets). Although false positives existed, a considerable proportion of the additional off-target sites detected by Tracking-seq2 were validated to be true off-target sites in the physiological condition, underscoring the value of Tracking-seq2 for genome-editing scenarios with high requirement of safety assessment.

14. is the increased number of sites accompanied by a shift in indel spectra or microhomology usage at on and off targets (i.e. evidence of more MMEJ)? Did you check for increased cytotoxicity or global DNA damage?

Reply:

Thank you for raising these important questions. However, all validations of off-target editing were performed with cells in physiological condition, thus the indel spectra under T5 or NHEJ inhibitor treatments were not characterized. As the treated cells were only used for Tracking-seq2 in Detection Phase, we did not check the cytotoxicity or global DNA damage in the treated cells.

15. for base and prime editors, only T5 (no KU 60648) is used. It would be helpful if the authors comment on why NHEJ inhibition is not applied there (e.g. not relevant for SSB based pathways, or toxicity/artefacts).

Reply:

Thank you for this question. We have added a description in the revised manuscript explaining the rationale for this experimental design in the Introduction section. As you mentioned, BEs and PEs generate SSBs, while NHEJ pathway is primarily involved in DSB repair. Therefore, NHEJ inhibition is not applied for BEs and PEs.

Comparison to CIRCLE seq / Cas OFFinder

- Strength: The authors validate >5,000 candidate sites by capture seq and show that the majority of Tracking seq2 only or Tracking seq2 shared sites have measurable indel frequencies, whereas many CIRCLE seq/Cas OFFinder only sites do not. This supports a good precision/specificity argument for Tracking seq2 in cells. The points I would like to raise are:

16. Sensitivity claims are relative and incomplete. They only compare against one in vitro assay (CIRCLE seq) and one in silico tool, not against other modern cell based or in situ methods (GUIDE seq/2, DISCOVER seq/+, GUIDE tag, CHANGE seq, AID seq, etc.), several of which are known to detect high mismatch and non NGG sites. I would suggest softening statements like "high specificity and sensitivity" and especially any "most sensitive" wording, or adding direct benchmarks to at least one other state of the art cellular assay if feasible.

Reply:

Thank you for this important feedback. We completely agree that our initial comparisons were insufficient to fully support our sensitivity claims in a cellular context. We have carefully revised the manuscript to soften our statements and removed absolute phrases such as "most sensitive" and carefully modulated our wording to accurately reflect the results of our experiments.

Additionally, to directly address this gap, we have performed extensive new experiments to benchmark Tracking-seq2 against GUIDE-seq-2 and COSMID, as detailed in our response to the previous point (comment #10).

17. The conclusion that cell free/computational methods "miss substantial true off target sites" is likely correct in principle, but here it rests on one guide pair and one cell line. I would extend the comparison to additional guides and/or editors.

Reply:

Thank you for pointing out the limitation. We agree that our initial conclusion was somewhat overstated, as the substantial number of missed off-target sites was primarily observed with the VEGFA2 sgRNA, whereas the effect was less pronounced for HEK4. Consequently, we have softened the description to avoid overgeneralization. We now state that cell free/computational methods may miss true off-target sites that can be detected by Tracking-seq2. We believe this revision ensures that our conclusions are strictly supported by the presented data.

Application to BEs and PEs

- Strength: They demonstrate that T5 also boosts signals for CBE, ABE, and PE3, yielding ~3× more CBE off targets (VEGFA2), ~1.5× for HEK4, while PEs remain relatively high fidelity but gain some additional low frequency sites. This supports the claim that Tracking seq2 generalizes to SSB based editors. The points I would like to raise are:

18. Please explicitly report editing frequencies (not just "novel sites") and a clearer definition of what threshold you used to call an off target for base edits versus indels. It is not clear from the methods.

Reply:

Thank you for this important feedback. We performed additional targeted amplicon sequencing on top five novel sites for each condition of BEs and PEs (CBE-VEGFA2, CBE-HEK4, ABE-VEGFA2, ABE-HEK4, PE3-VEGFA2, and PE3-HEK4). For CBE and ABE, all tested sites exhibited C to T or A to G editing when C or A bases were present within the editing window **(Fig. R23a-d)**. For 4 sites of ABE lacking an A in the editing window, low-frequency indels were detected in 3 of them **(Fig. R23b)**. For PE3, we evaluated all types of substitutions and indels for off-target editing, but the majority exhibited no significant editing **(Fig. R23e)**. Given that only three novel off-target sites were identified for PE3-HEK4, we additionally validated four sites jointly detected by both

Tracking-seq V1 and V2. The results revealed that only the site with the strongest Tracking-seq2 signal (V1&V2-01) contained substantial off-target editing. Considering the presence of distinct Tracking-seq2 signals at mutation-negative sites **(Fig. R23f)**, we hypothesize that while DNA nicking events do occur at these loci, they are efficiently repaired in an error-free manner. Our findings are consistent with prior reports demonstrating that PE exhibited high fidelity with much lower off-target effects.

Besides, we have updated the Methods section to clearly define the off-target editing for different editors: For Cas9, we regard indels within ±1 bp of the cleavage position (3 bp upstream the PAM) as off-target editing; for ABE, we regard A-to-G conversion within the 20 bp sgRNA range and indel ±1 bp around the cleavage position as off-target editing; for CBE, we regard C-to-T conversion within the 20 bp sgRNA range and indel ±1 bp around the cleavage position as off-target editing; for PE, we regard all types of substitutions and indel ±10 bp around the 5′ of PAM as off-target editing.

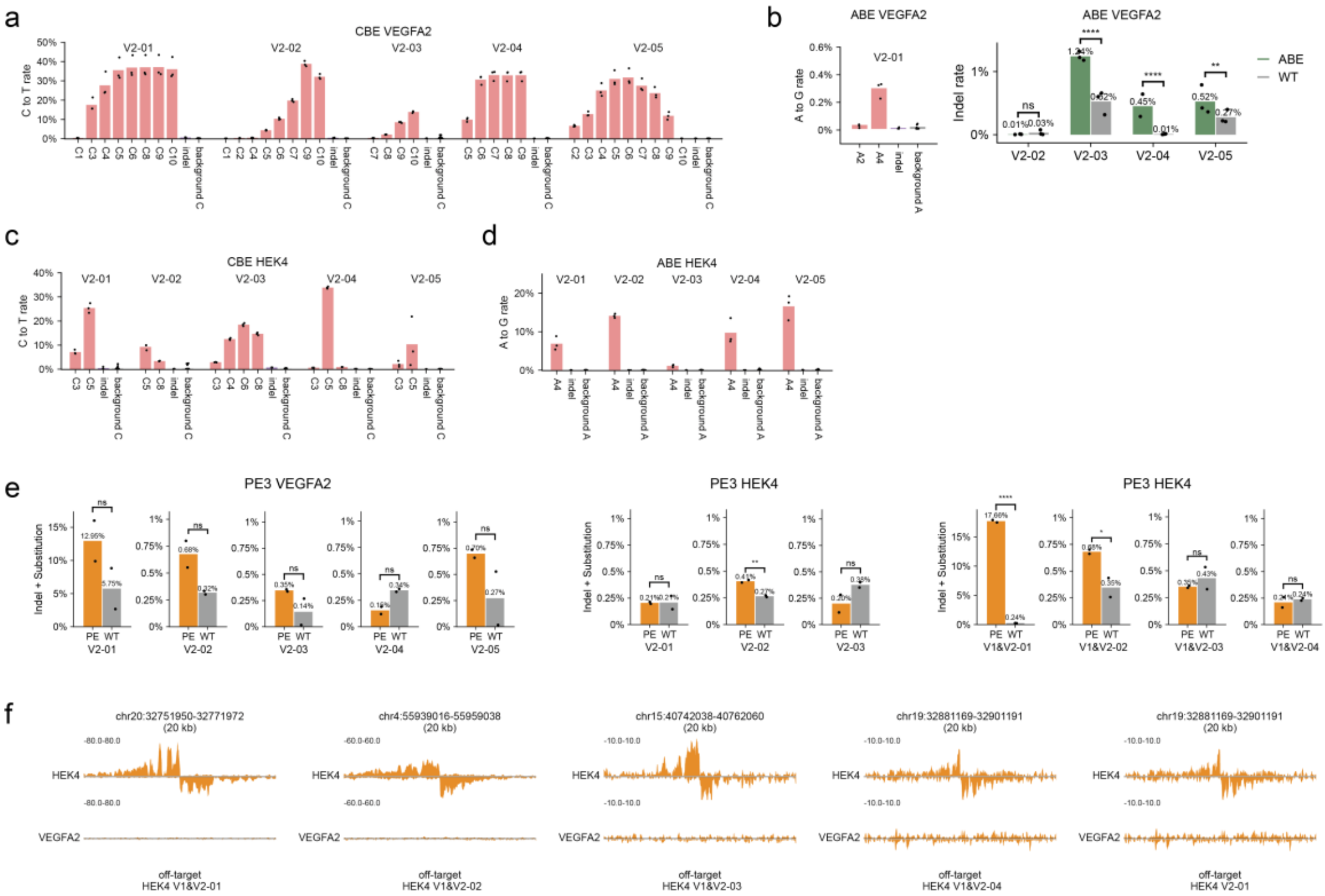

Fig. R23 (Corresponding to Extended Data Fig. 7 in the revised manuscript). a, Scatter plot showing the Track scores of Tracking-seq with combined T5 co-transfection and KU-60648 treatment versus those without treatment edited by Cas9 + sgRNA VEGFA2 or HEK4. Each dot represents a candidate off-target site. Dashed lines represent the thresholds of the Track score > 2 for positive sites. V1: Tracking-seq1. **b**, Box plot showing the distribution of indel rates measured by hybrid capture targeted sequencing at off-target sites exclusively detected by Tracking-seq2 and shared by Tracking-seq and Tracking-seq2 for sgRNA VEGFA2 or sgRNA HEK4. Approximately half of the additional off-target sites detected by Tracking-seq2 were validated as true off-target sites in the physiological condition (without T5 or KU-60648 treatment). **c-f**, Targeted amplicon sequencing validation of the top five novel off-target sites detected by Tracking-seq2 for CBE + sgRNA VEGFA2 (a), ABE + sgRNA VEGFA2 (b), CBE + sgRNA HEK4 (c), and ABE + sgRNA

HEK4 (d) in HEK293T cells. All tested sites exhibited C-to-T editing within the editing window for CBE + sgRNA VEGFA2 or HEK4. For ABE + sgRNA VEGFA2 or HEK4, all tested sites with adenines within the editing window exhibited A-to-G editing, while 3/4 sites lacking adenines in the editing window exhibited low-frequency indels. *P*-values were calculated by one-sided binomial tests and the exact value for each site is listed in Supplementary Table 3. Background C/A: C or A outside the editing window. **g**, Targeted amplicon sequencing validation of off-target sites for PE3. Novel off-target sites for PE3 + pegRNA VEGFA2 (left) and for PE3 + pegRNA HEK4 (middle) and four sites jointly detected by both Tracking-seq V1 and V2 for PE3 + pegRNA HEK4 (right). The majority of tested sites exhibited no significant editing, consistent with the high fidelity of prime editors. Only the site with the strongest Tracking-seq2 signal (V1&V2-01) contained substantial off-target editing. *P*-values were calculated by one-sided binomial tests and the exact value for each site is listed in Supplementary Table 3. **h**, Tracking-seq2 signals at representative off-target sites for PE3 + pegRNA HEK4. PE3 + pegRNA VEGFA2 serves as a non-targeting control. Distinct signals were observed at these off-target sites lacking detectable mutations, suggesting that DNA nicking events occurred at these loci but are efficiently repaired in an error-free manner.

19. Given that PE3 is already known to be specific, it would be good if the authors show that T5 does not generate spurious "off targets" in unedited controls for these modalities, similarly to what they did for Cas9.

Reply:

Thank you for this important suggestion. To ensure that T5 overexpression does not induce spurious off-target signals, we have added a negative control experiment where we performed Tracking-seq2 on unedited cells treated with T5 exonuclease only **(Fig. R24)**. The results confirm that no detectable off-target signals were detected in this control group. This demonstrates that T5 alone does not generate background noise or false positives, thereby validating the specificity of the signals observed for BEs and PE3.

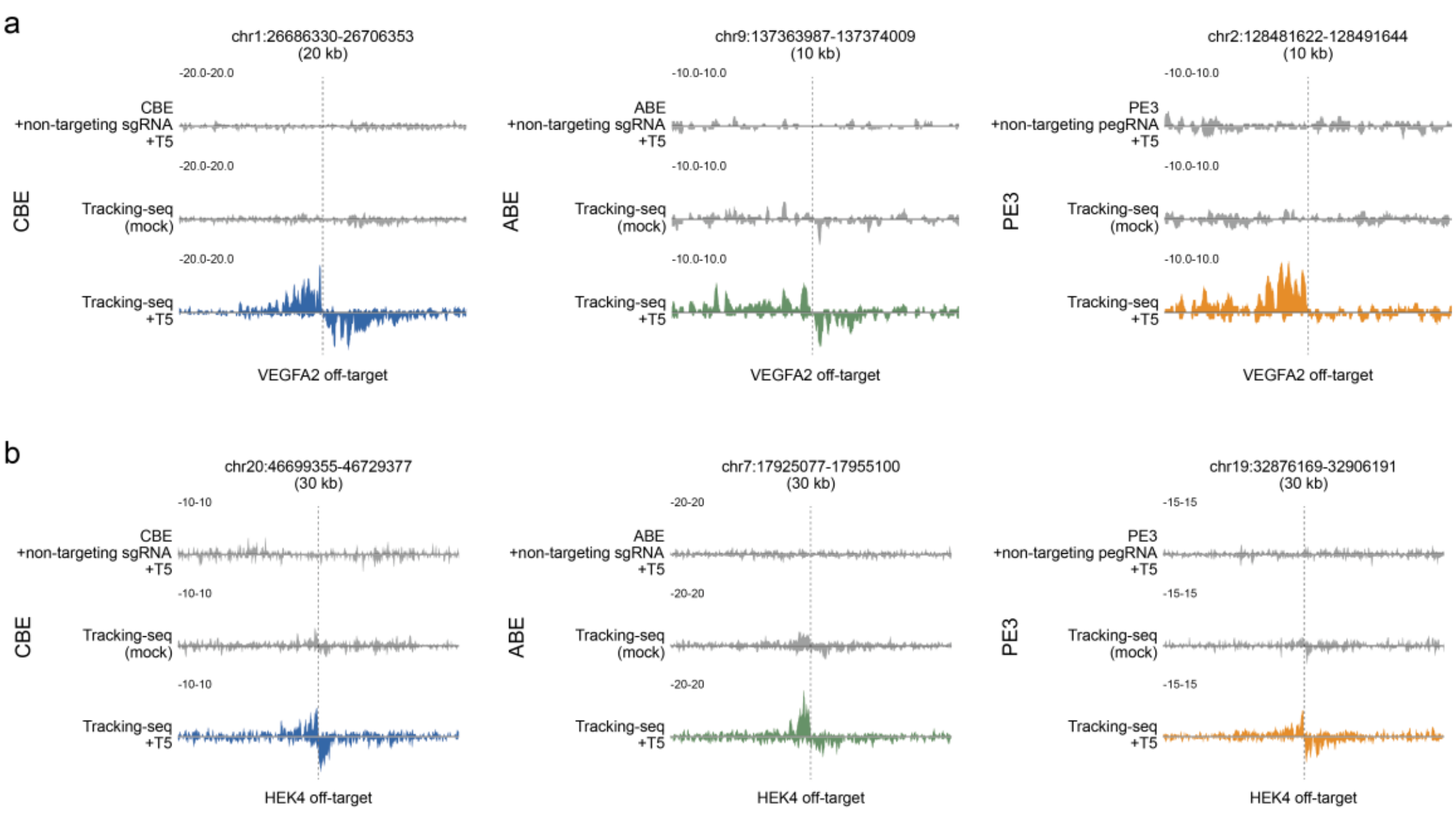

Fig. R24 (Corresponding to Fig. 3d and Extended Data Fig. 6d in the revised manuscript). a, Signals generated by Tracking-seq at representative off-target sites of sgRNA/pegRNA VEGFA2.

b, Signals generated by Tracking-seq at representative off-target sites of sgRNA/pegRNA HEK4. The range in the top left of each track denotes normalized signal intensity, represented as read coverage per 10 million mapped reads.

Primary cells and "personalized" heterogeneity

- Strength: They extend Tracking seq2 to RNP edited primary T cells and CD34+ HSPCs and show ~2–3× more sites vs original Tracking seq, with some sites shared and some unique per cell type. That is a clear practical advance and directly relevant to clinical applications. The points I would like to raise are:

20. Heterogeneity across cell types (HEK293T vs T vs HSPC) is not the same as inter individual heterogeneity. Here the authors show that a few off targets are unique to T cells or HSPCs and validate them by amplicon sequencing. Later, they link some differential sites to sequence polymorphisms and also note sites with identical sequence but differential activity, implying chromatin or other factors.

Reply:

Thank you for this insightful comment. We agree that heterogeneity across cell types is not the same as inter-individual heterogeneity. We have performed additional experiments using primary T-cells to include a total of four distinct donors in the study, as described in the response to comment #12. These new data strengthen the clinical relevance of Tracking-seq2 for assessing donor-specific off-target risks.

Discussion section:

The current Discussion is very short and reads as promotional rather than balanced. It does not sufficiently position Tracking seq2 in the rapidly evolving landscape of off target detection and prediction tools, nor does it clearly articulate the limitations of the present study (limited benchmarking against other cell based assays, absence of HDR/in vivo data, small number of donors, use of T5 and NHEJ inhibition). I recommend substantially expanding and revising this section to:

21. compare Tracking seq2 to existing cell based and in vitro assays and to in silico prediction tools;

Reply:

Thank you for the constructive criticism regarding the Discussion section. We agree that the original version was too brief and lacked sufficient context. We have substantially expanded and revised the Discussion to provide a more balanced and comprehensive view. The comparison between Tracking-seq2 and other methods are at Line #314-332 in the revised manuscript as follows:

"Compared to existing methods, Tracking-seq2 offers distinct technical advantages. First of all, most off-target detection technologies require DSBs and are thus mainly confined to Cas9 system (e.g. GUIDE-seq[12]/GUIDE-seq-2[25], DISCOVER-seq[13]/DISCOVER-seq+[14], and CIRCLE-seq[8]) and other editors require dedicated methods (e.g. Detect-seq[16] for CBEs). Besides, GUIDE-seq/GUIDE-seq-2 requires the integration of exogenous oligodeoxynucleotide at off-target sites, which may trigger apoptosis due to high levels of free DNA ends in fragile primary cells[33]. Furthermore, while DISCOVER-seq[13]/DISCOVER-seq+[14] requires 1-5 million input cells, Tracking-seq2 operates with substantially lower cell inputs (as few as 50,000 cells)—a critical asset for scarce clinical samples. Unlike cell-free biochemical assays (e.g., CIRCLE-seq[8,] SITE-seq[10]) and in silico tools (e.g., Cas-

OFFinder[24] and COSMID[26]), Tracking-seq2 functions within native chromatin contexts, thereby eliminating the vast number of false positives typically associated with naked DNA templates or simple sequence alignment. Our direct benchmarking against GUIDE-seq-2 and COSMID demonstrated that Tracking-seq2 achieves comparable, if not superior, sensitivity in primary T cells. Importantly, each method identified unique bona fide off-target sites missed by the others. This underscores that these technologies are complementary and that their combined use offers the most rigorous strategy for comprehensive clinical safety assessment."

22. moderate claims of 'unprecedented sensitivity' and 'overcoming a major barrier' unless supported by broader head to head data;

Reply:

Thank you for this advice. We agree that these statements were too strong given the current scope of benchmarking. We have removed claims of "unprecedented sensitivity" and "overcoming a major barrier" from the revised manuscript to ensure our conclusions are objective.

23. temper the language around 'personalized off target assessment' and clearly distinguish what is demonstrated here (donor/cell type heterogeneity in vitro) from future, clinically oriented applications;

Reply:

Thank you for this suggestion. We have revised the discussion to temper the language regarding "personalized off-target assessment." We now clearly distinguish between what is demonstrated in this study—specifically, the detection of off-target heterogeneity across different cell types and donors—and the future potential for clinical application. We have modified the text to state that Tracking-seq2 serves as a powerful technology for potential patient-specific risk evaluation.

24. explicitly acknowledge method specific limitations, including the perturbed repair context and lack of long term/in vivo data."

Reply:

Thank you for this advice. We have added a dedicated paragraph in the Discussion section to explicitly acknowledge the limitations of Tracking-seq2 at Line #333-347 in the revised manuscript as follows:

"Despite these strengths, several limitations of the current study warrant consideration. First, while Tracking-seq2 effectively identifies off-target cleavage or nicking events, it does not provide information about structural variations, such as large deletions or translocations. Second, the sensitized condition of T5/KU-60648 treatment may overestimate certain weak off-target cleavage/nicking events, which may not lead to detectable off-target editing at such sites under physiological conditions. Third, because the assay captures transient RPA-ssDNA intermediates, it is inherently constrained to specific temporal windows of editing and may overlook late-occurring off-target events beyond early sampling timepoints. Fourth, for CBEs, detection sensitivity remains suboptimal for certain weak off-target effects. The SSB repair cascades are currently poorly characterized and lack potent small-molecule inhibitors, hindering further signal amplification for base editors. We recommend Detect-seq as a complementary cell-based method tailored for CBEs. Finally, our study is restricted to in vitro and ex vivo systems, and the performance of Tracking-seq2 for in vivo off-target profiling deserves further investigation."

Point-by-point responses to the reviewers' comments

Reviewer #1

(Remarks to the Author):

The authors have carefully and comprehensively addressed the prior comments. The revisions have improved the clarity, rigor, and overall impact of the work. I appreciate the thoughtful responses and believe the manuscript is now suitable for publication.

Reply:

Thank you very much for your positive evaluation of our revised manuscript. Your constructive suggestion has profoundly strengthened our study. We are very glad that our revisions have met your expectations.

Reviewer #2

(Remarks to the Author):

All my comments were addressed and I recommend the manuscript for publication

Reply:

We deeply appreciate your thorough evaluation and constructive feedback, which have greatly helped us improve the manuscript. We are very glad that you are satisfied with our revised manuscript.

Reviewer #4

(Remarks to the Author):

Dr. Cong and colleagues developed Tracking-seq2, an advanced technology that integrates 5′→3′ exonuclease treatment and inhibitors of the non-homologous end joining (NHEJ) pathway with the original Tracking-seq. The overall concept is interesting, but the revised manuscript is still not yet ready for publication in Nature Communications. As the authors note in their rebuttal, they have responded to most concerns raised by the four reviewers, including performing:

- Benchmarking against additional state-of-the-art methods

- Investigation of donor-specific heterogeneity

- Evaluation of TadA-derived CBEs

- Assessment of HiFi Cas9

- Additional targeting of new loci

- Comprehensive amplicon sequencing validation

- Increased biological replicates

- Expansion of the Introduction

- Comprehensive discussion of methodology and limitations

- Refinement of the Methods section

Although most reviewer points were addressed, a few important issues included in my first revision remain that I believe should be resolved to bring the study in line with the journal's standards. I list them below; for several I understand the practical reasons for omission and accept them, but I identify one experiment that I think should be added as a minor revision.

1) Biological replicates (increase to n = 5). The authors state they agreed with the handling editor that n = 3 is acceptable for this type of NGS assay. Given the substantial cost, labor, and revision time constraints, I accept this compromise.

2) In vivo repopulation assay to evaluate selection/expansion of edited clones. The authors did not perform such an experiment. They have, however, discussed the limited temporal detection window of Tracking-seq2 as a limitation in the revised Discussion. I accept this justification.

3) Off-target detection in the context of HDR using different donor templates (AAV6 vs. ssODN). The authors did not address this; I acknowledge that this likely requires a dedicated study and accept its omission here.

4) Comparison of CB-derived HSPCs to mobilized peripheral blood (mPB)-derived HSPCs. The authors did not include this comparison and explain that obtaining mPB HSPCs is challenging. They nonetheless provide convincing evidence validating Tracking-seq2's technical sensitivity on CB-derived HSPCs. I accept this limitation.

5) Cytotoxicity and global DNA-damage assessment following T5 or NHEJ inhibitor treatment. The authors did not assess cytotoxicity or global DNA damage in treated cells. I consider this an important omission. I recommend they perform these assays (for example, viability/apoptosis assays and γH2AX or comet assays) at least in n = 3 biological replicates using CB-derived HSPCs. I view this as a reasonable minor revision that would align the manuscript with Nature Communications' quality standards.

Overall recommendation: conditional acceptance after minor revision to include cytotoxicity and global DNA damage assays (point 5). The other omissions are acceptable with the authors' provided explanations and discussion.

Reply:

We are grateful to the reviewer for acknowledging the extensive revisions we have made, and we sincerely thank you for your understanding and acceptance of our previous justifications regarding points 1 to 4. Your insightful critiques have profoundly strengthened the robustness and clarity of our study.

Regarding the overall recommendation (related to point 5), we thank the reviewer for raising it. We completely agree that cytotoxicity and global DNA damage are crucial considerations for genome editing. However, the drug-treated cells are used strictly for off-target detection by Tracking-seq2 during the "detection phase" and do not affect the safety of the actual editing applications. Crucially, all validations of off-target results (by targeted hybrid capture sequencing or amplicon sequencing) were performed with untreated cells under standard physiological conditions. Therefore, the potential cytotoxicity or global DNA damage induced by the treatment does not compromise our final validated conclusions, making extensive toxicity testing in HSPCs of limited value.

Nevertheless, this conceptual point must be made clear to the readers. We have now revised the Discussion section for the potential cytotoxicity and DNA damage of the treatment, explicitly clarifying why it does not compromise the method's utility as follows (Lines #299-315):

"Tracking-seq2 employs a two-step workflow to balance detection sensitivity with physiological relevance. The first "detection phase" utilizes T5 exonuclease and NHEJ inhibitors to perturb the repair landscape and amplify RPA-ssDNA signals, thereby maximizing the discovery of low-frequency off-target sites. Although the treatment may induce a certain degree of cytotoxicity, the treated cells are used exclusively for Tracking-seq2 and are not intended for practical editing scenarios. As this treatment amplifies RPA-ssDNA intermediates and may overestimate rare off-target events, the second "validation phase" leverages targeted amplicon or hybrid capture sequencing under standard physiological conditions (without T5 or KU-60648) to accurately measure true off-target editing efficiencies. While the signal amplification during the detection phase alters the native repair environment and may cause additional DNA damage, our validation phase confirmed that a considerable amount of the newly identified candidates represented genuine off-target events under physiological conditions. Although this two-step paradigm increases experimental complexity, it is also required by other state-of-the-art methods (e.g., GUIDE-seq[12]/GUIDE-seq-2[25], DISCOVER-seq+[14]). More importantly, this trade-off yields markedly enhanced sensitivity over the original Tracking-seq, fully justifying the additional effort."
